# Associations of parental age with offspring all-cause and cause-specific adult mortality

**DOI:** 10.1038/s41598-019-52853-8

**Published:** 2019-11-19

**Authors:** David Carslake, Per Tynelius, Gerard J. van den Berg, George Davey Smith

**Affiliations:** 1 MRC Integrative Epidemiology Unit at the University of Bristol, Bristol, UK; 2 Population Health Sciences, Bristol Medical School, Bristol, UK; 3 Department of Public Health Sciences, Karolinska Institute, Stockholm, Sweden; 4 School of Economics, Finance and Management, University of Bristol, Bristol, UK

**Keywords:** Risk factors, Medical research

## Abstract

People are having children later in life. The consequences for offspring adult survival have been little studied due to the need for long follow-up linked to parental data and most research has considered offspring survival only in early life. We used Swedish registry data to examine all-cause and cause-specific adult mortality (293,470 deaths among 5,204,433 people, followed up to a maximum of 80 years old) in relation to parental age. For most common causes of death adult survival was improved in the offspring of older parents (HR for all-cause survival was 0.96 (95% CI: 0.96, 0.97) and 0.98 (0.97, 0.98) per five years of maternal and paternal age, respectively). The childhood environment provided by older parents may more than compensate for any physiological disadvantages. Within-family analyses suggested stronger benefits of advanced parental age. This emphasises the importance of secular trends; a parent’s later children were born into a wealthier, healthier world. Sibling-comparison analyses can best assess individual family planning choices, but our results suggested a vulnerability to selection bias when there is extensive censoring. We consider the numerous causal and non-causal mechanisms which can link parental age and offspring survival, and the difficulty of separating them with currently available data.

## Introduction

In the developed world, the average age at which people have children is increasing dramatically^1^. There are numerous mechanisms, sometimes anticipated to act in opposing directions, by which this rise in parental age could affect the health and well-being of their offspring. Children of older parents are probably more vulnerable to germline DNA mutations (particularly with older fathers^2^ but see^3^) and aneuploidy (particularly with older mothers^4^). The quality of the intra-uterine environment may decline with maternal age, and those with older parents are more likely to suffer the emotional and potentially financial hardship of an early bereavement^5^. On the other hand, the greater life experience and wealth of older parents may allow them to provide a more stable and well-resourced environment for their offspring^6^. Finally, in a country with improving living conditions delaying parenthood means that one’s children are born into a healthier, better-resourced society^7^.

Most studies so far of parental age effects on offspring survival have focused on the neonatal and early childhood periods. The lifelong consequences of parental age for the offspring are harder to study because of the shortage of large, unbiased, comprehensive datasets linking parental age and longevity. Most studies have been low-powered, lacked covariate data, and/or considered only simplified outcomes such as whether or not a person lived to a particular age. Extensive potential for confounding and co-linearity with variables such as birth order, the other parent’s age and (particularly within families) the offspring’s date of birth (DOB) make it difficult to identify causal effects of parental age and harder still to break them down into separate mechanisms.

One recent publication^7^ used the Swedish multi-generation register, linked to other national registers, to provide population-scale data on parental age and all-cause mortality for offspring aged up to 74 years, including data on family structure and socioeconomic position (SEP). Using the same data source, we extend this analysis to estimate parental age effects on cause-specific mortality and consider further the various causal and non-causal links behind crude parental age-mortality associations.

## Results

There were 293,470 deaths during 97,608,449 person-years of follow-up of the 5,204,433 offspring in the main analysis. The age during follow-up ranged between 18 and 80.75 years, with a mean of 43.1. Median maternal age was 27.5 (IQR 23.75 to 31.75), median paternal age was 30.5 (IQR 26.5 to 35.25), and the correlation between them was 0.76.

The offspring of younger mothers or fathers were born smaller and were slightly more likely to be male (Tables 1 and 2). At age 18 (data from sons only), they were shorter, had higher body mass index (BMI) but lower blood pressure, had lower intelligence, and were more likely to smoke. Offspring of younger parents had lower occupational and educational SEP. The overall trends for SEP, birth length, height, intelligence and non-cognitive ability were slightly reversed among offspring of the oldest fathers and, more so, of older mothers. Non-cognitive ability was highest at intermediate parental ages, falling considerably at the extremes. The occupational and educational SEP of the parents showed a similar pattern. The associations of these characteristics with parental age in this dataset have been considered at greater length elsewhere^8^.

**Table 1:** Description of the study population by classes of maternal age. Analyses were restricted to those offspring included in the primary analyses of cause-specific mortality. Within each class of maternal age, continuous variables were summarised as means, binary variables as percentages. P values tested for heterogeneity in each variable between the classes of maternal age. They came from unadjusted linear or logistic regressions with robust standard errors clustered by the mother’s identity. Intelligence and non-cognitive ability were each recorded on a scale of 1-9.

Person, variable	Mother’s age at offspring birth						P	N
	≤19	20–24	25–29	30–34	35–39	≥ 40		
***Mothers***								
Age at offspring birth (years)	18.6	22.6	27.3	32.1	37.0	42.0	<0.001	5,204,433
Non-manual worker	34.4%	39.1%	46.2%	40.9%	29.0%	17.6%	<0.001	4,590,785
Completed secondary school	9.0%	13.3%	22.3%	24.3%	20.9%	15.2%	<0.001	3,921,575
Alive when offspring 16	99.3%	99.3%	99.1%	98.6%	97.7%	96.2%	<0.001	3,542,302
Alive when offspring 40	93.6%	92.8%	90.5%	85.3%	76.1%	61.3%	<0.001	2,147,035
***Fathers***								
Age at offspring birth (years)	23.2	26.6	30.6	34.9	39.4	44.1	<0.001	5,204,433
Non-manual worker	50.8%	59.6%	66.6%	63.8%	54.5%	41.9%	<0.001	3,685,070
Completed secondary school	15.9%	23.7%	34.0%	35.7%	31.3%	24.1%	<0.001	3,766,998
Alive when offspring 16	97.7%	97.9%	97.8%	96.9%	94.8%	91.3%	<0.001	3,542,270
Alive when offspring 40	84.4%	82.7%	77.8%	66.5%	50.7%	33.6%	<0.001	2,147,003
***Offspring***								
Non-manual worker	35.7%	42.6%	48.0%	51.1%	50.4%	47.2%	<0.001	3,048,381
Completed secondary school	41.2%	52.1%	60.7%	59.7%	53.4%	44.8%	<0.001	5,159,950
Male	51.0%	51.0%	51.2%	51.0%	51.1%	50.5%	<0.001	5,204,433
First-born	91.1%	64.0%	42.4%	28.5%	24.7%	26.1%	<0.001	5,204,433
Smoker at age 18^a^	67.5%	60.8%	57.5%	54.8%	55.7%	56.5%	<0.001	45,006
Left-handed^a^	8.6%	8.6%	8.4%	8.2%	8.4%	8.3%	<0.001	1,304,248
Date of birth	1962	1962	1963	1961	1958	1954	<0.001	5,204,433
Birth weight (kg)	3.4	3.5	3.5	3.5	3.5	3.5	<0.001	1,377,985
Birth length (cm)	50.1	50.3	50.5	50.5	50.5	50.3	<0.001	1,374,771
Height at age 18 (cm)^a^	178.3	179.0	179.6	179.8	179.6	179.2	<0.001	1,592,625
BMI at age 18 (kg m^−2^)^a^	22.0	21.9	21.9	21.9	21.8	21.7	<0.001	1,592,271
SBP at age 18 (mm Hg)^a^	127.7	128.3	128.7	128.9	128.9	128.9	<0.001	1,517,662
DBP at age 18 (mm Hg)^a^	67.0	67.2	67.5	68.1	68.4	68.7	<0.001	1,517,457
Intelligence at age 18^a^	4.6	4.9	5.3	5.3	5.3	5.1	<0.001	1,648,011
Non-cognitive ability at age 18^a^	5.0	5.2	5.4	5.3	5.1	4.9	<0.001	1,079,880

^a^Measured in male offspring only, at military service medicals 1969-1970 (smoking) or 1969-2001 (other measurements).

**Table 2:** Description of the study population by classes of paternal age.

Person, variable	Father's age at offspring birth							P	N
	≤19	20–24	25–29	30–34	35–39	40–44	≥45		
*Mothers*									
Age at offspring birth (years)	19.2	21.9	25.4	29.0	32.4	35.2	37.3	<0.001	5,204,433
Non-manual worker	38.1%	40.0%	45.6%	43.2%	34.2%	25.6%	21.4%	<0.001	4,590,785
Completed secondary school	9.9%	12.9%	20.2%	22.5%	20.0%	16.3%	15.7%	<0.001	3,921,575
Alive when offspring 16	99.1%	99.3%	99.2%	98.9%	98.5%	97.8%	97.1%	<0.001	3,542,302
Alive when offspring 40	93.5%	92.9%	91.6%	88.5%	83.6%	77.4%	70.2%	<0.001	2,147,035
*Fathers*									
Age at offspring birth (years)	19.0	22.9	27.5	32.2	37.1	42.0	49.0	<0.001	5,204,433
Non-manual worker	56.2%	61.4%	66.5%	64.7%	56.5%	47.7%	42.5%	<0.001	3,685,070
Completed secondary school	19.1%	22.9%	31.1%	33.5%	30.7%	25.7%	24.5%	<0.001	3,766,998
Alive when offspring 16	97.9%	98.2%	98.3%	97.7%	96.4%	93.8%	86.9%	<0.001	3,542,270
Alive when offspring 40	87.6%	86.7%	83.3%	75.1%	61.3%	43.1%	19.5%	<0.001	2,147,003
*Offspring*									
Non-manual worker	35.1%	40.1%	45.6%	49.9%	50.5%	48.3%	44.5%	<0.001	3,048,381
Completed secondary school	40.4%	49.5%	58.7%	60.0%	55.5%	49.7%	45.0%	<0.001	5,159,950
Male	51.3%	51.0%	51.2%	51.1%	51.0%	51.0%	50.7%	<0.001	5,204,433
First-born	93.6%	74.5%	53.1%	36.5%	28.8%	27.1%	28.9%	<0.001	5,204,433
Smoker at age 18^a^	71.6%	63.8%	58.5%	56.4%	56.7%	54.9%	56.0%	<0.001	45,006
Left-handed^a^	8.3%	8.5%	8.4%	8.4%	8.3%	8.4%	8.4%	0.368	1,304,248
Date of birth	1963	1963	1963	1962	1959	1956	1954.8	<0.001	5,204,433
Birth weight (kg)	3.4	3.4	3.5	3.5	3.5	3.5	3.5	<0.001	1,377,985
Birth length (cm)	50.0	50.2	50.4	50.5	50.5	50.4	50.4	<0.001	1,374,771
Height at age 18 (cm)^a^	178.3	178.9	179.4	179.6	179.6	179.4	179.1	<0.001	1,592,625
BMI at age 18 (kg m^-2^)^a^	22.0	21.9	21.9	21.9	21.9	21.8	21.8	<0.001	1,592,271
SBP at age 18 (mm Hg)^a^	127.5	128.0	128.5	128.7	128.9	128.9	128.9	<0.001	1,517,662
DBP at age 18 (mm Hg)^a^	66.9	67.0	67.3	67.9	68.2	68.4	68.4	<0.001	1,517,457
Intelligence at age 18^a^	4.6	4.8	5.2	5.3	5.3	5.2	5.1	<0.001	1,648,011
Non-cognitive ability at age 18^a^	5.0	5.2	5.4	5.3	5.2	5.0	4.9	<0.001	1,079,880

Analyses were restricted to those offspring included in the primary analyses of cause-specific mortality. Within each class of paternal age, continuous variables were summarised as means, binary variables as percentages. P values tested for heterogeneity in each variable between the classes of paternal age. They came from unadjusted linear or logistic regressions with robust standard errors clustered by the father’s identity. Intelligence and non-cognitive ability were each recorded on a scale of 1–9. ^a^Measured in male offspring only, at military service medicals 1969–1970 (smoking) or 1969–2001 (other measurements).

**Figure 1:**
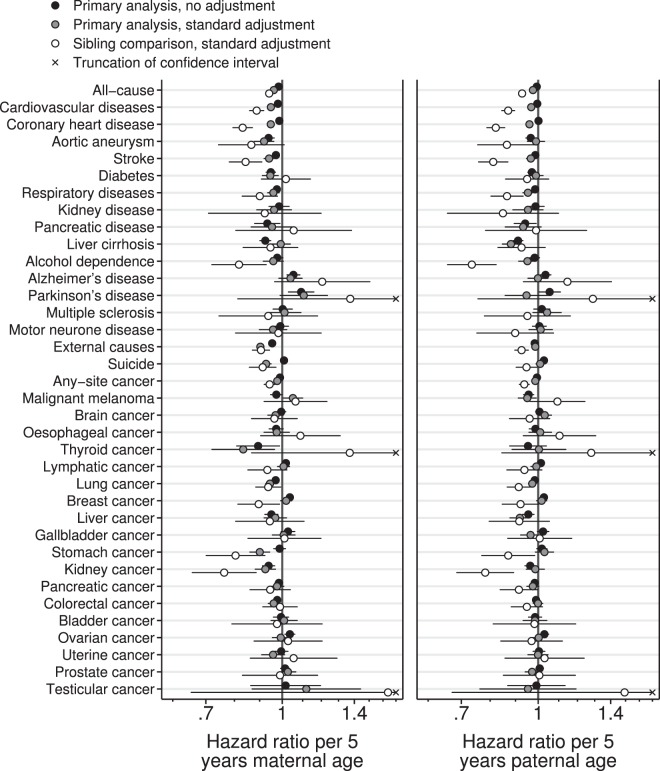
Log-linear hazard ratios for offspring mortality against maternal or paternal age. 95% confidence intervals are truncated for clarity where indicated with crosses. Primary analyses used Cox proportional hazards regression with robust standard errors clustered by parental identity. Standard adjustment comprised offspring sex, DOB and birth order, maternal and paternal occupational and educational SEP and the other parent’s age at the time of the offspring’s birth. Sibling-comparison analyses used Cox regression stratified by the identity of the parent in question. They were conducted on the restricted dataset and adjusted for offspring sex and birth order (adjustment for parental SEP was unnecessary and adjustment for offspring DOB and the other parent’s age were impossible). Plotted values, sample sizes and the number of deaths from each cause may be seen in Tables 3–4 and Supplementary Tables S2–S5.

**Figure 2:**
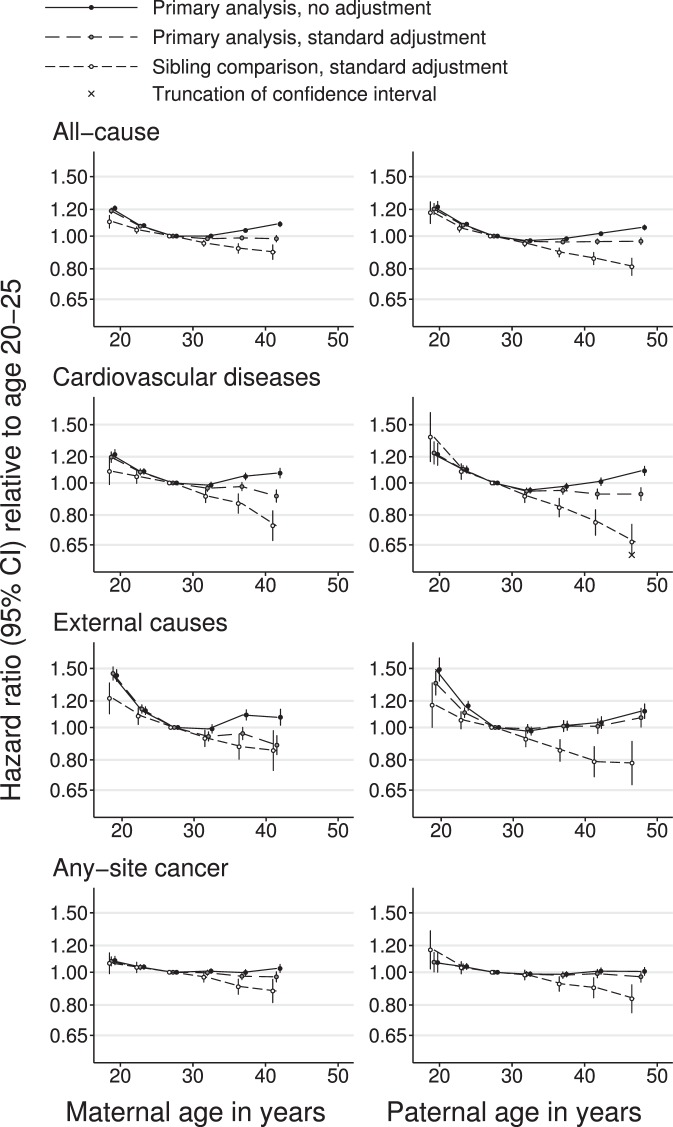
Hazard ratios for offspring mortality against classes of maternal or paternal age. Parental age in years was put into classes of <20, 20–24, 25–29 (reference), 30–34, 35–39, 40–44 and ≥45 and each class was plotted at its median. Points, but not lines, are transposed horizontally by +/−0.5 years for clarity. The two oldest classes were combined for maternal age. Error bars are 95% confidence intervals. Primary analysis associations are shown with no adjustment and with adjustment for offspring sex, DOB, maternal and paternal occupational and educational SEP, offspring birth order, and the other parent’s parental age. Sibling comparison analyses are shown with adjustment for offspring sex and birth order. Selected causes of death; full results are shown in Supplementary Fig. S2.

Unadjusted linear primary analyses found that greater maternal or paternal age were associated with slightly reduced offspring mortality from most common causes of death (Fig. 1). Adjustment for parental SEP strengthened these negative associations slightly. Associations with paternal, but not maternal, age were somewhat attenuated by further adjustment for the other parent’s age (Supplementary Tables S2 and S3). Analyses by categories of parental age suggested that these negative associations levelled off or reversed for parents older than 30, especially in the unadjusted analyses (Fig. 2, Supplementary Fig. S2). In opposition to this general pattern, the daughters of older parents appeared to be at increased risk of dying from breast cancer. Mortality from Alzheimer’s and Parkinson’s diseases were also increased in the offspring of older mothers but results for older fathers were unclear.

For the sibling comparison analyses a restricted dataset was necessary, containing only those offspring who had a sibling with whom they were discordant for both the exposure and the outcome. This substantially reduced the sample size, particularly for rarer causes of death (Tables 3 and 4, Supplementary Tables S4 and S5). Sibling comparison analyses of common causes of death suggested much stronger reductions in mortality with increased parental age than the primary analyses did. The positive associations suggested by the primary analyses between parental age and breast cancer mortality were reversed in the sibling comparison analyses. The positive associations with mortality from Alzheimer’s and Parkinson’s diseases were an exception, becoming more positive in the sibling comparison analyses (albeit with wide confidence intervals). Hazard ratios from sibling comparison analyses were generally wide, meaning that they could not be distinguished from the null for many rarer causes of death (Fig. 1). When sibling comparison analyses were applied to categories of parental age, the associations with common causes of death were close to linear with mortality continuing to decline for the oldest parents (Fig. 2).

**Table 3:** Analyses decomposing the difference between the primary and sibling-comparison analyses of offspring mortality and maternal age, for outcomes causing >10,000 deaths in the main dataset.

Outcome	Deaths (main data)	Deaths (restricted data)	N (restricted data)	Hazard ratio per five years of mother’s age at offspring’s birth (or per five years of offspring’s DOB for secular trend)				
				Primary analysis (main data)	Secular trend (main data)	Primary analysis plus mediation (main data)	Primary analysis (restricted data)	Sibling-comparison analysis (restricted data)
All-cause	293,470	190,795	449,224	0.96 (0.96, 0.97)	0.94 (0.94, 0.94)	0.91 (0.90, 0.91)	1.03 (1.03, 1.04)	0.94 (0.93, 0.96)
Cardiovascular disease	76,352	45,734	118,683	0.95 (0.94, 0.96)	0.88 (0.87, 0.88)	0.83 (0.82, 0.85)	1.03 (1.02, 1.03)	0.89 (0.86, 0.92)
Coronary heart disease	41,853	25,214	67,767	0.95 (0.93, 0.96)	0.84 (0.83, 0.85)	0.80 (0.79, 0.82)	1.02 (1.01, 1.03)	0.83 (0.79, 0.87)
Stroke	14,692	8,391	22,973	0.94 (0.92, 0.96)	0.86 (0.84, 0.87)	0.81 (0.79, 0.83)	1.03 (1.02, 1.05)	0.84 (0.78, 0.91)
Respiratory diseases	12,187	6,880	18,537	0.96 (0.93, 0.99)	0.92 (0.90, 0.95)	0.89 (0.86, 0.92)	1.04 (1.03, 1.06)	0.90 (0.83, 0.98)
External causes	38,658	27,050	73,054	0.90 (0.89, 0.92)	0.98 (0.97, 0.98)	0.88 (0.86, 0.89)	1.07 (1.06, 1.08)	0.91 (0.87, 0.94)
Suicide	15,565	11,106	30,276	0.93 (0.91, 0.95)	0.97 (0.95, 0.98)	0.90 (0.87, 0.92)	1.07 (1.05, 1.08)	0.91 (0.86, 0.97)
Any cancer	113,597	71,881	184,833	0.98 (0.97, 0.99)	0.93 (0.92, 0.94)	0.91 (0.90, 0.92)	1.04 (1.04, 1.05)	0.94 (0.92, 0.97)
Lung cancer	20,752	12,998	35,452	0.94 (0.93, 0.96)	0.93 (0.91, 0.95)	0.88 (0.86, 0.91)	1.04 (1.03, 1.06)	0.94 (0.88, 0.99)
Breast cancer	10,887	4,587	10,862	1.02 (0.99, 1.05)	0.91 (0.89, 0.93)	0.93 (0.90, 0.96)	1.00 (0.98, 1.02)	0.90 (0.81, 0.99)
Colorectal cancer	12,325	7,357	19,886	0.96 (0.94, 0.99)	0.97 (0.95, 0.99)	0.94 (0.91, 0.97)	1.07 (1.05, 1.08)	0.99 (0.91, 1.07)

Results for all causes of death may be seen in Supplementary Table S4. Primary analyses used Cox proportional hazards regression of 2,658,132 male and 2,546,301 female offspring. Age was the time axis and robust standard errors were clustered by maternal identity. Adjustment set (e) (offspring sex and date of birth (DOB), maternal and paternal occupational and educational SEP, offspring birth order, and paternal age) was used. The secular trend per five years of offspring DOB was assessed using a similar model but without maternal age. The primary analysis was repeated with adjustment for maternal, not offspring, DOB to account for confounding, but not mediation, by secular trends. To examine whether the restricted dataset used for sibling-comparison analyses was representative of the main dataset, the primary analysis was repeated on this subset. Finally, the sibling-comparison analysis used Cox regression stratified by maternal identity and was restricted to offspring in families with discordant outcomes (maximum N = 449,224 for all-cause mortality). All family-level confounding was intrinsically adjusted for and adjustment for offspring DOB or paternal age were not possible. Explicit adjustment was therefore limited to offspring sex and birth order.

**Table 4:** Analyses decomposing the difference between the primary and sibling-comparison analyses of offspring mortality and paternal age, for outcomes causing >10,000 deaths in the main dataset.

Outcome	Deaths (main data)	Deaths (restricted data)	N (restricted data)	Hazard ratio per five years of father’s age at offspring’s birth (or per five years of offspring’s DOB for secular trend)				
				Primary analysis (main data)	Secular trend (main data)	Primary analysis plus mediation (main data)	Primary analysis (restricted data)	Sibling-comparison analysis (restricted data)
All-cause	293,470	190,579	451,278	0.98 (0.97, 0.98)	0.94 (0.94, 0.94)	0.92 (0.91, 0.93)	0.98 (0.98, 0.98)	0.93 (0.92, 0.94)
Cardiovascular disease	76,352	45,690	118,949	0.97 (0.96, 0.98)	0.88 (0.87, 0.88)	0.85 (0.84, 0.86)	0.98 (0.97, 0.99)	0.87 (0.84, 0.90)
Coronary heart disease	41,853	25,182	67,910	0.96 (0.95, 0.97)	0.84 (0.83, 0.85)	0.81 (0.80, 0.83)	0.98 (0.97, 0.98)	0.82 (0.79, 0.86)
Stroke	14,692	8,356	22,951	0.97 (0.95, 0.99)	0.86 (0.84, 0.88)	0.83 (0.81, 0.86)	0.98 (0.96, 0.99)	0.81 (0.76, 0.87)
Respiratory diseases	12,187	6,863	18,556	0.95 (0.93, 0.98)	0.92 (0.90, 0.94)	0.88 (0.86, 0.91)	0.99 (0.98, 1.01)	0.87 (0.80, 0.93)
External causes	38,658	26,951	73,616	0.99 (0.97, 1.00)	0.97 (0.96, 0.98)	0.96 (0.95, 0.98)	0.97 (0.96, 0.98)	0.93 (0.90, 0.96)
Suicide	15,565	11,062	30,489	1.01 (0.99, 1.03)	0.97 (0.95, 0.98)	0.98 (0.95, 1.00)	0.98 (0.97, 0.99)	0.95 (0.90, 0.99)
Any cancer	113,597	71,927	185,758	0.99 (0.98, 0.99)	0.93 (0.92, 0.94)	0.92 (0.91, 0.93)	0.98 (0.98, 0.99)	0.94 (0.92, 0.96)
Lung cancer	20,752	12,939	35,479	0.97 (0.96, 0.99)	0.93 (0.91, 0.95)	0.91 (0.89, 0.93)	0.98 (0.97, 0.99)	0.91 (0.87, 0.96)
Breast cancer	10,887	4,630	10,967	1.02 (0.99, 1.04)	0.91 (0.89, 0.93)	0.93 (0.90, 0.95)	0.97 (0.95, 0.99)	0.92 (0.85, 1.00)
Colorectal cancer	12,325	7,354	20,003	1.00 (0.98, 1.02)	0.97 (0.95, 1.00)	0.97 (0.94, 1.00)	0.98 (0.97, 0.99)	0.95 (0.88, 1.02)

Results for all causes of death may be seen in Supplementary Table S5. Primary analyses used Cox proportional hazards regression of 2,658,132 male and 2,546,301 female offspring. Age was the time axis and robust standard errors were clustered by paternal identity. Adjustment set (e) (offspring sex and date of birth (DOB), maternal and paternal occupational and educational SEP, offspring birth order, and maternal age) was used. The secular trend per five years of offspring DOB was assessed using a similar model but without paternal age. The primary analysis was repeated with adjustment for paternal, not offspring, DOB to account for confounding, but not mediation, by secular trends. To examine whether the restricted dataset used for sibling-comparison analyses was representative of the main dataset, the primary analysis was repeated on this subset. Finally, the sibling-comparison analysis used Cox regression stratified by paternal identity and was restricted to offspring in families with discordant outcomes (maximum N = 451,376 for all-cause mortality). All family-level confounding was intrinsically adjusted for and adjustment for offspring DOB or maternal age were not possible. Explicit adjustment was therefore limited to offspring sex and birth order.

There were negative secular trends (i.e. declining mortality) over the period of the study for all common causes of death (Tables 3 and 4) and for several of the rarer ones (Supplementary Tables S4 and S5). Among the rarer causes of death, however, trends for Alzheimer’s disease, Parkinson’s disease, motor neurone disease and malignant melanoma were positive and those for some others could not be distinguished from the null. When the linear primary analyses were adjusted for parental DOB instead of offspring DOB (thus including an effect mediated by secular trends in the association between parental age and mortality), the hazard ratios per five years of parental age for most common causes of death were amplified by a factor corresponding approximately to the observed secular trend. When the primary analyses were repeated on the restricted dataset used in the sibling comparison analyses, associations with maternal age reversed, becoming weakly positive for most causes of death. In contrast, those with paternal age remained weakly negative (Tables 3 and 4, Supplementary Tables S4 and S5).

Tests for most specific causes of death did not indicate departure from the proportional hazards assumption, but hazard ratios for all-cause mortality did decline slightly with the offspring’s age, particularly when the exposure was paternal age (Supplementary Tables S18 and S19). When associations between mortality and parental age were analysed separately for male and female offspring, there were no clear qualitative differences (Supplementary Tables S20 and S21). Primary analyses suggested that reduced mortality from cardiovascular diseases was slightly more associated with higher paternal age in sons and with higher maternal age in daughters. In the sibling-comparison analysis, however, the apparent cardiovascular benefits of both higher paternal and higher maternal age were greater for sons than for daughters. There was also a suggestion that higher maternal age was more protective against external causes mortality in daughters than in sons. The sex-specific sibling-comparison analyses suffered from additional power loss and potential selection bias because of the necessary restriction to outcome-discordant same-sex sibling groups. There was some evidence, particularly for paternal age, that the lower mortality from CVD and CHD among the offspring of older parents was most apparent when the offspring were older and absent or reversed when the offspring were under 40, although the low number of deaths made estimates for younger offspring imprecise. A similar but weaker pattern for all-cause mortality was probably driven by mortality from CVD.

## Discussion

### Mechanisms associating parental age and offspring survival

**Figure 3:**
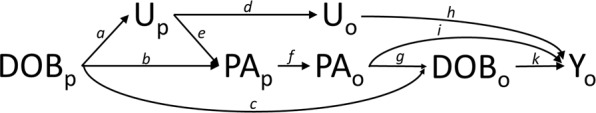
Directed acyclic graph illustrating pathways between dates of birth (DOB), parental age (PA), unmeasured confounders (U) and mortality outcomes (Y). Subscripts p and o represent variables at the parental and offspring level, respectively. PA_p_ represents a parent’s tendency to earlier or later parenthood, not the age of the parent’s parents. Note that DOB_o_ is completely determined by DOB_p_ and PA_o_; effects *c* and *g* both have known linear coefficients of 1. A primary analysis of the effect of PA_o_ on Y_o_ comprises a direct effect (*i*), an effect mediated by DOB_o_ (*gk*) and associations due to family-level confounding (*fedh*, *fbadh*). When the primary analysis is adjusted for DOB_o_ the mediated effect is blocked but a perfect negative association between PA_o_ and DOB_p_ is induced, changing the nature of the family-level confounding *[gc]adh*. If the primary analysis is adjusted for DOB_p_ the direct (*i*) and indirect (*gk*) causal effects of PA_o_ on Y_o_ apply but the pathways for family-level confounding (*fedh*) are reduced. In a sibling-comparison analysis all family-level confounding is blocked and individual-level confounding is unlikely given that the exposure is determined before birth. Adjustment for DOB_o_ is impossible because it is perfectly positively associated within families with PA_o_. The estimates from the sibling comparison analysis thus comprise the direct (*i*) and indirect (*gk*) causal effects of PA_o_ on Y_o_.

Figure 3 shows some of the pathways by which parental age may be associated with survival in the offspring. Parental age may have “direct” causal effects *i* on offspring survival (i.e. not mediated by any variables shown in Fig. 3). This is often the pathway we wish to isolate, but while all the analytical methods employed here include it, none does so in isolation from other pathways. No method that we are aware of does so. The diverse direct effects of parental age have already been listed above and include various genetic, physiological, behavioural and socio-economic changes as individual parents age. The total causal effect of parental age also includes an indirect effect *gk* mediated by the offspring’s DOB. In a society with positive secular trends in survival, parents who delay childbirth will increase their offspring’s survival simply by bringing them into a modern world more conducive to longevity^7^. This mechanism is included in all the analyses presented here, except those which have been adjusted for offspring DOB (i.e. the adjusted primary analysis in Tables 3 and 4 and Fig. 1). We often wish to exclude this process from estimates of parental age effects because it is wholly dependent on secular trends in the society under consideration, limiting the generalisability of our results. Most parts of the world have seen consistent long-term improvements in longevity^9,10^, but there is some evidence suggesting that these improvements may be coming to an end or reversing in some developed countries^11–13^.

In addition to these causal effects, the analyses presented here all include some confounded pathways which we would usually like to eliminate from our estimates. The unadjusted primary analysis (Fig. 1, Supplementary Tables S2 and S3) include confounding *fedh* by family-level variables, including family level secular trends (*fbadh*). For example, higher SEP in the parents is often associated with delayed childbirth and, via higher SEP in the offspring, with improved offspring survival. Note that this confounding by lifelong parental SEP is distinct from the mediation of causal parental age effects on offspring survival by the increasing SEP of older parents. Similarly, family-level confounding may be due to lifelong genetic variants affecting age at parenthood in the parents and survival in the offspring, but age-related genetic changes in parental germlines may mediate causal effects of parental age. Adjustment for family-level covariates in the adjusted primary analyses (Fig. 1, Tables 3 and 4) eliminates this confounded pathway only insofar as the measured covariates represent all relevant family-level confounders U_p_ (Fig. 3). Furthermore, adjustment for offspring DOB in the adjusted primary analyses blocks the effect *gk* mediated by offspring DOB, but because offspring DOB is fully determined by parental DOB and parental age, conditioning on this collider induces a perfect negative association between them. This strengthens the confounding via family-level secular trends *[gc]adh*. When primary analyses are adjusted for parental DOB instead of offspring DOB (Tables 3 and 4), there is no confounding via family-level secular trends, but the family-level confounding *fedh* via other covariates remains, and the causal effect of parental age includes the effect mediated by offspring DOB. Finally, in a sibling-comparison analysis (Fig. 1, Tables 3 and 4), all family-level confounding is automatically accounted for. This is particularly attractive in an analysis of parental age, because the determination of the exposure before birth means that substantial individual-level confounding (which can cause bias in a sibling-comparison analysis^14^) is unlikely. However, there are two potentially important drawbacks to this method. First, offspring DOB is perfectly associated with parental age within families and so cannot easily be adjusted for^8,15,16^. This means that the estimates from sibling comparison analyses include the mediated effect *gk* (Fig. 3). A similar argument applies to adjustment for the other parent’s age (except for the usually small proportion of the population who have half-siblings, if families are defined by only one parent’s identity). It has been suggested^17^ that these issues may be remedied in the presence of nonlinearity; the power and feasibility of such an approach remains to be demonstrated. Second, a sibling comparison analysis is necessarily restricted to individuals from families discordant for the exposure and outcome. If multiple births are excluded and parental age is treated as a continuous exposure, parental age is necessarily discordant within families. However, in a survival analysis with extensive right-censoring, within-family concordance in the outcome leads to a heavily selected sample which may not be representative of the target population. In the present study, use of the restricted dataset to analyse all-cause mortality and maternal age reduced the sample size from 5,204,433 to 449,224, increased the mortality rate from 3.0 to 24.1 per 1000 person-years, changed the mean DOB from 1961.2 to 1947.6 and increased the mean number of siblings from 1.6 to 2.3. This dramatic change in mean DOB may explain why hazard ratios for maternal age became positive while those for paternal age remained unchanged, when the primary analysis was repeated in the restricted dataset. Separate primary analyses for people born before and after 1970 (Supplementary Tables S8 and S9) suggest that hazard ratios were becoming more negative with time for maternal age but more positive for paternal age.

### Parental age and survival

The data used in this study were collected over a period of fast-declining age-specific mortality. It is therefore surprising that adjustment for secular trends made no appreciable difference to the hazard ratios for parental age in primary analyses (compare adjustment sets a and b in Supplementary Tables S2 and S3), suggesting that effects of parental age mediated by secular trends are minimal. However, the interpretation of models with and without adjustment for offspring DOB is rather complex. As well as individual-level mediation by secular trends, there may also be family-level confounding. Over the period of this study, average parental age fell rapidly until about 1970 before rising again equally rapidly^8^. When primary analyses in these two periods were analysed separately (Supplementary Tables S8 and S9), the apparent benefits of having an older mother increased after 1970, while those of having an older father decreased. It should be noted that adjusting for offspring DOB induces a strong negative association between parental age and the parent’s DOB, making the analysis more vulnerable to confounding by family-level secular trends. Mediation by secular trends in a primary analysis may be less important than confounding by family-level trends if the data collection period is relatively much longer than the typical interval between a mother’s first and last offspring. When the primary analysis was adjusted for parent, not offspring, DOB it avoided family-level confounding by secular trends (but did not avoid other family-level confounding) while reintroducing the mediated effect of parental age. This amplified the negative associations between parental age and mortality (Tables 3 and 4), but we are unable to say how much of this amplification was due to the additional mediation, versus the removal of family-level confounding by secular trends. Linear sibling comparison analyses suggested that the offspring of older mothers and fathers benefitted from increased survival, after removal of all family-level confounding. However, these associations included mediation by secular trends (likely to be stronger within families) and could not adjust for effects mediated or confounded by the other parent’s age. Furthermore, repetition of the primary analyses on the restricted dataset used for the sibling comparison analyses suggested a strong selection bias towards a benefit of having younger mothers, but no overall bias with respect to paternal age. Matters are complicated still further by the two-variable analyses (Supplementary Tables S6 and S7) which suggest a negative association between maternal or paternal age and offspring mortality due to family-level confounding and a positive association at the individual level once this confounding is accounted for. However, this technique is vulnerable to induced confounding at the family level due to conditioning on two colliders (offspring DOB and family-level parental age)^8^.

The most relevant study of all-cause mortality for the present study was published by Barclay & Myrskylä (B&M)^7^ using most of the same data as the present study. Their primary analyses found broadly similar results to ours; a weakly negative or U-shaped association between maternal or paternal age and mortality became more strongly negative when adjusted for SEP. It is however concerning that there were considerable differences between their sibling comparison results and ours, which may reflect the sensitivity of sibling comparison analyses of parental age to small details of adjustment and selection. Their minimally-adjusted sibling comparison analysis of paternal age produced a similar association to ours; a near-linear negative association with mortality. For maternal age, however, our sibling comparison analysis gave a similar result to paternal age whereas B&M found a shallow U-shape with a greater increase in mortality among offspring of the oldest mothers. Since the same data resource was used in both analyses, these differences must be due to one or more of the small differences in selection and adjustment. We applied six of these minor changes to our analysis individually (see Supplementary Note in Supplementary Material). The exclusion of people born after December 1960 (rather than December 1987 in our main analyses) diminished the apparent disadvantage among offspring of young mothers. These more recent offspring would only have been followed up in early adulthood; an age at which the negative associations between all-cause mortality and parental age were slightly less pronounced (Supplementary Tables S18 and S19). The use of follow-up from January 1990 (rather than July 1991 in our main analyses) diminished the apparent advantage among offspring of older mothers. We chose to exclude follow-up prior to July 1991 because of differential missingness up to this time. When we repeated the sibling comparison analysis including the potentially biased follow-up from 1961 to June 1991 (Supplementary Tables S16 and S17), the associations between parental age and all-cause mortality switched from negative to positive. In our sibling comparison analyses, we did not adjust for the other parent’s age due to concerns over co-linearity. When we did this to replicate the analysis of B&M, the hazard ratios moved towards those of B&M. However, it was only when all six changes were made to our analysis that our results for maternal age approximated those of B&M. Furthermore, our sibling-comparison results for paternal age resembled those of B&M when any one (or none) of the six changes was made but deviated from them somewhat when all six were made simultaneously. A thorough theoretical and/or simulation investigation of the properties of sibling comparison methods, when applied to analyses of parental age, should be a priority in this field.

Negative effects of having older parents may be mediated through the risk of the offspring losing their parents at a relatively early age. Previous studies of all-cause adult mortality in the USA^5^ and Finland^18^ found that adjustment for parental survival until the offspring’s 40^th^ or 35^th^ birthday, respectively, attenuated negative associations between advanced parental age and offspring survival. We found that negative linear associations between parental age and offspring mortality from primary analyses became slightly stronger with adjustment for parental survival to the offspring’s 35^th^ birthday, consistent in direction with these previous results but insufficient in magnitude to be the major mechanism by which parental age affects offspring survival.

Most other studies of parental age and offspring adult mortality have been heavily constrained by data availability; either following people up to early middle age or relying on relatively small samples with few covariates from historical data. One study of Finns^19^ followed up to age 39, and another of Danes^20^ followed up to age 40, both found evidence of elevated all-cause mortality in the offspring of older fathers and younger mothers. Mortality in this age group is dominated by external causes, and so these all-cause results are not comparable with ours. Three other studies^21–23^ have followed people up to very old age or extinction and found no association between parental age and survival. However, these studies all had relatively low sample sizes and limited power. They also lacked covariate data, and it should be noted that in our primary analyses, the crude associations were close to the null but became more strongly negative when adjusted for SEP.

### Parental age and breast cancer

We are only aware of one study^24,25^ of parental age and breast cancer mortality, but there have been several studies of parental age and breast cancer incidence. Most have found a small increase in breast cancer with maternal age, but weaker associations with paternal age that attenuate when adjusted for maternal age^24,26–30^. These results are usually ascribed to age-related changes in the intra-uterine hormonal environment. Two studies^31,32^ have found the reverse; that breast cancer was more closely associated with paternal than maternal age. One of these studies^32^ also noted that telomere length increased with paternal age and suggested that this could have increased the risk of breast cancer in the daughters of older fathers. In the primary analyses of the present study, we found weak positive associations between breast cancer mortality and parental age that were similar for paternal and maternal age. They did not change greatly with adjustment and could not confidently be distinguished from the null. This is consistent with an earlier study^33^ of breast cancer incidence using a shorter follow-up (to 1994) of the same data source. The sibling comparison analyses of paternal and maternal age both gave a strong negative association which should probably be ascribed to the particularly strong negative secular trend in this outcome.

### Parental age and external causes mortality

External causes (accidents, violence and suicide) are a leading cause of death in younger and middle-aged adults and are therefore one of the better-studied outcomes in relation to parental age, given that most cohorts with parental age data have not yet been followed up to old age. With follow-up usually to the age of 30–40, several studies have found an elevated risk of suicide and/or accidental death in the offspring of younger mothers^19,20,34–37^. In two of these six studies, there was also an elevated risk in those with older mothers such that the overall association between suicide and maternal age was U-shaped, although this was attenuated with adjustment for SEP. Only three of these studies also analysed paternal age, with two^19,20^ finding increased suicide and accidental mortality in the offspring of older fathers but also to a lesser extent in the offspring of young fathers. The third^36^, a case-control study, found increased suicide mortality in the offspring of younger, but not older, fathers. The present results are consistent with the majority of studies of maternal age in emphasising the increased risk in offspring of younger mothers. The results for paternal age presented here pick up elements of the contrasting previous results for paternal age, with elevated mortality at both extremes of paternal age resulting in a hazard ratio from the linear analysis that was close to the null. Interestingly, a study of bipolar disorder in the Swedish population found a strong positive association with maternal and paternal age^38^. Since bipolar disorder is a strong risk factor for suicide^39^, this runs contrary to the negative or U-shaped associations for suicide. Only one previous study^34^ to our knowledge fitted sibling comparison models, and only for maternal age and offspring suicide mortality. These were imprecise and close to the null with minimal adjustment, but adjustment for birth order gave a strong negative association between maternal age and suicide risk. This change was ascribed to a strong increase in suicide rates among the later offspring of a family. We also found a negative association in our sibling comparison studies (also adjusted for birth order).

Parental age may affect the risk of suicide in offspring through the childhood environment or be associated with it through residual socioeconomic confounding, although the association from sibling comparison analyses argues against the latter. Mediation through secular trends is unlikely, given the very minor secular trends identified in the present study. Associations of parental age with psychiatric disorders have been relatively well studied^40,41^, and might mediate effects on mortality, particularly from external causes. However, a further possibility is confounding by an inherited genetic tendency to both mental illness (and thus external causes mortality) and early parenthood^42^. There is increasing evidence suggesting that this may explain much of the association between parental age and offspring schizophrenia^42–44^ although since lifelong parental genetics is a family-level confounder, the mechanism would not apply in a sibling comparison analysis.

### Parental age and Alzheimer's and Parkinson’s diseases

In contrast to most causes of death, we found evidence suggesting that mortality from Alzheimer’s and Parkinson’s diseases increased among the offspring of older parents. These are both diseases which usually become manifest in old age and this result could be interpreted as the result of an inherited tendency to a “slow” life history. However, they are also both among the few causes of death displaying positive secular trends over the course of the study (Supplementary Tables S4 and S5). The positive associations between parental age and mortality from Alzheimer’s and Parkinson’s diseases were most apparent in those analyses which did not adjust for secular trends in the offspring, particularly the sibling comparison analysis, and mediation by secular trends may largely explain these results. Confidence intervals were wide, but of the other causes of death with positive secular trends (motor neurone disease, malignant melanoma, oesophageal cancer, thryoid cancer, liver cancer and uterine cancer) all but motor neurone disease and liver cancer also showed positive associations with parental age in the sibling comparison analysis. Given that an analysis of parental age and mortality from Alzheimer’s or Parkinson’s disease requires a cohort followed up to old age, with data on their parents age, it is unsurprising that we are not aware of any previous cohort studies of this association. Even in the present study (maximum age 80.75 years), mortality from these conditions is probably under-represented and an unusually high proportion of these deaths might be considered early-onset Alzheimer’s or Parkinson’s disease. A meta-analysis^45^ of four case-control studies found suggestive evidence for increased Alzheimer’s disease risk among those with mothers over 40 years old, and perhaps also among those with teenage mothers. Diseases of old age affecting the quality of life as well as potentially causing mortality are likely to be of increasing importance and more robust studies of this association would be useful if suitable data can be identified.

### Parental age and other cancer sites

A study by Hemminki & Kyyrönen^33^ used the same data source as the present study but examined site-specific cancer incidence, rather than mortality, with follow up only to 1994 (maximum age 53). They found melanoma incidence to be positively associated with maternal age and negatively associated with paternal age; a result we repeated for melanoma mortality. The positive association with maternal age was only apparent after adjustment for birth order and, particularly, paternal age. Positive associations in the sibling comparison analyses were probably driven by positive secular trends of similar magnitude. Our analysis of brain cancer mortality also replicated their suggestive findings for nervous system cancer incidence; a weak negative association with maternal age and a weak positive association with paternal age. Hemminki & Kyyrönen found a positive association of leukemia incidence with maternal, but not paternal age. Another study^46^, of non-Hodgkin lymphoma incidence, found the opposite result; a positive association with paternal but not maternal age. Our analysis of lymphatic cancer mortality combined these two cancers and our null findings for both maternal and paternal age may have been due to this heterogeneity in the outcome, although the association with maternal age was positive before adjustment for sex, DOB and SEP.

Mortality from prostate cancer was not strongly associated with parental age in primary analyses, although there was a suggestion of a negative association with paternal age after adjustment for SEP, birth order and maternal age. Sibling comparison analyses were low-powered but did not suggest a strong association. A negative association with paternal age contradicts a study of prostate cancer incidence^47^, adjusted for SEP and maternal age but not birth order, which found a suggestive increase in the sons of older fathers. Differences between studies of incidence and mortality is unsurprising, given the low case-fatality rate of prostate cancer and the fact that both estimates were of low magnitude and, in the case of the former study, low precision.

### Strengths and weaknesses

The study benefits from a very large, population-based dataset following some participants into old age, giving it the power to examine some rarer causes of death with reasonable precision. Nonetheless, only the earlier cohorts within the data were followed up into old age (maximum age 80.75), which limits the number of deaths (5.6% of participants) and therefore the power in a survival analysis, and also makes age- and cohort-effects difficult to distinguish. We have undertaken numerous sensitivity analyses to try to separate the various pathways by which parental age may be associated with offspring mortality, but the sensitivity of the sibling comparison analyses in particular, to small changes in adjustment and selection means that they should be treated with caution.

Because of non-random missingness, it was not possible to begin follow-up for all participants at age 18. This may have introduced a survivor bias. The decision to exclude childhood follow-up even where it was available was taken deliberately in order to focus on adult survival. The study does therefore presuppose survival to at least age 18.

The sibling comparison analyses emphasise the importance of secular trends in the causal effect of a parent’s decision regarding at what age to have children. The results presented here necessarily represent the secular trends in mortality present over the lifetime of these study participants. Should these trends change, then the within-family associations between parental age and survival for children born in the future could be dramatically different from those reported here.

### Conclusions

For most common causes of death, the offspring of older parents enjoy better survival. Mortality is particularly elevated in the offspring of teenage mothers and fathers, even after adjustment for SEP. This is the opposite pattern to that expected from physiological mechanisms or from the risk of losing a parent early in life. While there may be some residual socioeconomic confounding, adjustment for the available proxies for SEP further improved the apparent advantages of those with older parents rather than attenuating them. It may be that older parents are better able to provide a supportive childhood environment. Our earlier study^8^ of parental age and health-related outcomes in young adult offspring concluded that having older parents was associated at age 18 with physiological disadvantages but social and behavioural advantages. It would appear that as these early consequences of parental age translate into longevity, the physiological disadvantages are more easily mitigated than the social and behavioural ones^6^. Sibling comparison analyses best represent the effect of the family planning choices made by individual parents and emphasise an overwhelming effect of secular trends at this level; the children of older parents have benefitted simply by being born into a society in which financial, social and medical systems have had more time to improve. This somewhat trivial mechanism is completely dependent on trends in the society and time into which a child is born and further methodological research to enable its isolation from other causal effects would be a useful development.

## Methods

### Data preparation

The Swedish multi-generation register 2010 contains approximately 10 million index persons (hereafter, offspring) who were born between 1^st^ January 1932 and 1^st^ December 1987, and registered alive in Sweden in 1961 or later. These were first restricted to the 5,444,697 offspring with information on the DOB of both parents. Dates of birth were provided already rounded to the nearest quarter-year, so this level of precision was used for all dates and ages. Data were linked to national mortality records to give dates and underlying causes of death for all offspring who had died. Causes of death were originally recorded as ICD codes and were converted to more descriptive categories using the translations shown in Supplementary Table S1. Linkage to national records also provided dates of emigration and identified multiple births. Mortality data were available for 1961–2012 but for many offspring who died between 1^st^ January 1969 and 30^th^ June 1991, the identity of their parents was missing and this missingness was not independent of the age of the parents. We therefore analysed follow-up starting from 1^st^ July 1991 or the offspring’s 18^th^ birthday, whichever was latest. Follow-up ended with the death or emigration of the offspring, or on 31^st^ December 2012, whichever was earliest. Furthermore, the data were limited to the 5,300,767 offspring who did not die or emigrate before 1^st^ July 1991 or their 18^th^ birthday, whichever was sooner. Offspring were also excluded if they were from a multiple birth (96,161 exclusions) or if their cause of death was not recorded (173 exclusions), leaving a total of 2,546,301 daughters and 2,658,132 sons.

The Swedish Population and Housing Census provided data on educational and occupational SEP. The maximum attained educational level was recorded in 1970 and 1990 for parents and in 1970 and annually from 1990–2004 for offspring. We took the highest level attained and classified it into seven levels: <9 years; 9–10 years; incomplete secondary education; full secondary education; <3 years tertiary education; >=3 years tertiary education; and missing (0.9% of offspring, 24.7% of mothers and 27.6% of fathers). A binary variable was also created indicating whether or not each person had completed secondary education (i.e. the highest three levels). Occupational status was recorded in 1960, 1970, 1980 and 1990 with only the latter being available for offspring. We took the highest recorded status and classified it into seven levels; high non-manual; intermediate non-manual; low non-manual; skilled manual; unskilled manual; other; and missing. Occupational SEP was set to missing for offspring born after 1972 because many of them would still have been in education in 1990. It was also set to missing for parents born before 1895 because they would mostly have retired by 1960. Occupational status was missing in 30.2% of offspring, 1.2% of mothers and 3.1% of fathers. Others (14.3% of offspring, 23.6% of mothers and 9.6% of fathers) included housewives, part-time workers, farmers and those students or pensioners not set to missing due to their age. A binary variable was also created combining the three non-manual categories.

Linkage to the Medical Birth Register provided data on the birth weight and length of 98.5% of offspring born in 1973 or later. Birth order was coded according to the mother’s previous live births; none, one, two, or more than two. Linkage to conscription medical records provided information on height, BMI, blood pressure, intelligence, non-cognitive ability, handedness and smoking for a subset of male offspring, measured at around 18 years old. Intelligence was scored between 1 and 9 based on tests of synonyms, logic, spatial ability and technical knowledge^48^. Non-cognitive ability was also scored between 1 and 9 and assessed willingness to assume responsibility, independence, outgoing character, persistence, emotional stability and power of initiative^48^. Conscription medical examinations were compulsory from 1969 until 2001, except for those with a severe handicap or chronic disease. Smoking was only recorded in 1969–1970. The study was approved (number 2016/5:5) by the Ethical Review Board in Stockholm, who operate according to Swedish national law and European guidelines and do not require informed consent for research based on non-identifiable register-based data.

### Descriptive statistics

Paternal and maternal age were put into categories of <20, 20–24, 25–29, 30–34, 35–39, 40–44 and ≥45 years, with the last two categories combined for maternal age due to the scarcity of mothers aged over 45. Various demographic, socio-economic, and developmental variables measured in the offspring, mother, and father were summarised within each category of parental age. Linear or logistic regression with robust standard errors clustered by the parent’s identity was used to test for differences in each variable among categories. The associations of parental age with risk factors potentially associated with offspring health in this dataset have been reported more comprehensively elsewhere^8^.

### Primary analyses

In the primary statistical analyses, Cox proportional hazards regression with offspring age as the time axis was used to estimate hazard ratios for all-cause and cause-specific mortality per 5 years of parental age. Maternal and paternal age were analysed separately as the exposure of interest. Male and female offspring were analysed together in the main analyses except for sex-specific outcomes. Robust standard errors clustered by the identity of the parent in question were used to account for clustering within families. Analyses were conducted with five alternative adjustment sets. Adjustment (a) consisted of no adjustment; the age of the parent in question at offspring birth was the only covariate. Adjustment (b) consisted of the offspring’s sex and DOB (as a linear covariate). Adjustment (c) additionally adjusted for the occupational SEP and educational level of both parents. Adjustment (d) additionally adjusted for the offspring’s birth order. Adjustment (e) consisted of adjustment (d), plus adjustment for the other parent’s age at the time of the offspring’s birth as a linear covariate. When adjustment (e) was used, the resulting coefficients for maternal and paternal age were compared using a Wald test. Offspring sex was removed from the adjustment set when analyses were restricted to offspring of one sex.

### Sibling-comparison analyses

A range of secondary analyses were used to look further into the association between parental age and mortality. To estimate within-family associations between mortality and parental age avoiding all measured and unmeasured family-level confounding, Cox proportional hazards models were repeated, with stratification by the identity of the parent in question. Adjustment covariates which are invariant or almost invariant within families (i.e. the occupational SEP and educational level of both parents) cannot be included in such models but equally cannot confound a within-family association. More problematically, the exposure of interest (the age of the parent at the time of the offspring’s birth) is perfectly co-linear with the offspring’s DOB within families. Individual-level secular trends therefore cannot be adjusted for in these sibling-comparison models^8,15,16^ although secular trends at the family level are automatically adjusted for. For similar reasons, these models also cannot be adjusted for the other parent’s age at the offspring’s birth. Sibling comparison models were thus adjusted for offspring sex and birth order only.

To further investigate the role of secular trends (and adjustment for them) in associations between parental age and offspring survival, the primary analysis was first repeated with adjustment (e) but omitting the exposure (age of the parent in question at the offspring’s birth) and the association between offspring DOB and survival was noted as a measure of secular trends. Secondly, the primary analysis was repeated with adjustment (e) except that the DOB of the parent in question was used in place of the offspring’s DOB. This reduced the likely family-level confounding by secular trends but did not remove the individual-level mediation by them.

Sibling comparison studies of within-family associations are necessarily restricted to families of ≥2 offspring in which both the exposure and the outcome are discordant. In a survival analysis, this means restriction to those offspring who either (i) died at an age when a sibling was also at risk or (ii) were at risk at the age when a sibling died. A restricted dataset consisting of such offspring was created for each sibling-comparison analysis (sample sizes varied according to outcome). To separate the methodological effects of the sibling-comparison analysis from the effects of this sample restriction, the primary analysis was repeated using this restricted dataset.

### Two-variable analyses

As an alternative way to avoid family-level confounding, it has been proposed^49^ that an analysis of parental age effects should be adjusted for the parent’s age at the birth of their first child. In this “two-variable” analysis, the association with parental age at their first child’s birth can be interpreted as an estimate of the family-level confounding and the association with parental age at the index child’s birth is an estimate of the association adjusted for such confounding (although there is a risk of induced confounding^8^). Such analyses necessarily exclude first-born offspring, so the primary analyses were also repeated separately for first-born and subsequent offspring to see if the associations between parental age and survival differed between these groups. An analysis of first-born offspring also has the advantages of (i) being a purely between-family analysis to compare with the within-family associations of the sibling comparison analysis and the overall associations of the primary analysis and (ii) being invulnerable to the effects of “stoppage”, whereby parents having had an unhealthy child decide against having further children^50^.

### Other sensitivity analyses

Prior to about 1970, parental age at the birth of offspring was in a long-term decline similar in gradient to the subsequent rise^8^. Two such distinct periods with strong secular trends in the exposure make confounding and/or mediation by secular trends particularly likely. To address this, we repeated the primary and sibling comparison analyses with offspring born on or before 31^st^ December 1969 analysed separately from those born on or after 1^st^ January 1970.

The primary and sibling-comparison analyses were repeated with restriction to male or female offspring, to assess the contribution of survival in each sex to the overall results. Adjustment for sex was omitted from these models and the dataset for the sibling-comparison analysis was further restricted to those offspring from discordant same-sex sibling groups.

To assess whether associations between offspring survival and parental age were mediated by intergenerational lifespan overlap^18^, the primary analyses were repeated on those offspring for whom it could be determined whether or not the parent in question lived until the offspring’s 35^th^ birthday. Parental survival follow-up was available from 1^st^ January 1952 until 31^st^ December 2012, so offspring born before this period, or not yet 35 at the end of it, were excluded from this analysis. Analyses on this reduced data set were run with and without adjustment for a binary variable indicating parental survival to the offspring’s 35^th^ birthday.

Parental age effects on offspring survival might be mediated by the offspring’s size at birth. In the subset of offspring (born in 1973 or later) where these data were available, we repeated the primary analyses with and without adjustment for birth weight and length.

To assess the shape of associations between offspring mortality and parental age, the primary and sibling comparison analyses were repeated with parental age divided into categories (as defined for descriptive statistics above), and the associations plotted. If a category of parental age included fewer than 5 deaths, offspring in that category were excluded from the analysis. The sibling comparison analysis by categories of parental age was also necessarily restricted to offspring in families discordant for the exposure (i.e. parental age spanned more than one category).

The proportional hazards assumption was tested by measuring the correlation between log(age) and the scaled Schoenfeld residuals for parental age from the model with adjustment (e). To further examine any departures from the proportional hazards assumption, the analyses were repeated with follow-up divided into four stages according to the offspring’s age (divisions at 40, 50 and 60 years) and a Wald test used to compare the hazard ratios within each age class.

Sensitivity analyses were omitted when the data restrictions resulted in the analysis of fewer than 20 deaths. Analyses were performed using Stata 15.1 on a desktop machine and Stata 14.1 on the University of Bristol’s Blue Crystal high power computing cluster.

## Supplementary information

Supplementary Material

## Data Availability

Swedish privacy laws prohibit us from making individual-level data publicly available. Researchers who are interested in replicating our work using individual-level data should apply to the appropriate Swedish authorities e.g. Statistics Sweden. For more information, see https://www.scb.se/en/services/guidance-for-researchers-and-universities/.
