# Supplementary material for: Associations of parental age with offspring all-cause and cause-specific adult mortality
Source: Sci Rep. 2019 Nov 19;9:17097. doi: 10.1038/s41598-019-52853-8 (PMC6864242; doi:10.1038/s41598-019-52853-8)
Supplement: Supplementary file 1 — Supplementary Material [file 41598_2019_52853_MOESM1_ESM.pdf]

Supplementary Material

Associations of parental age with offspring all-cause and cause-specific adult mortality

David Carslake[1,2]*, Per Tynelius[3], Gerard J. van den Berg[4] and George Davey Smith[1,2]

[1]MRC Integrative Epidemiology Unit at the University of Bristol, Bristol, UK, [2]Population Health Sciences, Bristol Medical School, Bristol, UK, [3]Department of Public Health Sciences, Karolinska Institute, Stockholm, Sweden, [4]School of Economics, Finance and Management, University of Bristol, Bristol, UK.
*Correspondence and requests for materials should be addressed to D. C. (email: David.Carslake@bristol.ac.uk)

Table S1. Conversion of ICD codes to descriptive categories.
Table S2. Primary analyses of offspring mortality and maternal age with alternative adjustment sets.
Table S3. Primary analyses of offspring mortality and paternal age with alternative adjustment sets.
Table S4. Analyses decomposing the difference between the primary and sibling-comparison analyses of offspring mortality and maternal age.
Table S5. Analyses decomposing the difference between the primary and sibling-comparison analyses of offspring mortality and paternal age.
Table S6. Analyses of offspring mortality and maternal age, conducted separately for offspring who were the oldest child in their family and for others. Two-variable analyses of later children were also conducted with mutual adjustment for mother's age at the birth of (i) the child in question, and (ii) her first child.
Table S7. Analyses of offspring mortality and paternal age, conducted separately for offspring who were the oldest child in their family and for others. Two-variable analyses of later children were also conducted with mutual adjustment for father's age at the birth of (i) the child in question, and (ii) his first child.
Table S8. Primary analyses of offspring mortality and maternal age, conducted separately for offspring born up to 1969 (when parental age was decreasing) and for offspring born from 1970 (when parental age was increasing).
Table S9. Primary analyses of offspring mortality and paternal age, conducted separately for offspring born up to 1969 (when parental age was decreasing) and for offspring born from 1970 (when parental age was increasing).
Table S10. Sibling comparison analyses of offspring mortality and maternal age, conducted separately for offspring born up to 1969 (when parental age was decreasing) and for offspring born from 1970 (when parental age was increasing).
Table S11. Sibling comparison analyses of offspring mortality and paternal age, conducted separately for offspring born up to 1969 (when parental age was decreasing) and for offspring born from 1970 (when parental age was increasing).
Table S12. Primary analyses of son's outcomes and maternal age, with and without additional adjustment for maternal survival to the offspring's 35th birthday.
Table S13. Primary analyses of son's outcomes and paternal age, with and without additional adjustment for paternal survival to the offspring's 35th birthday.
Table S14. Primary analyses of offspring mortality and maternal age, with and without additional adjustment for offspring birth weight and birth length.
Table S15. Primary analyses of offspring mortality and paternal age, with and without additional adjustment for offspring birth weight and birth length.
Table S16. Associations between offspring mortality and maternal age from primary and sibling comparison analyses, including the potentially biased subset of offspring who died between 1961 and 30th June 1991 (N=56,901).

Table S17. Associations between offspring mortality and paternal age from primary and sibling comparison analyses, including the potentially biased subset of offspring who died between 1961 and 30th June 1991 (N=56,901).
Table S18. Primary analyses of offspring mortality and maternal age, with follow-up split by categories of offspring age.
Table S19. Primary analyses of offspring mortality and paternal age, with follow-up split by categories of offspring age.
Table S20. Primary and sibling-comparison analyses of offspring mortality and maternal age, made separately for men (sons) and women (daughters).
Table S21. Primary and sibling-comparison analyses of offspring mortality and paternal age, made separately for men (sons) and women (daughters).

Figure S1. Recorded all-cause and cause-specific deaths by year of death.
Figure S2. Hazard ratios for offspring mortality against classes of maternal or paternal age.

Supplementary Note. Comparison with Barclay & Myrskylä 2018.

Table S1. Conversion of ICD codes to descriptive categories. Mortality was recorded using ICD 7 (1961-1968), ICD 8 (1969-1986), ICD 9 (1987-1996) and ICD 10 (1997-2012). ICD codes are in alphabetical (not numerical) order such that, for example, 451>45099. Alzheimer’s disease includes all deaths recorded as dementia. Table continues on next page.

Cause of death	ICD 7	ICD 8	ICD 9	ICD 10
All-cause	(all codes)	(all codes)	(all codes)	(all codes)
Cardiovascular disease	330-33499<br>400-41699<br>420-42299<br>430-44799<br>450-46899<br>782-78299	390-40499<br>410-41499<br>420-42909<br>4299-43899<br>440-45899<br>782-78299	390-40599<br>410-43899<br>440-45999	G45-G4599<br>I00-I1599<br>I20-I5299<br>I60-I9999
Coronary heart disease	420-42099<br>422-42299<br>450-45099	410-41499<br>4299-42999	410-41499<br>4292-42929	I20-I2599<br>I516-I5169
Aortic aneurysm	451-45199	441-44199	441-44199	I71-I7199
Stroke	306-30699<br>330-33499<br>352-35299	2930-29319<br>430-43899<br>344-34499	2904-29049<br>342-34299<br>344-34499<br>430-43899	F01-F0199<br>G45-G4599<br>G81-G8199<br>G83-G8399<br>I60-I6999
Diabetes	260-26099	250-25099	250-25099	E10-E1499
Respiratory diseases	240-24199<br>470-52799	460-51999	460-51999	J00-J9999
Kidney disease	590-60499	580-59499	580-59499	N00-N2999
Pancreatic disease	587-58729	577-57799	577-57799	K85-K8699
Liver cirrhosis	581-58119	571-57109<br>5718-57199	5712-57129<br>5715-57159	K703-K7039<br>K746-K7469
Alcohol dependence	322-32299	303-30399	303-30399	F10-F1099
Alzheimer's disease	304-30599	290-29099	290-29039<br>2905-29099<br>3310-33109	F00-F0099<br>F03-F0399<br>G30-G3099
Parkinson's disease	350-35099	342-34299	332-33209	G20-G2099
Multiple sclerosis	345-34599	340-34099	340-34099	G35-G3599
Motor neurone disease	356-35699	348-34899	3352-33529	G122-<br>G1229
External causes	E800-E9999	E800-E9999	E800-E9999	V01-Y9899
Suicide	E970-E9799	E950-E9599	E950-E9599	X60-X8499

Table S1 (continued).

Cause of death	ICD 7	ICD 8	ICD 9	ICD 10
Any cancer	140-20799	140-20799	140-20939	C00-C9799
Malignant melanoma	190-19099	172-17299	172-17299	C43-C4399
Brain cancer	193-19399	191-19299	191-19299	C71-C7299
Oesophageal cancer	150-15099	150-15099	150-15099	C15-C1599
Thyroid cancer	194-19499	193-19399	193-19399	C73-C7399
Lymphatic cancer	200-20799	200-20799	200-20899	C81-C9699
Lung cancer	162-16219<br>1628-16399	162-16299	162-16299	C33-C3499
Breast cancer	170-17099	174-17499	174-17599	C50-C5099
Liver cancer	155-15509<br>1552-15599	155-15599	155-15599	C22-C2299
Gallbladder cancer	1551-15519	156-15699	156-15699	C23-C2499
Stomach cancer	151-15199	151-15199	151-15199	C16-C1699
Kidney cancer	180-18099	1890-18929	1890-18929	C64-C6699
Pancreatic cancer	157-15799	157-15799	157-15799	C25-C2599
Colorectal cancer	153-15499	153-15499	153-15499	C18-C2199
Bladder cancer	1810-18109	188-18899	188-18899	C67-C6799
Ovarian cancer	175-17599	183-18399	183-18399	C56-C5699<br>C570-C5749
Uterine cancer	171-17499	180-18209<br>1829-18299	179-18099<br>181-18289	C53-C5599
Prostate cancer	177-17799	185-18599	185-18599	C61-C6199
Testicular cancer	178-17899	186-18699	186-18699	C62-C6299

Table S2. Primary analyses of maternal age and offspring mortality with alternative adjustment sets. Adjustment set (a) was empty, (b) comprised offspring sex and date of birth, (c) additionally included educational and occupational SEP in both parents and (d) additionally included birth order. Adjustment set (e) additionally included paternal age as a linear term. Hazard ratios (95% CI) are from Cox proportional hazards regression of 2,658,132 male and 2,546,301 female offspring with age as the time axis. Maternal and paternal associations in model (e) were compared using P-values from a Wald test.

		**Primary analysis hazard ratio per five years of mother's age at offspring's birth with adjustment set:**					**Parental**
**Outcome**	**Deaths**	**a**	**b**	**c**	**d**	**e**	**comparison**
All-cause	293,470	0.98 (0.98, 0.99)	0.98 (0.98, 0.98)	0.95 (0.95, 0.96)	0.94 (0.94, 0.95)	0.96 (0.96, 0.97)	<0.001
Cardiovascular disease	76,352	0.98 (0.97, 0.99)	0.97 (0.97, 0.98)	0.93 (0.92, 0.94)	0.93 (0.92, 0.94)	0.95 (0.94, 0.96)	0.020
Coronary heart disease	41,853	0.99 (0.98, 0.99)	0.98 (0.97, 0.99)	0.93 (0.92, 0.94)	0.92 (0.91, 0.93)	0.95 (0.93, 0.96)	0.278
Aortic aneurysm	3,974	0.94 (0.91, 0.96)	0.93 (0.91, 0.96)	0.91 (0.88, 0.95)	0.91 (0.88, 0.95)	0.92 (0.88, 0.96)	0.055
Stroke	14,692	0.97 (0.96, 0.98)	0.97 (0.95, 0.98)	0.92 (0.90, 0.94)	0.92 (0.90, 0.94)	0.94 (0.92, 0.96)	0.175
Diabetes	5,956	0.95 (0.93, 0.97)	0.95 (0.93, 0.97)	0.93 (0.91, 0.96)	0.94 (0.91, 0.97)	0.95 (0.91, 0.98)	0.158
Respiratory diseases	12,187	0.98 (0.96, 0.99)	0.97 (0.96, 0.99)	0.95 (0.93, 0.97)	0.93 (0.91, 0.95)	0.96 (0.93, 0.99)	0.789
Kidney disease	1,271	0.99 (0.94, 1.03)	0.98 (0.94, 1.03)	0.94 (0.88, 1.00)	0.93 (0.87, 0.99)	0.96 (0.89, 1.04)	0.891
Pancreatic disease	848	0.93 (0.88, 0.99)	0.93 (0.87, 0.98)	0.93 (0.86, 1.00)	0.91 (0.84, 0.98)	0.95 (0.86, 1.05)	0.792
Liver cirrhosis	4,440	0.92 (0.90, 0.95)	0.92 (0.90, 0.95)	0.92 (0.89, 0.95)	0.91 (0.88, 0.94)	0.99 (0.95, 1.04)	0.002
Alcohol dependence	4,101	0.98 (0.95, 1.00)	0.96 (0.94, 0.99)	0.97 (0.94, 1.00)	0.93 (0.89, 0.96)	0.96 (0.92, 1.00)	0.840
Alzheimer's disease	2,873	1.05 (1.02, 1.09)	1.05 (1.02, 1.09)	1.04 (0.99, 1.09)	1.04 (0.99, 1.09)	1.04 (0.98, 1.10)	0.373
Parkinson's disease	713	1.09 (1.03, 1.16)	1.09 (1.03, 1.16)	1.06 (0.97, 1.16)	1.07 (0.98, 1.17)	1.11 (0.99, 1.23)	0.104
Multiple sclerosis	1,296	1.00 (0.96, 1.05)	1.00 (0.96, 1.05)	1.00 (0.95, 1.06)	1.04 (0.98, 1.11)	1.01 (0.94, 1.09)	0.640
Motor neurone disease	1,914	0.99 (0.95, 1.03)	0.99 (0.96, 1.03)	0.97 (0.92, 1.02)	0.97 (0.92, 1.02)	0.96 (0.90, 1.02)	0.310
External causes	38,658	0.95 (0.95, 0.96)	0.95 (0.94, 0.96)	0.93 (0.92, 0.94)	0.89 (0.88, 0.90)	0.90 (0.89, 0.92)	<0.001
Suicide	15,565	1.01 (0.99, 1.02)	1.01 (0.99, 1.02)	0.98 (0.97, 1.00)	0.93 (0.92, 0.95)	0.93 (0.91, 0.95)	<0.001
Any cancer	113,597	0.99 (0.98, 0.99)	0.99 (0.98, 0.99)	0.98 (0.97, 0.98)	0.97 (0.96, 0.97)	0.98 (0.97, 0.99)	0.172
Malignant melanoma	3,472	0.97 (0.95, 1.00)	0.97 (0.95, 1.00)	0.98 (0.95, 1.01)	1.01 (0.98, 1.05)	1.05 (1.00, 1.10)	0.013
Brain cancer	5,824	1.00 (0.97, 1.02)	0.99 (0.97, 1.01)	0.99 (0.96, 1.01)	0.99 (0.96, 1.02)	0.97 (0.94, 1.00)	0.047
Oesophageal cancer	2,482	0.97 (0.94, 1.00)	0.97 (0.94, 1.01)	0.98 (0.94, 1.02)	0.98 (0.94, 1.03)	0.97 (0.92, 1.03)	0.487
Thyroid cancer	323	0.89 (0.81, 0.99)	0.89 (0.81, 0.99)	0.85 (0.75, 0.97)	0.84 (0.73, 0.95)	0.83 (0.72, 0.96)	0.120
Lymphatic cancer	9,408	1.02 (1.00, 1.03)	1.01 (1.00, 1.03)	1.00 (0.98, 1.03)	1.00 (0.98, 1.02)	1.01 (0.98, 1.03)	0.548
Lung cancer	20,752	0.97 (0.96, 0.98)	0.97 (0.96, 0.98)	0.96 (0.95, 0.98)	0.93 (0.91, 0.94)	0.94 (0.93, 0.96)	0.080
Breast cancer	10,887	1.04 (1.02, 1.05)	1.03 (1.02, 1.05)	1.02 (1.00, 1.04)	1.03 (1.01, 1.05)	1.02 (0.99, 1.05)	0.920
Liver cancer	3,053	0.95 (0.92, 0.98)	0.95 (0.92, 0.98)	0.92 (0.88, 0.96)	0.91 (0.88, 0.95)	0.97 (0.92, 1.02)	0.242
Gallbladder cancer	2,626	1.03 (0.99, 1.06)	1.02 (0.99, 1.06)	0.97 (0.93, 1.01)	0.98 (0.94, 1.03)	1.01 (0.95, 1.06)	0.349
Stomach cancer	3,580	0.99 (0.96, 1.02)	0.98 (0.95, 1.01)	0.94 (0.91, 0.97)	0.92 (0.88, 0.95)	0.90 (0.86, 0.94)	<0.001
Kidney cancer	3,515	0.94 (0.91, 0.97)	0.93 (0.91, 0.96)	0.92 (0.89, 0.96)	0.92 (0.88, 0.95)	0.92 (0.88, 0.97)	0.105
Pancreatic cancer	8,258	0.98 (0.97, 1.00)	0.98 (0.97, 1.00)	0.97 (0.95, 1.00)	0.96 (0.94, 0.99)	0.98 (0.95, 1.01)	0.987
Colorectal cancer	12,325	0.98 (0.96, 0.99)	0.98 (0.96, 0.99)	0.96 (0.94, 0.97)	0.96 (0.94, 0.98)	0.96 (0.94, 0.99)	0.070

Bladder cancer	2,173	0.99 (0.96, 1.03)	0.99 (0.96, 1.03)	1.00 (0.96, 1.05)	1.00 (0.95, 1.05)	1.01 (0.95, 1.07)	0.654
Ovarian cancer	4,682	1.04 (1.01, 1.06)	1.03 (1.01, 1.06)	0.99 (0.97, 1.03)	0.99 (0.96, 1.03)	0.99 (0.95, 1.03)	0.778
Uterine cancer	2,672	0.99 (0.96, 1.03)	0.99 (0.96, 1.03)	0.95 (0.91, 0.98)	0.96 (0.92, 1.00)	0.96 (0.91, 1.01)	0.363
Prostate cancer	5,919	1.01 (0.99, 1.03)	1.01 (0.99, 1.03)	1.01 (0.98, 1.04)	1.01 (0.98, 1.04)	1.03 (0.99, 1.07)	0.079
Testicular cancer	133	1.02 (0.86, 1.20)	1.01 (0.86, 1.19)	1.01 (0.86, 1.19)	1.08 (0.91, 1.28)	1.12 (0.87, 1.44)	0.480

Table S3. Primary analyses of paternal age and offspring mortality with alternative adjustment sets. Adjustment set (a) was empty, (b) comprised offspring sex and date of birth, (c) additionally included educational and occupational SEP in both parents and (d) additionally included birth order. Adjustment set (e) additionally included maternal age as a linear term. Hazard ratios (95% CI) are from Cox proportional hazards regression of 2,658,132 male and 2,546,301 female offspring with age as the time axis. Maternal and paternal associations in model (e) were compared using P-values from a Wald test.

		Primary analysis hazard ratio per five years of father's age at offspring's birth with adjustment set:					Parental
**Outcome**	**Deaths**	**a**	**b**	**c**	**d**	**e**	**comparison**
All-cause	293,470	0.99 (0.99, 1.00)	0.99 (0.99, 0.99)	0.96 (0.96, 0.97)	0.96 (0.95, 0.96)	0.98 (0.97, 0.98)	<0.001
Cardiovascular disease	76,352	1.00 (0.99, 1.00)	0.99 (0.98, 1.00)	0.94 (0.94, 0.95)	0.94 (0.94, 0.95)	0.97 (0.96, 0.98)	0.020
Coronary heart disease	41,853	1.00 (0.99, 1.01)	0.99 (0.99, 1.00)	0.94 (0.93, 0.95)	0.93 (0.93, 0.94)	0.96 (0.95, 0.97)	0.278
Aortic aneurysm	3,974	0.97 (0.94, 0.99)	0.96 (0.94, 0.98)	0.95 (0.92, 0.98)	0.95 (0.92, 0.98)	0.99 (0.95, 1.03)	0.055
Stroke	14,692	0.99 (0.98, 1.00)	0.98 (0.97, 0.99)	0.94 (0.92, 0.95)	0.94 (0.92, 0.95)	0.97 (0.95, 0.99)	0.175
Diabetes	5,956	0.97 (0.95, 0.99)	0.97 (0.95, 0.99)	0.95 (0.93, 0.98)	0.96 (0.94, 0.99)	0.99 (0.96, 1.02)	0.158
Respiratory diseases	12,187	0.99 (0.97, 1.00)	0.98 (0.97, 1.00)	0.95 (0.93, 0.97)	0.93 (0.92, 0.95)	0.95 (0.93, 0.98)	0.789
Kidney disease	1,271	0.99 (0.95, 1.03)	0.98 (0.95, 1.03)	0.94 (0.89, 0.99)	0.93 (0.88, 0.99)	0.95 (0.89, 1.02)	0.891
Pancreatic disease	848	0.94 (0.89, 0.99)	0.94 (0.89, 0.99)	0.92 (0.87, 0.98)	0.91 (0.85, 0.97)	0.93 (0.86, 1.01)	0.792
Liver cirrhosis	4,440	0.91 (0.89, 0.93)	0.91 (0.89, 0.93)	0.89 (0.86, 0.92)	0.88 (0.85, 0.91)	0.88 (0.85, 0.92)	0.002
Alcohol dependence	4,101	0.98 (0.96, 1.01)	0.97 (0.95, 0.99)	0.96 (0.94, 0.99)	0.93 (0.90, 0.96)	0.95 (0.91, 0.99)	0.840
Alzheimer's disease	2,873	1.03 (1.01, 1.06)	1.03 (1.01, 1.06)	1.02 (0.98, 1.06)	1.02 (0.98, 1.06)	1.00 (0.95, 1.05)	0.373
Parkinson's disease	713	1.05 (1.00, 1.11)	1.05 (1.00, 1.11)	0.98 (0.91, 1.07)	0.99 (0.91, 1.07)	0.95 (0.86, 1.05)	0.104
Multiple sclerosis	1,296	1.02 (0.98, 1.06)	1.02 (0.98, 1.05)	1.02 (0.97, 1.07)	1.05 (1.00, 1.10)	1.04 (0.98, 1.11)	0.640
Motor neurone disease	1,914	1.01 (0.97, 1.04)	1.01 (0.98, 1.04)	0.99 (0.95, 1.03)	0.99 (0.95, 1.03)	1.01 (0.96, 1.07)	0.310
External causes	38,658	0.98 (0.98, 0.99)	0.98 (0.97, 0.99)	0.96 (0.95, 0.96)	0.93 (0.92, 0.94)	0.99 (0.97, 1.00)	<0.001
Suicide	15,565	1.03 (1.02, 1.04)	1.02 (1.01, 1.04)	1.00 (0.98, 1.01)	0.97 (0.95, 0.98)	1.01 (0.99, 1.03)	<0.001
Any cancer	113,597	0.99 (0.99, 1.00)	0.99 (0.99, 1.00)	0.98 (0.98, 0.99)	0.97 (0.97, 0.98)	0.99 (0.98, 0.99)	0.172
Malignant melanoma	3,472	0.96 (0.93, 0.98)	0.96 (0.94, 0.98)	0.95 (0.93, 0.98)	0.98 (0.95, 1.01)	0.95 (0.91, 0.99)	0.013
Brain cancer	5,824	1.01 (0.99, 1.02)	1.00 (0.99, 1.02)	1.01 (0.99, 1.03)	1.01 (0.99, 1.04)	1.03 (1.00, 1.06)	0.047
Oesophageal cancer	2,482	0.99 (0.96, 1.02)	0.99 (0.96, 1.02)	0.99 (0.95, 1.03)	1.00 (0.96, 1.04)	1.01 (0.96, 1.06)	0.487
Thyroid cancer	323	0.95 (0.88, 1.04)	0.95 (0.88, 1.04)	0.92 (0.83, 1.03)	0.92 (0.82, 1.03)	1.00 (0.89, 1.14)	0.120
Lymphatic cancer	9,408	1.01 (1.00, 1.03)	1.01 (0.99, 1.02)	1.00 (0.98, 1.02)	0.99 (0.98, 1.01)	0.99 (0.97, 1.02)	0.548
Lung cancer	20,752	0.98 (0.97, 0.99)	0.98 (0.97, 0.99)	0.97 (0.96, 0.99)	0.95 (0.93, 0.96)	0.97 (0.96, 0.99)	0.080
Breast cancer	10,887	1.03 (1.01, 1.04)	1.02 (1.01, 1.04)	1.02 (1.00, 1.04)	1.03 (1.01, 1.05)	1.02 (0.99, 1.04)	0.920
Liver cancer	3,053	0.96 (0.93, 0.98)	0.95 (0.93, 0.98)	0.91 (0.88, 0.94)	0.90 (0.87, 0.94)	0.92 (0.88, 0.96)	0.242
Gallbladder cancer	2,626	1.02 (1.00, 1.05)	1.02 (0.99, 1.05)	0.96 (0.93, 1.00)	0.97 (0.93, 1.01)	0.97 (0.92, 1.01)	0.349
Stomach cancer	3,580	1.02 (0.99, 1.04)	1.01 (0.99, 1.03)	0.99 (0.96, 1.02)	0.97 (0.94, 1.01)	1.03 (0.99, 1.07)	<0.001
Kidney cancer	3,515	0.96 (0.94, 0.99)	0.96 (0.93, 0.98)	0.95 (0.92, 0.98)	0.95 (0.92, 0.98)	0.99 (0.95, 1.03)	0.105
Pancreatic cancer	8,258	0.98 (0.97, 1.00)	0.98 (0.97, 1.00)	0.97 (0.95, 1.00)	0.96 (0.94, 0.99)	0.98 (0.95, 1.00)	0.987

Colorectal cancer	12,325	0.99 (0.98, 1.00)	0.99 (0.98, 1.00)	0.97 (0.96, 0.99)	0.98 (0.96, 1.00)	1.00 (0.98, 1.02)	0.070
Bladder cancer	2,173	0.99 (0.96, 1.02)	0.98 (0.95, 1.01)	0.99 (0.95, 1.04)	0.99 (0.95, 1.03)	0.99 (0.93, 1.04)	0.654
Ovarian cancer	4,682	1.03 (1.01, 1.05)	1.02 (1.00, 1.04)	1.00 (0.97, 1.03)	1.00 (0.97, 1.03)	1.00 (0.97, 1.04)	0.778
Uterine cancer	2,672	1.00 (0.98, 1.03)	1.00 (0.97, 1.03)	0.97 (0.93, 1.00)	0.98 (0.94, 1.01)	1.00 (0.95, 1.05)	0.363
Prostate cancer	5,919	1.01 (0.99, 1.03)	1.01 (0.99, 1.02)	0.99 (0.96, 1.01)	0.98 (0.96, 1.01)	0.97 (0.94, 1.00)	0.079
Testicular cancer	133	0.99 (0.87, 1.13)	0.99 (0.87, 1.13)	0.98 (0.85, 1.13)	1.02 (0.88, 1.18)	0.95 (0.76, 1.19)	0.480

Table S4. Analyses decomposing the difference between the primary and sibling-comparison analyses of offspring mortality and maternal age. Primary analyses used Cox proportional hazards regression of 2,658,132 male and 2,546,301 female offspring. Age was the time axis and robust standard errors were clustered by maternal identity. Adjustment set (e) (offspring sex and date of birth (DOB), maternal and paternal occupational and educational SEP, offspring birth order, and paternal age) was used. The secular trend per five years of offspring DOB was assessed using a similar model but without maternal age. The primary analysis was repeated with adjustment for maternal, not offspring, DOB to account for confounding, but not mediation, by secular trends. To examine whether the restricted dataset used for sibling-comparison analyses was representative of the main dataset, the primary analysis was repeated on this subset. Finally, the sibling-comparison analysis used Cox regression stratified by maternal identity and was restricted to offspring in families with discordant outcomes. All family-level confounding was intrinsically adjusted for and adjustment for offspring DOB or paternal age were not possible. Explicit adjustment was therefore limited to offspring sex and birth order.

				**Hazard ratio per five years of mother's age at offspring's birth (or per five years of offspring's DOB for secular trend)**				
**Outcome**	**Deaths (main data)**	**Deaths (restricted data)**	**N (restricted data)**	**Primary analysis (main data)**	**Secular trend (main data)**	**Primary analysis plus mediation (main data)**	**Primary analysis (restricted data)**	**Sibling-comparison analysis (restricted data)**
All-cause	293,470	190,795	449,224	0.96 (0.96, 0.97)	0.94 (0.94, 0.94)	0.91 (0.90, 0.91)	1.03 (1.03, 1.04)	0.94 (0.93, 0.96)
Cardiovascular disease	76,352	45,734	118,683	0.95 (0.94, 0.96)	0.88 (0.87, 0.88)	0.83 (0.82, 0.85)	1.03 (1.02, 1.03)	0.89 (0.86, 0.92)
Coronary heart disease	41,853	25,214	67,767	0.95 (0.93, 0.96)	0.84 (0.83, 0.85)	0.80 (0.79, 0.82)	1.02 (1.01, 1.03)	0.83 (0.79, 0.87)
Aortic aneurysm	3,974	2,346	6,396	0.92 (0.88, 0.96)	0.86 (0.83, 0.90)	0.80 (0.75, 0.85)	1.04 (1.00, 1.07)	0.87 (0.74, 1.01)
Stroke	14,692	8,391	22,973	0.94 (0.92, 0.96)	0.86 (0.84, 0.87)	0.81 (0.79, 0.83)	1.03 (1.02, 1.05)	0.84 (0.78, 0.91)
Diabetes	5,956	3,449	9,302	0.95 (0.91, 0.98)	0.98 (0.95, 1.01)	0.93 (0.89, 0.97)	1.06 (1.03, 1.09)	1.02 (0.91, 1.14)
Respiratory diseases	12,187	6,880	18,537	0.96 (0.93, 0.99)	0.92 (0.90, 0.95)	0.89 (0.86, 0.92)	1.04 (1.03, 1.06)	0.90 (0.83, 0.98)
Kidney disease	1,271	691	1,853	0.96 (0.89, 1.04)	0.94 (0.87, 1.01)	0.91 (0.82, 1.01)	1.02 (0.98, 1.07)	0.92 (0.71, 1.20)
Pancreatic disease	848	521	1,454	0.95 (0.86, 1.05)	0.92 (0.85, 0.99)	0.88 (0.78, 0.99)	1.07 (0.99, 1.16)	1.05 (0.81, 1.38)
Liver cirrhosis	4,440	2,710	7,404	0.99 (0.95, 1.04)	0.99 (0.95, 1.02)	0.98 (0.93, 1.03)	1.03 (1.00, 1.06)	0.95 (0.83, 1.08)
Alcohol dependence	4,101	2,822	8,208	0.96 (0.92, 1.00)	0.82 (0.79, 0.84)	0.78 (0.74, 0.83)	1.04 (1.01, 1.08)	0.82 (0.72, 0.93)
Alzheimer's disease	2,873	1,244	3,172	1.04 (0.98, 1.10)	1.05 (0.98, 1.12)	1.08 (0.99, 1.17)	1.06 (1.02, 1.11)	1.21 (0.97, 1.51)
Parkinson's disease	713	290	735	1.11 (0.99, 1.23)	1.21 (1.04, 1.41)	1.30 (1.10, 1.55)	1.08 (1.00, 1.18)	1.37 (0.81, 2.32)
Multiple sclerosis	1,296	800	2,130	1.01 (0.94, 1.09)	1.00 (0.94, 1.06)	1.01 (0.92, 1.11)	1.03 (0.98, 1.08)	0.94 (0.74, 1.18)
Motor neurone disease	1,914	1,163	3,201	0.96 (0.90, 1.02)	1.09 (1.03, 1.16)	1.05 (0.96, 1.14)	1.04 (1.00, 1.08)	0.98 (0.80, 1.20)
External causes	38,658	27,050	73,054	0.90 (0.89, 0.92)	0.98 (0.97, 0.98)	0.88 (0.86, 0.89)	1.07 (1.06, 1.08)	0.91 (0.87, 0.94)
Suicide	15,565	11,106	30,276	0.93 (0.91, 0.95)	0.97 (0.95, 0.98)	0.90 (0.87, 0.92)	1.07 (1.05, 1.08)	0.91 (0.86, 0.97)
Any cancer	113,597	71,881	184,833	0.98 (0.97, 0.99)	0.93 (0.92, 0.94)	0.91 (0.90, 0.92)	1.04 (1.04, 1.05)	0.94 (0.92, 0.97)
Malignant melanoma	3,472	2,171	5,834	1.05 (1.00, 1.10)	1.06 (1.02, 1.10)	1.11 (1.05, 1.18)	1.09 (1.06, 1.13)	1.06 (0.92, 1.23)
Brain cancer	5,824	3,996	10,871	0.97 (0.94, 1.00)	0.95 (0.92, 0.97)	0.92 (0.88, 0.96)	1.03 (1.01, 1.05)	0.96 (0.87, 1.07)
Oesophageal cancer	2,482	1,455	3,966	0.97 (0.92, 1.03)	1.04 (0.98, 1.09)	1.01 (0.94, 1.09)	1.04 (1.00, 1.09)	1.09 (0.90, 1.31)
Thyroid cancer	323	207	554	0.83 (0.72, 0.96)	1.02 (0.88, 1.19)	0.87 (0.71, 1.06)	1.08 (0.97, 1.20)	1.37 (0.87, 2.17)
Lymphatic cancer	9,408	5,781	15,854	1.01 (0.98, 1.03)	0.84 (0.82, 0.86)	0.84 (0.81, 0.87)	1.04 (1.02, 1.06)	0.93 (0.85, 1.02)

Lung cancer	20,752	12,998	35,452	0.94 (0.93, 0.96)	0.93 (0.91, 0.95)	0.88 (0.86, 0.91)	1.04 (1.03, 1.06)	0.94 (0.88, 0.99)
Breast cancer	10,887	4,587	10,862	1.02 (0.99, 1.05)	0.91 (0.89, 0.93)	0.93 (0.90, 0.96)	1.00 (0.98, 1.02)	0.90 (0.81, 0.99)
Liver cancer	3,053	1,818	4,988	0.97 (0.92, 1.02)	1.04 (0.99, 1.09)	1.01 (0.94, 1.08)	1.07 (1.03, 1.10)	0.94 (0.80, 1.11)
Gallbladder cancer	2,626	1,550	4,437	1.01 (0.95, 1.06)	0.95 (0.90, 1.00)	0.96 (0.89, 1.03)	1.03 (1.00, 1.07)	1.01 (0.85, 1.20)
Stomach cancer	3,580	2,343	6,712	0.90 (0.86, 0.94)	0.84 (0.81, 0.87)	0.76 (0.72, 0.81)	1.02 (0.99, 1.05)	0.80 (0.70, 0.92)
Kidney cancer	3,515	2,144	6,073	0.92 (0.88, 0.97)	0.81 (0.77, 0.84)	0.75 (0.71, 0.80)	1.02 (0.99, 1.05)	0.76 (0.66, 0.89)
Pancreatic cancer	8,258	4,993	13,777	0.98 (0.95, 1.01)	0.97 (0.94, 1.00)	0.95 (0.91, 0.99)	1.06 (1.04, 1.08)	0.95 (0.86, 1.04)
Colorectal cancer	12,325	7,357	19,886	0.96 (0.94, 0.99)	0.97 (0.95, 0.99)	0.94 (0.91, 0.97)	1.07 (1.05, 1.08)	0.99 (0.91, 1.07)
Bladder cancer	2,173	1,216	3,339	1.01 (0.95, 1.07)	0.94 (0.89, 1.00)	0.95 (0.87, 1.03)	1.06 (1.01, 1.10)	0.98 (0.79, 1.20)
Ovarian cancer	4,682	1,848	4,328	0.99 (0.95, 1.03)	0.92 (0.89, 0.95)	0.91 (0.87, 0.96)	1.03 (1.00, 1.07)	1.03 (0.88, 1.20)
Uterine cancer	2,672	1,004	2,412	0.96 (0.91, 1.01)	1.01 (0.97, 1.05)	0.97 (0.90, 1.04)	1.02 (0.97, 1.06)	1.05 (0.86, 1.29)
Prostate cancer	5,919	1,937	4,538	1.03 (0.99, 1.07)	0.87 (0.84, 0.91)	0.89 (0.84, 0.93)	0.99 (0.96, 1.02)	0.99 (0.83, 1.18)
Testicular cancer	133	45	104	1.12 (0.87, 1.44)	0.99 (0.86, 1.14)	1.11 (0.84, 1.47)	1.20 (0.94, 1.53)	1.64 (0.65, 4.10)

Table S5. Analyses decomposing the difference between the primary and sibling-comparison analyses of offspring mortality and paternal age. Primary analyses used Cox proportional hazards regression of 2,658,132 male and 2,546,301 female offspring. Age was the time axis and robust standard errors were clustered by paternal identity. Adjustment set (e) (offspring sex and date of birth (DOB), maternal and paternal occupational and educational SEP, offspring birth order, and maternal age) was used. The secular trend per five years of offspring DOB was assessed using a similar model but without paternal age. The primary analysis was repeated with adjustment for paternal, not offspring, DOB to account for confounding, but not mediation, by secular trends. To examine whether the restricted dataset used for sibling-comparison analyses was representative of the main dataset, the primary analysis was repeated on this subset. Finally, the sibling-comparison analysis used Cox regression stratified by paternal identity and was restricted to offspring in families with discordant outcomes. All family-level confounding was intrinsically adjusted for and adjustment for offspring DOB or maternal age were not possible. Explicit adjustment was therefore limited to offspring sex and birth order.

				Hazard ratio per five years of father's age at offspring's birth (or per five years of offspring's DOB for secular trend)				
**Outcome**	**Deaths (main data)**	**Deaths (restricted data)**	**N (restricted data)**	**Primary analysis (main data)**	**Secular trend (main data)**	**Primary analysis plus mediation (main data)**	**Primary analysis (restricted data)**	**Sibling-comparison analysis (restricted data)**
All-cause	293,470	190,579	451,278	0.98 (0.97, 0.98)	0.94 (0.94, 0.94)	0.92 (0.91, 0.93)	0.98 (0.98, 0.98)	0.93 (0.92, 0.94)
Cardiovascular disease	76,352	45,690	118,949	0.97 (0.96, 0.98)	0.88 (0.87, 0.88)	0.85 (0.84, 0.86)	0.98 (0.97, 0.99)	0.87 (0.84, 0.90)
Coronary heart disease	41,853	25,182	67,910	0.96 (0.95, 0.97)	0.84 (0.83, 0.85)	0.81 (0.80, 0.83)	0.98 (0.97, 0.98)	0.82 (0.79, 0.86)
Aortic aneurysm	3,974	2,351	6,418	0.99 (0.95, 1.03)	0.87 (0.83, 0.90)	0.86 (0.82, 0.91)	0.96 (0.94, 0.99)	0.86 (0.75, 0.99)
Stroke	14,692	8,356	22,951	0.97 (0.95, 0.99)	0.86 (0.84, 0.88)	0.83 (0.81, 0.86)	0.98 (0.96, 0.99)	0.81 (0.76, 0.87)
Diabetes	5,956	3,437	9,335	0.99 (0.96, 1.02)	0.98 (0.95, 1.01)	0.97 (0.93, 1.01)	0.99 (0.97, 1.01)	0.95 (0.86, 1.05)
Respiratory diseases	12,187	6,863	18,556	0.95 (0.93, 0.98)	0.92 (0.90, 0.94)	0.88 (0.86, 0.91)	0.99 (0.98, 1.01)	0.87 (0.80, 0.93)
Kidney disease	1,271	688	1,836	0.95 (0.89, 1.02)	0.94 (0.87, 1.01)	0.90 (0.82, 0.99)	0.98 (0.95, 1.02)	0.85 (0.66, 1.10)
Pancreatic disease	848	516	1,469	0.93 (0.86, 1.01)	0.92 (0.85, 0.99)	0.86 (0.77, 0.96)	0.98 (0.92, 1.05)	0.99 (0.78, 1.25)
Liver cirrhosis	4,440	2,722	7,525	0.88 (0.85, 0.92)	0.97 (0.94, 1.01)	0.87 (0.83, 0.91)	0.98 (0.95, 1.00)	0.93 (0.83, 1.03)
Alcohol dependence	4,101	2,816	8,207	0.95 (0.91, 0.99)	0.81 (0.79, 0.84)	0.78 (0.74, 0.82)	0.94 (0.92, 0.97)	0.73 (0.66, 0.82)
Alzheimer's disease	2,873	1,258	3,204	1.00 (0.95, 1.05)	1.04 (0.97, 1.11)	1.03 (0.96, 1.12)	0.99 (0.96, 1.02)	1.15 (0.93, 1.41)
Parkinson's disease	713	290	735	0.95 (0.86, 1.05)	1.16 (1.00, 1.35)	1.12 (0.94, 1.32)	1.01 (0.95, 1.08)	1.29 (0.76, 2.20)
Multiple sclerosis	1,296	808	2,173	1.04 (0.98, 1.11)	1.00 (0.94, 1.06)	1.04 (0.96, 1.13)	1.00 (0.96, 1.04)	0.95 (0.78, 1.16)
Motor neurone disease	1,914	1,162	3,222	1.01 (0.96, 1.07)	1.10 (1.04, 1.16)	1.11 (1.03, 1.19)	0.97 (0.94, 1.00)	0.90 (0.75, 1.07)
External causes	38,658	26,951	73,616	0.99 (0.97, 1.00)	0.97 (0.96, 0.98)	0.96 (0.95, 0.98)	0.97 (0.96, 0.98)	0.93 (0.90, 0.96)
Suicide	15,565	11,062	30,489	1.01 (0.99, 1.03)	0.97 (0.95, 0.98)	0.98 (0.95, 1.00)	0.98 (0.97, 0.99)	0.95 (0.90, 0.99)
Any cancer	113,597	71,927	185,758	0.99 (0.98, 0.99)	0.93 (0.92, 0.94)	0.92 (0.91, 0.93)	0.98 (0.98, 0.99)	0.94 (0.92, 0.96)
Malignant melanoma	3,472	2,202	5,957	0.95 (0.91, 0.99)	1.05 (1.02, 1.09)	1.01 (0.95, 1.06)	0.98 (0.95, 1.00)	1.09 (0.96, 1.24)
Brain cancer	5,824	4,001	10,921	1.03 (1.00, 1.06)	0.95 (0.92, 0.98)	0.98 (0.94, 1.02)	0.99 (0.98, 1.01)	0.96 (0.88, 1.06)
Oesophageal cancer	2,482	1,463	4,020	1.01 (0.96, 1.06)	1.04 (0.99, 1.10)	1.05 (0.98, 1.12)	0.98 (0.95, 1.02)	1.10 (0.93, 1.31)
Thyroid cancer	323	204	547	1.00 (0.89, 1.14)	1.04 (0.90, 1.21)	1.04 (0.87, 1.25)	0.99 (0.92, 1.07)	1.28 (0.84, 1.94)

Lymphatic cancer	9,408	5,780	15,870	0.99 (0.97, 1.02)	0.84 (0.81, 0.86)	0.83 (0.80, 0.86)	1.00 (0.99, 1.02)	0.94 (0.86, 1.02)
Lung cancer	20,752	12,939	35,479	0.97 (0.96, 0.99)	0.93 (0.91, 0.95)	0.91 (0.89, 0.93)	0.98 (0.97, 0.99)	0.91 (0.87, 0.96)
Breast cancer	10,887	4,630	10,967	1.02 (0.99, 1.04)	0.91 (0.89, 0.93)	0.93 (0.90, 0.95)	0.97 (0.95, 0.99)	0.92 (0.85, 1.00)
Liver cancer	3,053	1,821	5,008	0.92 (0.88, 0.96)	1.03 (0.98, 1.08)	0.96 (0.90, 1.01)	0.97 (0.94, 0.99)	0.92 (0.80, 1.05)
Gallbladder cancer	2,626	1,564	4,490	0.97 (0.92, 1.01)	0.95 (0.90, 1.00)	0.92 (0.86, 0.98)	0.98 (0.96, 1.01)	1.01 (0.87, 1.17)
Stomach cancer	3,580	2,339	6,743	1.03 (0.99, 1.07)	0.85 (0.82, 0.89)	0.87 (0.83, 0.92)	0.99 (0.97, 1.02)	0.87 (0.77, 0.98)
Kidney cancer	3,515	2,156	6,117	0.99 (0.95, 1.03)	0.81 (0.78, 0.85)	0.80 (0.76, 0.85)	0.97 (0.95, 1.00)	0.78 (0.69, 0.89)
Pancreatic cancer	8,258	4,984	13,797	0.98 (0.95, 1.00)	0.97 (0.94, 1.00)	0.95 (0.92, 0.99)	0.98 (0.97, 1.00)	0.91 (0.84, 1.00)
Colorectal cancer	12,325	7,354	20,003	1.00 (0.98, 1.02)	0.97 (0.95, 1.00)	0.97 (0.94, 1.00)	0.98 (0.97, 0.99)	0.95 (0.88, 1.02)
Bladder cancer	2,173	1,219	3,364	0.99 (0.93, 1.04)	0.94 (0.88, 0.99)	0.92 (0.86, 1.00)	0.96 (0.93, 0.99)	0.98 (0.81, 1.19)
Ovarian cancer	4,682	1,847	4,346	1.00 (0.97, 1.04)	0.92 (0.89, 0.95)	0.92 (0.88, 0.96)	0.95 (0.93, 0.98)	0.97 (0.84, 1.12)
Uterine cancer	2,672	1,013	2,431	1.00 (0.95, 1.05)	1.01 (0.97, 1.05)	1.01 (0.95, 1.07)	1.01 (0.98, 1.05)	1.03 (0.86, 1.24)
Prostate cancer	5,919	1,944	4,552	0.97 (0.94, 1.00)	0.86 (0.82, 0.90)	0.84 (0.80, 0.88)	0.98 (0.96, 1.01)	1.01 (0.85, 1.19)
Testicular cancer	133	45	103	0.95 (0.76, 1.19)	0.99 (0.86, 1.15)	0.95 (0.71, 1.26)	0.88 (0.68, 1.13)	1.49 (0.67, 3.33)

Table S6. Analyses of offspring mortality and maternal age, conducted separately for offspring who were the oldest child in their family and for others. Two-variable analyses of later children were also conducted with mutual adjustment for mother's age at the birth of (i) the child in question, and (ii) her first child. Primary analyses used Cox proportional hazards regression of 2,412,961 oldest offspring and 2,791,472 later offspring. Age was the time axis and robust standard errors were clustered by maternal identity. Adjustment set (e) (offspring sex and date of birth, maternal and paternal occupational and educational SEP, offspring birth order, and paternal age) was used.

	**Primary analysis hazard ratio per five years of mother's age at offspring's birth among:**		**Two-variable hazard ratio among later offspring per five years of mother's age at:**	
**Outcome**	**Oldest Offspring**	**Later offspring**	**Offspring's birth**	**Oldest offspring birth**
All-cause	0.96 (0.96, 0.97)	0.96 (0.95, 0.96)	1.02 (1.01, 1.03)	0.92 (0.91, 0.93)
Cardiovascular disease	0.95 (0.94, 0.97)	0.94 (0.92, 0.95)	1.03 (1.00, 1.05)	0.89 (0.87, 0.91)
Coronary heart disease	0.95 (0.93, 0.97)	0.94 (0.92, 0.96)	1.01 (0.98, 1.05)	0.90 (0.87, 0.94)
Aortic aneurysm	0.94 (0.88, 1.00)	0.90 (0.83, 0.96)	0.99 (0.88, 1.11)	0.87 (0.78, 0.98)
Stroke	0.95 (0.92, 0.98)	0.92 (0.89, 0.96)	1.01 (0.95, 1.07)	0.89 (0.84, 0.94)
Diabetes	0.93 (0.89, 0.98)	0.96 (0.91, 1.02)	1.08 (1.00, 1.17)	0.85 (0.78, 0.92)
Respiratory diseases	0.96 (0.93, 0.99)	0.97 (0.93, 1.01)	1.04 (0.97, 1.11)	0.91 (0.86, 0.97)
Kidney disease	0.93 (0.84, 1.03)	1.04 (0.91, 1.18)	1.03 (0.83, 1.28)	1.00 (0.82, 1.23)
Pancreatic disease	0.98 (0.86, 1.12)	0.92 (0.80, 1.06)	1.23 (1.02, 1.50)	0.65 (0.53, 0.80)
Liver cirrhosis	0.98 (0.92, 1.04)	1.00 (0.94, 1.07)	1.24 (1.13, 1.36)	0.74 (0.68, 0.82)
Alcohol dependence	0.97 (0.91, 1.04)	0.95 (0.89, 1.01)	1.04 (0.95, 1.14)	0.88 (0.80, 0.96)
Alzheimer's disease	1.01 (0.95, 1.08)	1.11 (1.00, 1.23)	1.13 (0.93, 1.38)	0.98 (0.81, 1.18)
Parkinson's disease	1.15 (1.01, 1.31)	0.95 (0.77, 1.17)	1.03 (0.69, 1.55)	0.91 (0.61, 1.37)
Multiple sclerosis	1.00 (0.90, 1.12)	1.02 (0.91, 1.14)	1.04 (0.89, 1.22)	0.97 (0.83, 1.14)
Motor neurone disease	0.95 (0.88, 1.04)	0.98 (0.88, 1.08)	1.01 (0.87, 1.17)	0.96 (0.82, 1.12)
External causes	0.89 (0.87, 0.91)	0.91 (0.89, 0.93)	0.95 (0.93, 0.98)	0.93 (0.90, 0.95)
Suicide	0.92 (0.89, 0.96)	0.93 (0.90, 0.96)	0.93 (0.90, 0.97)	0.99 (0.95, 1.03)
Any cancer	0.98 (0.97, 0.99)	0.97 (0.96, 0.99)	1.02 (1.00, 1.04)	0.94 (0.93, 0.96)
Malignant melanoma	1.07 (1.01, 1.14)	1.03 (0.96, 1.11)	1.08 (0.97, 1.19)	0.95 (0.85, 1.05)
Brain cancer	0.96 (0.92, 1.01)	0.97 (0.92, 1.03)	0.96 (0.89, 1.04)	1.01 (0.94, 1.10)
Oesophageal cancer	0.97 (0.90, 1.05)	0.97 (0.89, 1.06)	1.04 (0.91, 1.19)	0.91 (0.80, 1.04)
Thyroid cancer	0.74 (0.61, 0.90)	1.01 (0.80, 1.26)	1.25 (0.90, 1.76)	0.75 (0.52, 1.07)
Lymphatic cancer	1.02 (0.98, 1.06)	0.99 (0.95, 1.03)	1.07 (1.01, 1.15)	0.89 (0.84, 0.95)
Lung cancer	0.96 (0.94, 0.99)	0.92 (0.90, 0.95)	1.02 (0.98, 1.07)	0.87 (0.83, 0.92)
Breast cancer	1.01 (0.98, 1.05)	1.03 (0.99, 1.07)	1.04 (0.99, 1.10)	0.98 (0.93, 1.04)
Liver cancer	0.96 (0.90, 1.03)	0.97 (0.90, 1.05)	1.01 (0.90, 1.15)	0.94 (0.83, 1.07)
Gallbladder cancer	1.01 (0.94, 1.08)	1.01 (0.92, 1.10)	1.03 (0.90, 1.18)	0.97 (0.85, 1.11)
Stomach cancer	0.92 (0.86, 0.97)	0.89 (0.83, 0.96)	0.91 (0.81, 1.02)	0.98 (0.87, 1.09)
Kidney cancer	0.89 (0.84, 0.95)	0.98 (0.91, 1.06)	1.02 (0.90, 1.14)	0.96 (0.85, 1.08)
Pancreatic cancer	1.00 (0.95, 1.04)	0.94 (0.90, 0.99)	0.99 (0.92, 1.07)	0.93 (0.87, 1.00)
Colorectal cancer	0.95 (0.92, 0.98)	0.98 (0.94, 1.02)	1.02 (0.96, 1.08)	0.95 (0.90, 1.01)
Bladder cancer	1.05 (0.97, 1.13)	0.94 (0.85, 1.04)	0.95 (0.81, 1.12)	0.98 (0.84, 1.15)
Ovarian cancer	1.00 (0.95, 1.05)	0.99 (0.93, 1.05)	0.97 (0.89, 1.07)	1.02 (0.93, 1.11)
Uterine cancer	0.95 (0.89, 1.02)	0.96 (0.88, 1.04)	1.00 (0.88, 1.12)	0.95 (0.84, 1.08)
Prostate cancer	1.02 (0.97, 1.06)	1.05 (0.98, 1.12)	1.01 (0.90, 1.13)	1.04 (0.93, 1.16)
Testicular cancer	0.93 (0.66, 1.33)	1.38 (0.97, 1.97)	1.73 (1.11, 2.69)	0.69 (0.47, 1.03)

Table S7. Analyses of offspring mortality and paternal age, conducted separately for offspring who were the oldest child in their family and for others. Two-variable analyses of later children were also conducted with mutual adjustment for father's age at the birth of (i) the child in question, and (ii) his first child. Primary analyses used Cox proportional hazards regression of 2,454,558 oldest offspring and 2,749,875 later offspring. Age was the time axis and robust standard errors were clustered by paternal identity. Adjustment set (e) (offspring sex and date of birth, maternal and paternal occupational and educational SEP, offspring birth order, and maternal age) was used.

	**Primary analysis hazard ratio per five years of father's age at offspring's birth among:**		**Two-variable hazard ratio among later offspring per five years of father's age at:**	
**Outcome**	**Oldest Offspring**	**Later offspring**	**Offspring's birth**	**Oldest offspring birth**
All-cause	0.97 (0.96, 0.98)	0.98 (0.98, 0.99)	1.03 (1.02, 1.05)	0.94 (0.93, 0.95)
Cardiovascular disease	0.96 (0.95, 0.97)	0.98 (0.97, 1.00)	1.05 (1.02, 1.08)	0.93 (0.91, 0.95)
Coronary heart disease	0.96 (0.94, 0.97)	0.97 (0.95, 0.98)	1.01 (0.98, 1.05)	0.95 (0.92, 0.98)
Aortic aneurysm	0.97 (0.92, 1.02)	1.03 (0.96, 1.09)	1.13 (1.01, 1.26)	0.90 (0.81, 1.00)
Stroke	0.96 (0.93, 0.98)	0.99 (0.95, 1.02)	1.04 (0.98, 1.10)	0.94 (0.89, 1.00)
Diabetes	1.00 (0.96, 1.04)	0.97 (0.92, 1.02)	1.02 (0.94, 1.11)	0.94 (0.87, 1.02)
Respiratory diseases	0.95 (0.93, 0.98)	0.95 (0.92, 0.99)	1.00 (0.93, 1.06)	0.95 (0.89, 1.01)
Kidney disease	1.00 (0.92, 1.09)	0.89 (0.79, 1.00)	0.84 (0.69, 1.03)	1.06 (0.88, 1.29)
Pancreatic disease	0.95 (0.85, 1.06)	0.92 (0.81, 1.04)	1.25 (1.02, 1.53)	0.69 (0.56, 0.84)
Liver cirrhosis	0.89 (0.84, 0.93)	0.88 (0.83, 0.93)	1.08 (0.98, 1.18)	0.78 (0.72, 0.85)
Alcohol dependence	0.94 (0.89, 0.99)	0.96 (0.91, 1.02)	0.99 (0.91, 1.09)	0.96 (0.88, 1.05)
Alzheimer's disease	1.01 (0.96, 1.07)	0.96 (0.89, 1.05)	1.02 (0.85, 1.22)	0.94 (0.79, 1.13)
Parkinson's disease	0.90 (0.80, 1.01)	1.10 (0.91, 1.34)	1.12 (0.75, 1.69)	0.98 (0.67, 1.43)
Multiple sclerosis	1.06 (0.97, 1.16)	1.02 (0.92, 1.12)	0.95 (0.81, 1.12)	1.08 (0.92, 1.27)
Motor neurone disease	1.03 (0.96, 1.10)	0.99 (0.91, 1.08)	0.97 (0.83, 1.13)	1.03 (0.89, 1.19)
External causes	0.97 (0.95, 0.99)	0.99 (0.98, 1.01)	1.05 (1.03, 1.08)	0.93 (0.90, 0.95)
Suicide	0.98 (0.95, 1.01)	1.02 (1.00, 1.05)	1.06 (1.02, 1.10)	0.95 (0.92, 0.99)
Any cancer	0.98 (0.97, 0.99)	0.99 (0.98, 1.00)	1.02 (1.00, 1.04)	0.96 (0.95, 0.98)
Malignant melanoma	0.96 (0.91, 1.02)	0.93 (0.87, 0.99)	0.93 (0.84, 1.02)	1.00 (0.91, 1.11)
Brain cancer	1.03 (0.99, 1.08)	1.03 (0.98, 1.07)	1.01 (0.94, 1.09)	1.02 (0.95, 1.10)
Oesophageal cancer	1.00 (0.93, 1.06)	1.03 (0.95, 1.12)	1.15 (1.00, 1.32)	0.89 (0.79, 1.00)
Thyroid cancer	0.99 (0.83, 1.17)	1.03 (0.86, 1.24)	1.26 (0.93, 1.71)	0.79 (0.59, 1.07)
Lymphatic cancer	0.98 (0.95, 1.02)	1.00 (0.96, 1.04)	1.07 (1.00, 1.14)	0.92 (0.87, 0.98)
Lung cancer	0.96 (0.94, 0.98)	1.00 (0.97, 1.02)	1.07 (1.02, 1.13)	0.92 (0.88, 0.96)
Breast cancer	1.01 (0.98, 1.05)	1.02 (0.98, 1.05)	1.03 (0.97, 1.09)	0.99 (0.94, 1.04)
Liver cancer	0.91 (0.86, 0.96)	0.93 (0.87, 1.00)	0.98 (0.86, 1.11)	0.95 (0.84, 1.07)
Gallbladder cancer	0.96 (0.91, 1.02)	0.96 (0.89, 1.04)	0.97 (0.85, 1.11)	0.99 (0.87, 1.12)
Stomach cancer	1.02 (0.96, 1.08)	1.05 (0.98, 1.11)	1.08 (0.97, 1.21)	0.96 (0.87, 1.07)
Kidney cancer	0.99 (0.94, 1.05)	0.97 (0.91, 1.04)	1.05 (0.92, 1.18)	0.92 (0.82, 1.03)
Pancreatic cancer	0.98 (0.94, 1.01)	0.98 (0.94, 1.02)	0.98 (0.91, 1.06)	0.99 (0.93, 1.07)
Colorectal cancer	0.99 (0.96, 1.02)	1.01 (0.98, 1.05)	1.04 (0.98, 1.11)	0.97 (0.91, 1.03)
Bladder cancer	0.98 (0.91, 1.05)	1.00 (0.92, 1.10)	1.06 (0.90, 1.25)	0.94 (0.81, 1.10)
Ovarian cancer	1.00 (0.95, 1.04)	1.01 (0.96, 1.07)	0.99 (0.90, 1.08)	1.03 (0.95, 1.12)
Uterine cancer	1.02 (0.96, 1.08)	0.97 (0.90, 1.04)	0.95 (0.84, 1.07)	1.03 (0.92, 1.15)
Prostate cancer	0.99 (0.95, 1.03)	0.94 (0.89, 1.00)	0.96 (0.86, 1.08)	0.98 (0.88, 1.09)
Testicular cancer	1.05 (0.76, 1.44)	0.88 (0.65, 1.20)	1.04 (0.68, 1.58)	0.78 (0.51, 1.19)

Table S8. Primary analyses of offspring mortality and maternal age, conducted separately for offspring born up to 1969 (when parental age was decreasing) and for offspring born from 1970 (when parental age was increasing). Primary analyses used Cox proportional hazards regression of 3,494,225 offspring born before 1970 and 1,710,208 offspring born in 1970 or later. Age was the time axis and robust standard errors were clustered by maternal identity. Adjustment set (e) (offspring sex and date of birth, maternal and paternal occupational and educational SEP, offspring birth order, and paternal age) was used. Analyses of fewer than 20 deaths gave highly imprecise results and are omitted.

	**Deaths when DOB**		**Primary analysis hazard ratio per five years of mother's age at offspring's birth**	
**Outcome**	**< 1970**	**≥ 1970**	**DOB ≤ 1970**	**DOB ≥ 1970**
All-cause	280,292	13,178	0.96 (0.96, 0.97)	0.90 (0.87, 0.93)
Cardiovascular disease	75,445	907	0.95 (0.94, 0.96)	0.91 (0.81, 1.03)
Coronary heart disease	41,683	170	0.95 (0.93, 0.96)	0.93 (0.70, 1.24)
Aortic aneurysm	3,918	56	0.91 (0.87, 0.96)	0.97 (0.63, 1.52)
Stroke	14,553	139	0.94 (0.92, 0.96)	1.09 (0.83, 1.44)
Diabetes	5,811	145	0.94 (0.91, 0.98)	0.92 (0.70, 1.20)
Respiratory diseases	11,990	197	0.96 (0.93, 0.98)	1.18 (0.94, 1.48)
Kidney disease	1,253	18	0.95 (0.88, 1.03)	
Pancreatic disease	827	21	0.95 (0.86, 1.05)	0.87 (0.37, 2.04)
Liver cirrhosis	4,418	22	0.99 (0.95, 1.04)	1.11 (0.49, 2.54)
Alcohol dependence	4,072	29	0.96 (0.91, 1.00)	0.82 (0.50, 1.33)
Alzheimer's disease	2,873	0	1.04 (0.98, 1.10)	
Parkinson's disease	713	0	1.11 (0.99, 1.23)	
Multiple sclerosis	1,278	18	1.01 (0.94, 1.09)	
Motor neurone disease	1,893	21	0.96 (0.90, 1.03)	0.65 (0.41, 1.04)
External causes	30,617	8,041	0.92 (0.90, 0.94)	0.86 (0.82, 0.89)
Suicide	12,523	3,042	0.93 (0.91, 0.96)	0.94 (0.88, 1.00)
Any cancer	111,737	1,860	0.98 (0.97, 0.98)	0.97 (0.90, 1.05)
Malignant melanoma	3,337	135	1.05 (1.00, 1.10)	1.13 (0.89, 1.43)
Brain cancer	5,480	344	0.97 (0.93, 1.01)	0.90 (0.76, 1.07)
Oesophageal cancer	2,470	12	0.97 (0.92, 1.03)	
Thyroid cancer	315	8	0.85 (0.74, 0.99)	
Lymphatic cancer	9,112	296	1.00 (0.97, 1.03)	1.04 (0.84, 1.30)
Lung cancer	20,686	66	0.94 (0.93, 0.96)	1.13 (0.72, 1.77)
Breast cancer	10,730	157	1.02 (0.99, 1.05)	0.95 (0.74, 1.21)
Liver cancer	3,018	35	0.96 (0.91, 1.02)	1.31 (0.74, 2.32)
Gallbladder cancer	2,602	24	1.01 (0.96, 1.06)	0.94 (0.53, 1.66)
Stomach cancer	3,540	40	0.90 (0.86, 0.95)	1.14 (0.63, 2.05)
Kidney cancer	3,498	17	0.92 (0.88, 0.97)	
Pancreatic cancer	8,229	29	0.97 (0.94, 1.01)	0.83 (0.44, 1.58)
Colorectal cancer	12,161	164	0.96 (0.94, 0.98)	0.99 (0.78, 1.25)
Bladder cancer	2,164	9	1.01 (0.95, 1.07)	
Ovarian cancer	4,623	59	0.99 (0.95, 1.03)	1.27 (0.88, 1.85)
Uterine cancer	2,590	82	0.96 (0.91, 1.01)	0.94 (0.66, 1.34)
Prostate cancer	5,917	2	1.03 (0.99, 1.07)	
Testicular cancer	94	39	1.31 (0.98, 1.75)	1.05 (0.64, 1.73)

Table S9. Primary analyses of offspring mortality and paternal age, conducted separately for offspring born up to 1969 (when parental age was decreasing) and for offspring born from 1970 (when parental age was increasing). Primary analyses used Cox proportional hazards regression of 3,494,225 offspring born before 1970 and 1,710,208 offspring born in 1970 or later. Age was the time axis and robust standard errors were clustered by paternal identity. Adjustment set (e) (offspring sex and date of birth, maternal and paternal occupational and educational SEP, offspring birth order, and maternal age) was used. Analyses of fewer than 20 deaths gave highly imprecise results and are omitted.

	**Deaths when DOB**		**Primary analysis hazard ratio per five years of father's age at offspring's birth**	
**Outcome**	**< 1970**	**≥ 1970**	**DOB ≤ 1970**	**DOB ≥ 1970**
All-cause	280,292	13,178	0.97 (0.97, 0.98)	1.04 (1.02, 1.07)
Cardiovascular disease	75,445	907	0.97 (0.96, 0.98)	1.03 (0.95, 1.12)
Coronary heart disease	41,683	170	0.96 (0.95, 0.97)	1.01 (0.83, 1.23)
Aortic aneurysm	3,918	56	0.99 (0.95, 1.03)	1.06 (0.78, 1.43)
Stroke	14,553	139	0.96 (0.95, 0.98)	0.93 (0.75, 1.15)
Diabetes	5,811	145	0.98 (0.95, 1.02)	1.05 (0.84, 1.31)
Respiratory diseases	11,990	197	0.95 (0.93, 0.97)	1.12 (0.95, 1.33)
Kidney disease	1,253	18	0.96 (0.89, 1.03)	
Pancreatic disease	827	21	0.94 (0.86, 1.02)	0.86 (0.59, 1.23)
Liver cirrhosis	4,418	22	0.88 (0.84, 0.91)	1.22 (0.75, 1.97)
Alcohol dependence	4,072	29	0.95 (0.91, 0.99)	1.21 (0.84, 1.75)
Alzheimer's disease	2,873	0	1.00 (0.95, 1.05)	
Parkinson's disease	713	0	0.95 (0.86, 1.05)	
Multiple sclerosis	1,278	18	1.04 (0.97, 1.11)	
Motor neurone disease	1,893	21	1.01 (0.96, 1.07)	1.33 (0.93, 1.91)
External causes	30,617	8,041	0.98 (0.96, 0.99)	1.04 (1.01, 1.07)
Suicide	12,523	3,042	1.00 (0.98, 1.02)	1.05 (1.00, 1.10)
Any cancer	111,737	1,860	0.99 (0.98, 0.99)	1.01 (0.95, 1.07)
Malignant melanoma	3,337	135	0.95 (0.91, 1.00)	0.90 (0.73, 1.12)
Brain cancer	5,480	344	1.02 (0.99, 1.06)	1.15 (1.01, 1.31)
Oesophageal cancer	2,470	12	1.01 (0.96, 1.06)	
Thyroid cancer	315	8	0.99 (0.87, 1.13)	
Lymphatic cancer	9,112	296	0.99 (0.97, 1.02)	0.99 (0.84, 1.17)
Lung cancer	20,686	66	0.97 (0.96, 0.99)	0.74 (0.48, 1.13)
Breast cancer	10,730	157	1.02 (0.99, 1.04)	1.03 (0.85, 1.24)
Liver cancer	3,018	35	0.92 (0.87, 0.96)	0.90 (0.56, 1.45)
Gallbladder cancer	2,602	24	0.97 (0.92, 1.02)	0.80 (0.51, 1.23)
Stomach cancer	3,540	40	1.03 (0.99, 1.08)	0.87 (0.54, 1.41)
Kidney cancer	3,498	17	0.99 (0.95, 1.03)	
Pancreatic cancer	8,229	29	0.97 (0.95, 1.00)	0.94 (0.64, 1.40)
Colorectal cancer	12,161	164	1.00 (0.98, 1.02)	0.96 (0.79, 1.17)
Bladder cancer	2,164	9	0.98 (0.93, 1.04)	
Ovarian cancer	4,623	59	1.00 (0.97, 1.04)	1.02 (0.75, 1.37)
Uterine cancer	2,590	82	1.00 (0.95, 1.05)	0.84 (0.67, 1.05)
Prostate cancer	5,917	2	0.97 (0.94, 1.00)	
Testicular cancer	94	39	0.83 (0.66, 1.05)	1.21 (0.81, 1.82)

Table S10. Sibling comparison analyses of offspring mortality and maternal age, conducted separately for offspring born up to 1969 (when parental age was decreasing) and for offspring born from 1970 (when parental age was increasing). Sibling-comparison analyses used Cox regression stratified by maternal identity and were restricted to offspring in families with discordant outcomes (maximum N=421,002 for all-cause mortality). All family-level confounding was intrinsically adjusted for and adjustment for offspring DOB or paternal age were not possible. Explicit adjustment was therefore limited to offspring sex and birth order. Analyses of fewer than 20 deaths gave highly imprecise results and are omitted.

	**Deaths when DOB**		**Sibling comparison hazard ratio per five years of mother's age at offspring's birth**	
**Outcome**	**< 1970**	**≥ 1970**	**DOB ≤ 1970**	**DOB ≥ 1970**
All-cause	179,867	7,963	0.94 (0.93, 0.96)	0.88 (0.80, 0.96)
Cardiovascular disease	44,934	512	0.88 (0.85, 0.91)	1.00 (0.68, 1.48)
Coronary heart disease	25,062	82	0.83 (0.80, 0.87)	0.12 (0.03, 0.46)
Aortic aneurysm	2,298	31	0.85 (0.73, 0.99)	2.34 (0.29, 18.97)
Stroke	8,258	76	0.85 (0.78, 0.91)	1.08 (0.40, 2.90)
Diabetes	3,308	82	1.01 (0.89, 1.13)	1.50 (0.62, 3.63)
Respiratory diseases	6,730	109	0.90 (0.83, 0.98)	1.15 (0.49, 2.70)
Kidney disease	673	10	0.89 (0.68, 1.17)	
Pancreatic disease	500	12	1.06 (0.80, 1.39)	
Liver cirrhosis	2,694	11	0.95 (0.84, 1.08)	
Alcohol dependence	2,790	15	0.82 (0.72, 0.93)	
Alzheimer's disease	1,244	0	1.21 (0.97, 1.51)	
Parkinson's disease	290	0	1.37 (0.81, 2.32)	
Multiple sclerosis	778	9	0.95 (0.75, 1.20)	
Motor neurone disease	1,151	6	0.96 (0.78, 1.18)	
External causes	20,580	5,022	0.90 (0.86, 0.95)	0.90 (0.80, 1.00)
Suicide	8,623	1,877	0.92 (0.86, 0.99)	0.99 (0.83, 1.18)
Any cancer	70,247	1,033	0.95 (0.92, 0.97)	0.75 (0.57, 0.98)
Malignant melanoma	2,050	77	1.08 (0.93, 1.25)	0.77 (0.24, 2.42)
Brain cancer	3,679	209	0.98 (0.88, 1.09)	0.57 (0.28, 1.16)
Oesophageal cancer	1,448	3	1.08 (0.90, 1.30)	
Thyroid cancer	202	3	1.38 (0.87, 2.19)	
Lymphatic cancer	5,512	180	0.96 (0.87, 1.05)	0.87 (0.47, 1.61)
Lung cancer	12,952	26	0.94 (0.89, 1.00)	0.22 (0.04, 1.28)
Breast cancer	4,500	34	0.90 (0.82, 1.00)	0.81 (0.08, 7.73)
Liver cancer	1,788	19	0.94 (0.80, 1.11)	
Gallbladder cancer	1,531	11	1.01 (0.86, 1.20)	
Stomach cancer	2,318	18	0.81 (0.70, 0.93)	
Kidney cancer	2,131	9	0.75 (0.65, 0.87)	
Pancreatic cancer	4,962	19	0.95 (0.86, 1.04)	
Colorectal cancer	7,209	83	0.99 (0.91, 1.08)	0.62 (0.18, 2.12)
Bladder cancer	1,208	6	0.98 (0.79, 1.21)	
Ovarian cancer	1,815	17	1.01 (0.86, 1.18)	
Uterine cancer	970	19	1.08 (0.88, 1.32)	
Prostate cancer	1,935	1	0.99 (0.83, 1.18)	
Testicular cancer	21	11	3.97 (0.62, 25.57)	

Table S11. Sibling comparison analyses of offspring mortality and paternal age, conducted separately for offspring born up to 1969 (when parental age was decreasing) and for offspring born from 1970 (when parental age was increasing). Sibling-comparison analyses used Cox regression stratified by paternal identity and were restricted to offspring in families with discordant outcomes (maximum N=423,068 for all-cause mortality). All family-level confounding was intrinsically adjusted for and adjustment for offspring DOB or maternal age were not possible. Explicit adjustment was therefore limited to offspring sex and birth order. Analyses of fewer than 20 deaths gave highly imprecise results and are omitted.

	**Deaths when DOB**		**Sibling comparison hazard ratio per five years of father's age at offspring's birth**	
**Outcome**	**< 1970**	**≥ 1970**	**DOB ≤ 1970**	**DOB ≥ 1970**
All-cause	179,863	7,850	0.93 (0.91, 0.94)	0.95 (0.89, 1.01)
Cardiovascular disease	44,895	511	0.87 (0.84, 0.89)	0.96 (0.72, 1.28)
Coronary heart disease	25,029	78	0.82 (0.79, 0.86)	0.38 (0.16, 0.90)
Aortic aneurysm	2,302	33	0.86 (0.75, 0.99)	1.20 (0.24, 6.01)
Stroke	8,225	76	0.82 (0.77, 0.88)	0.59 (0.28, 1.23)
Diabetes	3,307	80	0.96 (0.86, 1.06)	1.23 (0.65, 2.34)
Respiratory diseases	6,721	104	0.86 (0.80, 0.93)	0.78 (0.45, 1.37)
Kidney disease	670	10	0.81 (0.62, 1.05)	
Pancreatic disease	501	11	0.99 (0.78, 1.25)	
Liver cirrhosis	2,704	11	0.91 (0.82, 1.02)	
Alcohol dependence	2,787	15	0.74 (0.66, 0.83)	
Alzheimer's disease	1,258	0	1.15 (0.93, 1.41)	
Parkinson's disease	290	0	1.29 (0.76, 2.20)	
Multiple sclerosis	786	9	0.94 (0.76, 1.16)	
Motor neurone disease	1,150	6	0.88 (0.74, 1.06)	
External causes	20,607	4,957	0.92 (0.89, 0.96)	0.98 (0.91, 1.06)
Suicide	8,618	1,861	0.94 (0.89, 1.00)	1.08 (0.96, 1.22)
Any cancer	70,313	1,023	0.94 (0.92, 0.96)	0.87 (0.71, 1.07)
Malignant melanoma	2,084	74	1.09 (0.95, 1.24)	1.20 (0.53, 2.73)
Brain cancer	3,686	209	0.96 (0.87, 1.06)	0.94 (0.59, 1.50)
Oesophageal cancer	1,457	3	1.10 (0.93, 1.30)	
Thyroid cancer	199	3	1.29 (0.83, 2.01)	
Lymphatic cancer	5,515	176	0.96 (0.89, 1.05)	0.78 (0.49, 1.24)
Lung cancer	12,897	24	0.91 (0.86, 0.96)	0.25 (0.04, 1.46)
Breast cancer	4,540	34	0.93 (0.85, 1.01)	3.80 (0.35, 41.51)
Liver cancer	1,793	19	0.91 (0.79, 1.05)	
Gallbladder cancer	1,544	10	1.02 (0.87, 1.18)	
Stomach cancer	2,313	20	0.87 (0.77, 0.98)	0.61 (0.19, 2.03)
Kidney cancer	2,142	9	0.78 (0.68, 0.89)	
Pancreatic cancer	4,950	19	0.92 (0.84, 1.00)	
Colorectal cancer	7,207	85	0.95 (0.88, 1.02)	1.02 (0.42, 2.46)
Bladder cancer	1,212	5	0.99 (0.82, 1.21)	
Ovarian cancer	1,819	14	0.95 (0.82, 1.10)	
Uterine cancer	975	20	1.04 (0.86, 1.26)	0.93 (0.33, 2.63)
Prostate cancer	1,942	1	1.00 (0.85, 1.19)	
Testicular cancer	21	10	5.24 (0.84, 32.71)	

Table S12. Primary analyses of son's outcomes and maternal age, with and without additional adjustment for maternal survival to the offspring's 35th birthday. Primary analyses used Cox proportional hazards regression of 2,636,028 offspring for which maternal survival to the offspring's 35th birthday could be established. Age was the time axis and robust standard errors were clustered by maternal identity. Adjustment set (e) (offspring sex and date of birth, maternal and paternal occupational and educational SEP, offspring birth order, and paternal age) was used. Analyses of fewer than 20 deaths gave highly imprecise results and are omitted.

		**Primary analysis hazard ratio per five years of mother's age at offspring's birth**	
**Outcome**	**Deaths**	**not adjusted for lifespan overlap**	**adjusted for lifespan overlap**
All-cause	58,923	0.94 (0.93, 0.95)	0.93 (0.92, 0.94)
Cardiovascular disease	9,834	0.94 (0.91, 0.97)	0.92 (0.90, 0.95)
Coronary heart disease	4,508	0.94 (0.91, 0.98)	0.93 (0.89, 0.97)
Aortic aneurysm	464	0.98 (0.85, 1.13)	0.97 (0.84, 1.11)
Stroke	1,906	0.92 (0.86, 0.98)	0.90 (0.85, 0.96)
Diabetes	1,140	0.97 (0.89, 1.06)	0.96 (0.88, 1.04)
Respiratory diseases	1,365	0.99 (0.91, 1.07)	0.97 (0.89, 1.05)
Kidney disease	179	1.25 (1.01, 1.55)	1.23 (1.00, 1.52)
Pancreatic disease	230	1.01 (0.84, 1.21)	0.99 (0.83, 1.19)
Liver cirrhosis	898	1.01 (0.91, 1.11)	0.99 (0.90, 1.10)
Alcohol dependence	1,049	0.90 (0.82, 0.99)	0.88 (0.80, 0.97)
Alzheimer's disease	59	1.64 (1.11, 2.43)	1.63 (1.10, 2.42)
Parkinson's disease	11		
Multiple sclerosis	358	1.05 (0.92, 1.21)	1.04 (0.90, 1.19)
Motor neurone disease	289	1.06 (0.90, 1.25)	1.05 (0.89, 1.23)
External causes	18,567	0.87 (0.85, 0.89)	0.86 (0.84, 0.88)
Suicide	8,027	0.91 (0.88, 0.94)	0.89 (0.86, 0.92)
Any cancer	17,162	0.98 (0.95, 1.00)	0.97 (0.95, 0.99)
Malignant melanoma	960	1.06 (0.98, 1.16)	1.06 (0.97, 1.15)
Brain cancer	1,734	0.96 (0.89, 1.02)	0.95 (0.89, 1.02)
Oesophageal cancer	315	1.00 (0.85, 1.18)	0.99 (0.84, 1.17)
Thyroid cancer	40	0.76 (0.50, 1.17)	0.75 (0.49, 1.15)
Lymphatic cancer	1,538	0.95 (0.89, 1.02)	0.95 (0.88, 1.02)
Lung cancer	1,950	0.89 (0.83, 0.95)	0.88 (0.83, 0.95)
Breast cancer	2,643	0.99 (0.94, 1.04)	0.98 (0.93, 1.03)
Liver cancer	458	1.00 (0.86, 1.15)	0.98 (0.85, 1.13)
Gallbladder cancer	323	1.02 (0.89, 1.17)	1.02 (0.89, 1.17)
Stomach cancer	548	0.88 (0.78, 0.99)	0.87 (0.77, 0.98)
Kidney cancer	339	0.92 (0.78, 1.08)	0.91 (0.78, 1.07)
Pancreatic cancer	904	1.01 (0.92, 1.11)	1.00 (0.91, 1.10)
Colorectal cancer	1,746	1.05 (0.98, 1.13)	1.04 (0.98, 1.12)
Bladder cancer	190	1.09 (0.89, 1.34)	1.09 (0.88, 1.35)
Ovarian cancer	746	1.01 (0.91, 1.12)	1.00 (0.90, 1.10)
Uterine cancer	620	0.96 (0.86, 1.08)	0.96 (0.85, 1.08)
Prostate cancer	178	1.00 (0.80, 1.25)	1.00 (0.80, 1.25)
Testicular cancer	75	1.15 (0.81, 1.63)	1.12 (0.80, 1.59)

Table S13. Primary analyses of son's outcomes and paternal age, with and without additional adjustment for paternal survival to the offspring's 35th birthday. Primary analyses used Cox proportional hazards regression of 2,635,996 offspring for which paternal survival to the offspring's 35th birthday could be established. Age was the time axis and robust standard errors were clustered by paternal identity. Adjustment set (e) (offspring sex and date of birth, maternal and paternal occupational and educational SEP, offspring birth order, and maternal age) was used. Analyses of fewer than 20 deaths gave highly imprecise results and are omitted.

		**Primary analysis hazard ratio per five years of father's age at offspring's birth**	
**Outcome**	**Deaths**	**not adjusted for lifespan overlap**	**adjusted for lifespan overlap**
All-cause	58,922	0.98 (0.97, 0.99)	0.96 (0.95, 0.97)
Cardiovascular disease	9,834	1.00 (0.98, 1.02)	0.97 (0.95, 0.99)
Coronary heart disease	4,508	0.99 (0.95, 1.03)	0.96 (0.92, 0.99)
Aortic aneurysm	464	1.03 (0.91, 1.16)	0.99 (0.88, 1.12)
Stroke	1,906	0.97 (0.92, 1.02)	0.95 (0.90, 1.00)
Diabetes	1,140	0.98 (0.91, 1.06)	0.95 (0.89, 1.03)
Respiratory diseases	1,365	0.96 (0.90, 1.03)	0.94 (0.87, 1.00)
Kidney disease	179	0.72 (0.60, 0.87)	0.72 (0.60, 0.86)
Pancreatic disease	230	0.97 (0.84, 1.12)	0.94 (0.81, 1.09)
Liver cirrhosis	898	0.83 (0.76, 0.91)	0.81 (0.74, 0.88)
Alcohol dependence	1,048	0.88 (0.82, 0.96)	0.86 (0.79, 0.93)
Alzheimer's disease	59	0.90 (0.67, 1.22)	0.94 (0.69, 1.27)
Parkinson's disease	11		
Multiple sclerosis	358	1.00 (0.90, 1.13)	0.99 (0.88, 1.11)
Motor neurone disease	289	0.96 (0.84, 1.11)	0.97 (0.84, 1.12)
External causes	18,567	0.99 (0.98, 1.01)	0.97 (0.95, 0.98)
Suicide	8,027	1.02 (0.99, 1.05)	0.99 (0.96, 1.02)
Any cancer	17,162	0.99 (0.97, 1.01)	0.98 (0.96, 1.00)
Malignant melanoma	960	0.95 (0.88, 1.02)	0.93 (0.86, 1.00)
Brain cancer	1,734	1.04 (0.98, 1.10)	1.02 (0.96, 1.08)
Oesophageal cancer	315	1.07 (0.92, 1.23)	1.06 (0.92, 1.23)
Thyroid cancer	40	0.88 (0.63, 1.23)	0.84 (0.60, 1.18)
Lymphatic cancer	1,538	1.05 (0.98, 1.11)	1.04 (0.98, 1.11)
Lung cancer	1,950	0.95 (0.90, 1.00)	0.93 (0.88, 0.98)
Breast cancer	2,643	1.06 (1.01, 1.11)	1.05 (1.01, 1.10)
Liver cancer	458	0.82 (0.73, 0.93)	0.80 (0.71, 0.91)
Gallbladder cancer	323	0.98 (0.86, 1.11)	0.97 (0.86, 1.10)
Stomach cancer	548	1.06 (0.96, 1.18)	1.04 (0.94, 1.16)
Kidney cancer	339	0.98 (0.86, 1.12)	0.97 (0.85, 1.10)
Pancreatic cancer	904	0.91 (0.84, 0.98)	0.90 (0.83, 0.97)
Colorectal cancer	1,746	0.97 (0.91, 1.02)	0.96 (0.90, 1.02)
Bladder cancer	190	0.84 (0.70, 1.02)	0.84 (0.70, 1.02)
Ovarian cancer	746	0.97 (0.89, 1.05)	0.96 (0.88, 1.04)
Uterine cancer	620	0.97 (0.88, 1.07)	0.97 (0.88, 1.08)
Prostate cancer	178	0.90 (0.73, 1.11)	0.90 (0.73, 1.11)
Testicular cancer	75	0.90 (0.69, 1.17)	0.92 (0.70, 1.19)

Table S14. Primary analyses of offspring mortality and maternal age, with and without additional adjustment for offspring birth weight and birth length. Primary analyses used Cox proportional hazards regression of 1,372,263 offspring born in 1973 or later with data on birth weight and length. Age was the time axis and robust standard errors were clustered by maternal identity. Adjustment set (e) (offspring sex and date of birth, maternal and paternal occupational and educational SEP, offspring birth order, and paternal age) was used. Analyses of fewer than 20 deaths gave highly imprecise results and are omitted.

		Primary analysis hazard ratio per five years of mother's age at offspring's birth	
**Outcome**	**Deaths**	**without birth weight & length**	**with birth weight & length**
All-cause	9,469	0.89 (0.86, 0.92)	0.89 (0.86, 0.92)
Cardiovascular disease	578	0.87 (0.75, 1.01)	0.87 (0.75, 1.01)
Coronary heart disease	92	0.80 (0.53, 1.21)	0.80 (0.53, 1.22)
Aortic aneurysm	30	1.13 (0.64, 2.00)	1.14 (0.64, 2.03)
Stroke	87	0.87 (0.61, 1.26)	0.87 (0.61, 1.25)
Diabetes	98	0.97 (0.70, 1.34)	0.97 (0.70, 1.33)
Respiratory diseases	149	1.14 (0.87, 1.49)	1.13 (0.86, 1.47)
Kidney disease	9		
Pancreatic disease	14		
Liver cirrhosis	12		
Alcohol dependence	10		
Alzheimer's disease	0		
Parkinson's disease	0		
Multiple sclerosis	10		
Motor neurone disease	6		
External causes	6,089	0.85 (0.81, 0.89)	0.85 (0.81, 0.89)
Suicide	2,267	0.91 (0.84, 0.99)	0.91 (0.84, 0.99)
Any cancer	1,172	0.99 (0.90, 1.09)	0.99 (0.90, 1.09)
Malignant melanoma	88	1.14 (0.84, 1.54)	1.14 (0.84, 1.55)
Brain cancer	232	0.98 (0.80, 1.21)	0.99 (0.80, 1.21)
Oesophageal cancer	2		
Thyroid cancer	5		
Lymphatic cancer	213	1.14 (0.89, 1.46)	1.14 (0.89, 1.46)
Lung cancer	33	1.21 (0.68, 2.16)	1.21 (0.67, 2.16)
Breast cancer	83	0.93 (0.67, 1.29)	0.93 (0.67, 1.29)
Liver cancer	24	1.27 (0.62, 2.60)	1.26 (0.62, 2.60)
Gallbladder cancer	15		
Stomach cancer	23	0.82 (0.49, 1.36)	0.81 (0.49, 1.35)
Kidney cancer	10		
Pancreatic cancer	16		
Colorectal cancer	85	0.96 (0.67, 1.38)	0.96 (0.67, 1.37)
Bladder cancer	4		
Ovarian cancer	36	1.27 (0.73, 2.19)	1.25 (0.72, 2.15)
Uterine cancer	46	1.00 (0.61, 1.64)	1.00 (0.61, 1.64)
Prostate cancer	2		
Testicular cancer	23	0.95 (0.50, 1.83)	0.95 (0.50, 1.81)

Table S15. Primary analyses of offspring mortality and paternal age, with and without additional adjustment for offspring birth weight and birth length. Primary analyses used Cox proportional hazards regression of 1,372,263 offspring born in 1973 or later with data on birth weight and length. Age was the time axis and robust standard errors were clustered by paternal identity. Adjustment set (e) (offspring sex and date of birth, maternal and paternal occupational and educational SEP, offspring birth order, and maternal age) was used. Analyses of fewer than 20 deaths gave highly imprecise results and are omitted.

		**Primary analysis hazard ratio per five years of father's age at offspring's birth**	
**Outcome**	**Deaths**	**without birth weight & length**	**with birth weight & length**
All-cause	9,469	1.06 (1.03, 1.09)	1.06 (1.03, 1.09)
Cardiovascular disease	578	0.98 (0.88, 1.09)	0.98 (0.88, 1.09)
Coronary heart disease	92	1.00 (0.78, 1.29)	1.00 (0.78, 1.29)
Aortic aneurysm	30	0.80 (0.47, 1.34)	0.79 (0.47, 1.34)
Stroke	87	0.94 (0.72, 1.21)	0.94 (0.72, 1.21)
Diabetes	98	1.05 (0.85, 1.28)	1.04 (0.86, 1.27)
Respiratory diseases	149	1.12 (0.90, 1.39)	1.12 (0.90, 1.38)
Kidney disease	9		
Pancreatic disease	14		
Liver cirrhosis	12		
Alcohol dependence	10		
Alzheimer's disease	0		
Parkinson's disease	0		
Multiple sclerosis	10		
Motor neurone disease	6		
External causes	6,089	1.05 (1.01, 1.09)	1.05 (1.01, 1.09)
Suicide	2,267	1.06 (1.01, 1.12)	1.06 (1.01, 1.12)
Any cancer	1,172	1.04 (0.97, 1.12)	1.04 (0.97, 1.12)
Malignant melanoma	88	0.98 (0.77, 1.25)	0.98 (0.77, 1.25)
Brain cancer	232	1.14 (0.98, 1.33)	1.14 (0.98, 1.33)
Oesophageal cancer	2		
Thyroid cancer	5		
Lymphatic cancer	213	0.94 (0.78, 1.12)	0.94 (0.78, 1.12)
Lung cancer	33	0.71 (0.48, 1.06)	0.71 (0.48, 1.06)
Breast cancer	83	0.93 (0.71, 1.22)	0.93 (0.71, 1.22)
Liver cancer	24	1.03 (0.59, 1.79)	1.03 (0.60, 1.80)
Gallbladder cancer	15		
Stomach cancer	23	1.25 (0.89, 1.76)	1.25 (0.89, 1.76)
Kidney cancer	10		
Pancreatic cancer	16		
Colorectal cancer	85	1.09 (0.86, 1.37)	1.09 (0.86, 1.38)
Bladder cancer	4		
Ovarian cancer	36	1.09 (0.72, 1.67)	1.10 (0.72, 1.67)
Uterine cancer	46	0.83 (0.60, 1.16)	0.83 (0.60, 1.15)
Prostate cancer	2		
Testicular cancer	23	1.55 (1.04, 2.32)	1.56 (1.04, 2.33)

Table S16. Associations between offspring mortality and maternal age from primary and sibling comparison analyses, including the potentially biased subset of 39,558 offspring who died between 1961 and 30th June 1991. Primary analyses used Cox proportional hazards regression of 2,682,440 male and 2,561,551 female offspring. Age was the time axis and robust standard errors were clustered by maternal identity. Adjustment set (e) (offspring sex and date of birth (DOB), maternal and paternal occupational and educational SEP, offspring birth order, and paternal age) was used. The secular trend per five years of offspring DOB was assessed using a similar model but without maternal age. The primary analysis was repeated with adjustment for maternal, not offspring, DOB to account for confounding, but not mediation, by secular trends. To examine whether the restricted dataset used for sibling-comparison analyses was representative of the main dataset, the primary analysis was repeated on this subset. Finally, the sibling-comparison analysis used Cox regression stratified by maternal identity and was restricted to offspring in families with discordant outcomes. All family-level confounding was intrinsically adjusted for and adjustment for offspring DOB or paternal age were not possible. Explicit adjustment was therefore limited to offspring sex and birth order.

				**Hazard ratio per five years of mother's age at offspring's birth (or per five years of offspring's DOB for secular trend)**				
**Outcome**	**Deaths (main data)**	**Deaths (restricted data)**	**N (restricted data)**	**Primary analysis (main data)**	**Secular trend (main data)**	**Primary analysis plus mediation (main data)**	**Primary analysis (restricted data)**	**Sibling-comparison analysis (restricted data)**
All-cause	319,629	222,039	534,100	0.96 (0.95, 0.96)	1.07 (1.07, 1.08)	1.03 (1.02, 1.03)	1.06 (1.06, 1.06)	1.05 (1.03, 1.06)
Cardiovascular disease	78,671	49,090	129,998	0.94 (0.93, 0.95)	1.04 (1.04, 1.05)	0.99 (0.97, 1.00)	1.05 (1.04, 1.06)	1.03 (1.00, 1.06)
Coronary heart disease	42,601	26,561	72,737	0.94 (0.93, 0.95)	1.00 (0.99, 1.01)	0.95 (0.93, 0.97)	1.04 (1.03, 1.05)	0.98 (0.94, 1.02)
Aortic aneurysm	4,057	2,457	6,792	0.91 (0.87, 0.95)	1.05 (1.01, 1.08)	0.96 (0.91, 1.01)	1.05 (1.02, 1.09)	1.05 (0.91, 1.21)
Stroke	15,323	9,162	25,849	0.93 (0.91, 0.96)	1.01 (0.99, 1.02)	0.94 (0.92, 0.97)	1.06 (1.05, 1.08)	0.98 (0.91, 1.05)
Diabetes	6,503	3,988	11,193	0.94 (0.91, 0.98)	1.04 (1.02, 1.06)	0.98 (0.94, 1.02)	1.09 (1.06, 1.11)	1.04 (0.94, 1.15)
Respiratory diseases	12,887	7,646	21,187	0.96 (0.93, 0.98)	1.02 (1.00, 1.04)	0.98 (0.95, 1.01)	1.08 (1.06, 1.09)	1.05 (0.98, 1.13)
Kidney disease	1,529	898	2,506	0.98 (0.91, 1.06)	0.81 (0.77, 0.85)	0.80 (0.73, 0.87)	1.04 (1.00, 1.08)	0.80 (0.65, 0.99)
Pancreatic disease	928	612	1,798	0.96 (0.87, 1.05)	1.04 (0.99, 1.10)	1.00 (0.90, 1.11)	1.09 (1.01, 1.17)	1.20 (0.96, 1.52)
Liver cirrhosis	4,679	3,023	8,488	1.00 (0.96, 1.04)	1.06 (1.03, 1.09)	1.06 (1.01, 1.11)	1.06 (1.04, 1.09)	1.07 (0.95, 1.19)
Alcohol dependence	4,374	3,239	9,832	0.96 (0.92, 1.00)	1.02 (0.99, 1.04)	0.97 (0.93, 1.02)	1.09 (1.05, 1.12)	1.02 (0.91, 1.13)
Alzheimer's disease	2,876	1,250	3,200	1.03 (0.98, 1.09)	1.09 (1.02, 1.17)	1.12 (1.03, 1.21)	1.06 (1.02, 1.10)	1.23 (0.99, 1.53)
Parkinson's disease	713	291	739	1.10 (0.99, 1.23)	1.24 (1.07, 1.43)	1.33 (1.12, 1.58)	1.07 (0.97, 1.18)[a]	1.47 (0.88, 2.45)
Multiple sclerosis	1,395	918	2,544	1.03 (0.95, 1.10)	1.10 (1.05, 1.15)	1.13 (1.04, 1.22)	1.07 (1.02, 1.11)	1.12 (0.92, 1.38)
Motor neurone disease	1,964	1,228	3,427	0.97 (0.91, 1.03)	1.17 (1.12, 1.23)	1.14 (1.05, 1.23)	1.06 (1.02, 1.11)	1.05 (0.87, 1.26)
External causes	52,759	41,295	119,379	0.89 (0.88, 0.90)	1.08 (1.08, 1.09)	0.96 (0.95, 0.97)	1.10 (1.09, 1.11)	0.97 (0.94, 1.00)
Suicide	20,564	16,356	48,117	0.90 (0.89, 0.92)	1.10 (1.10, 1.11)	1.00 (0.97, 1.02)	1.11 (1.09, 1.12)	1.00 (0.96, 1.05)
Any cancer	118,305	78,052	204,649	0.97 (0.97, 0.98)	1.06 (1.05, 1.06)	1.03 (1.02, 1.04)	1.06 (1.06, 1.07)	1.05 (1.02, 1.07)
Malignant melanoma	3,733	2,466	6,807	1.05 (1.00, 1.09)	1.16 (1.13, 1.20)	1.22 (1.16, 1.28)	1.11 (1.08, 1.15)	1.16 (1.02, 1.32)
Brain cancer	6,392	4,644	13,174	0.97 (0.93, 1.00)	1.10 (1.08, 1.12)	1.06 (1.02, 1.11)	1.08 (1.06, 1.11)	1.12 (1.02, 1.23)
Oesophageal cancer	2,510	1,502	4,159	0.97 (0.92, 1.03)	1.14 (1.09, 1.19)	1.11 (1.04, 1.19)	1.06 (1.02, 1.11)	1.17 (0.98, 1.40)
Thyroid cancer	338	223	622	0.84 (0.73, 0.97)	1.08 (0.96, 1.22)	0.92 (0.77, 1.10)	1.11 (1.00, 1.23)	1.40 (0.92, 2.13)
Lymphatic cancer	10,517	6,928	19,585	1.00 (0.97, 1.02)	0.96 (0.94, 0.97)	0.95 (0.92, 0.98)	1.07 (1.06, 1.09)	1.04 (0.96, 1.12)

Lung cancer	21,053	13,524	37,615	0.94 (0.92, 0.96)	1.03 (1.02, 1.05)	0.98 (0.96, 1.00)	1.06 (1.05, 1.08)	1.02 (0.97, 1.08)
Breast cancer	11,359	5,135	12,358	1.02 (0.99, 1.04)	1.10 (1.08, 1.11)	1.12 (1.08, 1.15)	1.04 (1.02, 1.06)	1.05 (0.96, 1.14)
Liver cancer	3,133	1,916	5,387	0.97 (0.92, 1.02)	1.15 (1.11, 1.19)	1.11 (1.05, 1.18)	1.08 (1.05, 1.12)	1.03 (0.89, 1.19)
Gallbladder cancer	2,669	1,628	4,768	1.00 (0.95, 1.05)	1.10 (1.06, 1.15)	1.11 (1.04, 1.18)	1.07 (1.03, 1.11)	1.17 (1.00, 1.37)
Stomach cancer	3,730	2,527	7,488	0.90 (0.86, 0.95)	1.00 (0.97, 1.03)	0.91 (0.86, 0.96)	1.06 (1.03, 1.09)	0.92 (0.81, 1.05)
Kidney cancer	3,602	2,268	6,578	0.92 (0.87, 0.96)	0.95 (0.91, 0.98)	0.88 (0.83, 0.93)	1.04 (1.01, 1.07)	0.90 (0.79, 1.03)
Pancreatic cancer	8,386	5,187	14,608	0.97 (0.94, 1.00)	1.08 (1.05, 1.11)	1.05 (1.01, 1.09)	1.07 (1.05, 1.09)	1.01 (0.92, 1.10)
Colorectal cancer	12,637	7,769	21,486	0.96 (0.93, 0.98)	1.09 (1.07, 1.11)	1.05 (1.02, 1.08)	1.08 (1.06, 1.10)	1.11 (1.03, 1.20)
Bladder cancer	2,189	1,243	3,473	1.01 (0.95, 1.07)	1.06 (1.01, 1.12)	1.07 (0.99, 1.15)	1.06 (1.02, 1.10)	1.08 (0.88, 1.32)
Ovarian cancer	4,838	2,029	4,838	0.99 (0.95, 1.02)	1.07 (1.04, 1.10)	1.05 (1.01, 1.10)	1.06 (1.02, 1.09)	1.12 (0.97, 1.30)
Uterine cancer	2,857	1,138	2,807	0.96 (0.91, 1.01)	1.14 (1.10, 1.17)	1.09 (1.03, 1.16)	1.04 (0.99, 1.09)	1.06 (0.88, 1.27)
Prostate cancer	5,930	1,963	4,620	1.02 (0.99, 1.06)	0.93 (0.89, 0.96)	0.94 (0.90, 0.99)	0.99 (0.96, 1.02)	1.03 (0.87, 1.23)
Testicular cancer	280	134	320	1.00 (0.84, 1.18)	0.91 (0.85, 0.97)	0.91 (0.76, 1.09)	1.09 (0.91, 1.31)	1.11 (0.73, 1.67)

[a]This analysis failed for sons and daughters combined. The hazard ratio was estimated by meta-analysing separate results for sons and daughters.

Table S17. Associations between offspring mortality and paternal age from primary and sibling comparison analyses, including the potentially biased subset of 39,558 offspring who died between 1961 and 30th June 1991. Primary analyses used Cox proportional hazards regression of 2,682,440 male and 2,561,551 female offspring. Age was the time axis and robust standard errors were clustered by paternal identity. Adjustment set (e) (offspring sex and date of birth (DOB), maternal and paternal occupational and educational SEP, offspring birth order, and maternal age) was used. The secular trend per five years of offspring DOB was assessed using a similar model but without paternal age. The primary analysis was repeated with adjustment for paternal, not offspring, DOB to account for confounding, but not mediation, by secular trends. To examine whether the restricted dataset used for sibling-comparison analyses was representative of the main dataset, the primary analysis was repeated on this subset. Finally, the sibling-comparison analysis used Cox regression stratified by maternal identity and was restricted to offspring in families with discordant outcomes. All family-level confounding was intrinsically adjusted for and adjustment for offspring DOB or maternal age were not possible. Explicit adjustment was therefore limited to offspring sex and birth order.

				Hazard ratio per five years of father's age at offspring's birth (or per five years of offspring's DOB for secular trend)				
**Outcome**	**Deaths (main data)**	**Deaths (restricted data)**	**N (restricted data)**	**Primary analysis (main data)**	**Secular trend (main data)**	**Primary analysis plus mediation (main data)**	**Primary analysis (restricted data)**	**Sibling-comparison analysis (restricted data)**
All-cause	319,629	221,846	537,705	0.98 (0.97, 0.98)	1.07 (1.07, 1.08)	1.05 (1.04, 1.05)	0.99 (0.98, 0.99)	1.04 (1.03, 1.05)
Cardiovascular disease	78,671	49,030	130,360	0.96 (0.96, 0.97)	1.04 (1.04, 1.05)	1.01 (1.00, 1.02)	0.98 (0.97, 0.99)	1.01 (0.98, 1.04)
Coronary heart disease	42,601	26,517	72,930	0.95 (0.94, 0.97)	1.00 (0.99, 1.01)	0.96 (0.95, 0.98)	0.97 (0.97, 0.98)	0.96 (0.93, 1.00)
Aortic aneurysm	4,057	2,469	6,853	0.98 (0.94, 1.02)	1.05 (1.02, 1.08)	1.03 (0.99, 1.08)	0.96 (0.94, 0.99)	1.04 (0.92, 1.17)
Stroke	15,323	9,121	25,834	0.97 (0.95, 0.99)	1.01 (0.99, 1.02)	0.98 (0.96, 1.00)	0.98 (0.97, 0.99)	0.94 (0.88, 0.99)
Diabetes	6,503	3,976	11,226	0.99 (0.96, 1.02)	1.04 (1.02, 1.06)	1.03 (1.00, 1.07)	0.99 (0.97, 1.01)	0.99 (0.91, 1.08)
Respiratory diseases	12,887	7,637	21,250	0.96 (0.94, 0.98)	1.02 (1.00, 1.03)	0.98 (0.95, 1.00)	1.00 (0.99, 1.02)	1.02 (0.96, 1.09)
Kidney disease	1,529	898	2,501	0.95 (0.89, 1.02)	0.81 (0.77, 0.84)	0.77 (0.72, 0.83)	0.94 (0.91, 0.98)	0.69 (0.57, 0.84)
Pancreatic disease	928	607	1,816	0.94 (0.87, 1.02)	1.04 (0.99, 1.10)	0.98 (0.90, 1.08)	0.99 (0.93, 1.05)	1.18 (0.96, 1.44)
Liver cirrhosis	4,679	3,039	8,630	0.89 (0.86, 0.93)	1.05 (1.02, 1.08)	0.94 (0.90, 0.99)	0.99 (0.96, 1.01)	1.05 (0.95, 1.16)
Alcohol dependence	4,374	3,222	9,824	0.95 (0.91, 0.99)	1.01 (0.99, 1.04)	0.97 (0.93, 1.01)	0.95 (0.93, 0.97)	0.93 (0.85, 1.02)
Alzheimer's disease	2,876	1,263	3,230	1.00 (0.95, 1.04)	1.08 (1.01, 1.16)	1.08 (1.00, 1.16)	0.99 (0.96, 1.03)	1.16 (0.95, 1.42)
Parkinson's disease	713	291	739	0.95 (0.86, 1.05)	1.19 (1.03, 1.37)	1.14 (0.97, 1.34)	0.98 (0.92, 1.04)[a]	1.39 (0.83, 2.33)
Multiple sclerosis	1,395	923	2,587	1.04 (0.98, 1.11)	1.10 (1.05, 1.15)	1.14 (1.06, 1.23)	1.00 (0.97, 1.04)	1.08 (0.91, 1.29)
Motor neurone disease	1,964	1,227	3,468	1.01 (0.95, 1.06)	1.18 (1.12, 1.23)	1.18 (1.11, 1.27)	0.98 (0.95, 1.01)	0.97 (0.83, 1.14)
External causes	52,759	41,185	120,960	0.98 (0.97, 0.99)	1.08 (1.08, 1.09)	1.06 (1.05, 1.07)	0.97 (0.97, 0.98)	1.01 (0.99, 1.03)
Suicide	20,564	16,320	48,707	1.00 (0.98, 1.02)	1.10 (1.09, 1.11)	1.10 (1.08, 1.12)	0.98 (0.97, 0.99)	1.04 (1.01, 1.08)
Any cancer	118,305	78,094	205,865	0.99 (0.98, 0.99)	1.06 (1.05, 1.06)	1.04 (1.03, 1.05)	0.98 (0.98, 0.99)	1.04 (1.02, 1.07)
Malignant melanoma	3,733	2,495	6,950	0.95 (0.92, 0.99)	1.16 (1.13, 1.19)	1.11 (1.06, 1.16)	0.99 (0.96, 1.01)	1.18 (1.05, 1.31)
Brain cancer	6,392	4,659	13,286	1.03 (1.00, 1.06)	1.10 (1.08, 1.12)	1.13 (1.10, 1.17)	1.00 (0.98, 1.01)	1.12 (1.03, 1.20)
Oesophageal cancer	2,510	1,511	4,220	1.01 (0.96, 1.06)	1.15 (1.10, 1.20)	1.15 (1.08, 1.23)	0.98 (0.94, 1.01)	1.20 (1.03, 1.41)
Thyroid cancer	338	220	616	1.03 (0.91, 1.16)	1.10 (0.98, 1.23)	1.12 (0.96, 1.31)	0.99 (0.92, 1.06)	1.35 (0.92, 1.99)
Lymphatic cancer	10,517	6,924	19,625	0.99 (0.97, 1.02)	0.95 (0.94, 0.97)	0.95 (0.92, 0.97)	1.01 (0.99, 1.02)	1.05 (0.98, 1.12)

Lung cancer	21,053	13,455	37,634	0.97 (0.95, 0.99)	1.04 (1.02, 1.05)	1.01 (0.99, 1.03)	0.98 (0.97, 0.99)	1.00 (0.95, 1.05)
Breast cancer	11,359	5,190	12,511	1.01 (0.99, 1.04)	1.10 (1.08, 1.12)	1.11 (1.08, 1.14)	0.98 (0.97, 1.00)	1.10 (1.02, 1.19)
Liver cancer	3,133	1,920	5,418	0.92 (0.88, 0.97)	1.14 (1.10, 1.18)	1.06 (1.01, 1.12)	0.97 (0.95, 1.00)	1.01 (0.88, 1.15)
Gallbladder cancer	2,669	1,642	4,820	0.96 (0.92, 1.01)	1.10 (1.05, 1.15)	1.06 (1.00, 1.12)	0.98 (0.95, 1.01)	1.13 (0.98, 1.29)
Stomach cancer	3,730	2,518	7,531	1.02 (0.98, 1.06)	1.01 (0.98, 1.04)	1.03 (0.98, 1.08)	0.99 (0.97, 1.01)	0.99 (0.89, 1.11)
Kidney cancer	3,602	2,277	6,624	0.98 (0.94, 1.03)	0.95 (0.92, 0.99)	0.94 (0.89, 0.99)	0.97 (0.95, 0.99)	0.91 (0.81, 1.03)
Pancreatic cancer	8,386	5,173	14,624	0.97 (0.95, 1.00)	1.08 (1.05, 1.11)	1.05 (1.02, 1.09)	0.98 (0.96, 1.00)	0.98 (0.90, 1.06)
Colorectal cancer	12,637	7,772	21,667	0.99 (0.97, 1.02)	1.09 (1.07, 1.11)	1.09 (1.06, 1.12)	0.98 (0.96, 0.99)	1.07 (1.00, 1.14)
Bladder cancer	2,189	1,247	3,497	0.98 (0.93, 1.04)	1.06 (1.01, 1.11)	1.04 (0.97, 1.11)	0.96 (0.93, 0.99)	1.08 (0.90, 1.30)
Ovarian cancer	4,838	2,022	4,847	1.00 (0.97, 1.04)	1.07 (1.04, 1.10)	1.07 (1.03, 1.11)	0.95 (0.92, 0.97)	1.06 (0.93, 1.20)
Uterine cancer	2,857	1,146	2,829	0.99 (0.95, 1.04)	1.14 (1.11, 1.17)	1.13 (1.07, 1.19)	1.00 (0.96, 1.03)	1.03 (0.88, 1.21)
Prostate cancer	5,930	1,969	4,632	0.97 (0.94, 1.00)	0.91 (0.88, 0.95)	0.89 (0.85, 0.94)	0.98 (0.96, 1.01)	1.05 (0.89, 1.23)
Testicular cancer	280	136	325	1.01 (0.87, 1.16)	0.91 (0.85, 0.97)	0.92 (0.79, 1.07)	0.99 (0.87, 1.13)	0.97 (0.66, 1.43)

[a]This analysis failed for sons and daughters combined. The hazard ratio was estimated by meta-analysing separate results for sons and daughters.

Table S18. Primary analyses of offspring mortality and maternal age, with follow-up split by categories of offspring age. Cox proportional hazards regression was applied to 2,658,132 male and 2,546,301 female offspring. Age was the time axis and robust standard errors were clustered by maternal identity. Adjustment set (e) (offspring sex and date of birth, maternal and paternal occupational and educational SEP, offspring birth order, and paternal age) was used. Causes of death were omitted when there were fewer than five deaths in any age category. P-values from two tests for proportional hazards are reported. The first tested the correlation between log(age) and the scaled Schoenfeld residuals for parental age in the combined primary analysis of all ages of offspring. The second was a Wald test comparing the HR within each age class.

	Deaths in main dataset among offspring aged (years):				P_{PH} from test:		Hazard ratio per five years of mother's age at offspring's birth from primary analyses among offspring aged:			
**Outcome**	**18-40**	**40-49**	**50-59**	**≥60**	**1**	**2**	**18-40**	**40-49**	**50-59**	**≥60**
All-cause	25,354	33,929	77,521	156,666	0.012	0.043	0.97 (0.96, 0.98)	0.97 (0.96, 0.97)	0.96 (0.96, 0.97)	0.96 (0.95, 0.96)
Cardiovascular disease	2,236	6,734	19,982	47,400	0.966	<0.001	1.02 (0.98, 1.06)	0.96 (0.94, 0.98)	0.96 (0.94, 0.97)	0.94 (0.93, 0.95)
Coronary heart disease	583	3,311	11,733	26,226	0.072	<0.001	1.03 (0.96, 1.11)	0.98 (0.95, 1.01)	0.96 (0.94, 0.97)	0.94 (0.92, 0.95)
Aortic aneurysm	131	291	898	2,654	0.941	0.178	1.06 (0.91, 1.23)	0.90 (0.81, 0.99)	0.94 (0.88, 1.00)	0.91 (0.86, 0.95)
Stroke	425	1,399	3,540	9,328	0.034	0.094	0.92 (0.84, 1.01)	0.89 (0.85, 0.94)	0.94 (0.91, 0.97)	0.95 (0.93, 0.98)
Diabetes	382	721	1,454	3,399	0.328	0.413	1.00 (0.91, 1.10)	0.97 (0.91, 1.04)	0.93 (0.88, 0.98)	0.94 (0.90, 0.98)
Respiratory diseases	472	823	2,505	8,387	0.065	0.014	1.06 (0.97, 1.16)	0.99 (0.93, 1.05)	0.98 (0.94, 1.02)	0.94 (0.91, 0.97)
Kidney disease	53	110	289	819	0.192	0.858	1.05 (0.79, 1.39)	0.99 (0.84, 1.17)	0.97 (0.86, 1.09)	0.95 (0.87, 1.04)
Pancreatic disease	58	173	292	325	0.910	0.527	1.06 (0.85, 1.33)	1.01 (0.87, 1.17)	0.93 (0.82, 1.06)	0.92 (0.81, 1.04)
Liver cirrhosis	96	651	1,674	2,019	0.822	0.252	1.11 (0.90, 1.36)	1.04 (0.97, 1.13)	0.99 (0.93, 1.04)	0.97 (0.92, 1.03)
Alcohol dependence	209	935	1,712	1,245	0.831	0.756	0.95 (0.82, 1.09)	0.97 (0.91, 1.04)	0.94 (0.89, 1.00)	0.97 (0.91, 1.03)
Alzheimer's disease	0	25	191	2,657						
Parkinson's disease	0	2	35	676						
Multiple sclerosis	78	259	419	540	0.967	0.851	0.96 (0.76, 1.22)	0.99 (0.89, 1.11)	1.04 (0.94, 1.15)	1.01 (0.91, 1.11)
Motor neurone disease	44	147	531	1,192	0.866	0.799	0.97 (0.76, 1.25)	0.97 (0.84, 1.12)	0.98 (0.90, 1.07)	0.94 (0.88, 1.01)
External causes	13,693	8,531	9,237	7,197	0.033	0.504	0.90 (0.88, 0.92)	0.90 (0.88, 0.92)	0.90 (0.88, 0.92)	0.92 (0.89, 0.94)
Suicide	5,609	3,780	3,896	2,280	0.233	0.434	0.94 (0.91, 0.97)	0.93 (0.90, 0.96)	0.91 (0.88, 0.94)	0.93 (0.90, 0.97)
Any cancer	4,260	10,907	32,079	66,351	0.142	0.468	1.00 (0.97, 1.03)	0.98 (0.96, 0.99)	0.98 (0.97, 0.99)	0.98 (0.97, 0.99)
Malignant melanoma	313	577	1,051	1,531	0.965	0.255	1.14 (1.03, 1.26)	1.02 (0.94, 1.10)	1.06 (1.00, 1.13)	1.04 (0.98, 1.10)
Brain cancer	731	991	1,785	2,317	0.465	0.639	0.95 (0.88, 1.03)	0.96 (0.90, 1.02)	0.99 (0.94, 1.04)	0.96 (0.92, 1.01)
Oesophageal cancer	16	175	771	1,520	0.155	0.543	1.09 (0.74, 1.62)	1.00 (0.88, 1.14)	1.00 (0.93, 1.08)	0.95 (0.89, 1.02)
Thyroid cancer	11	25	75	212	0.657	0.740	0.82 (0.39, 1.71)	1.00 (0.70, 1.41)	0.80 (0.63, 1.01)	0.82 (0.70, 0.98)
Lymphatic cancer	679	887	2,397	5,445	0.131	0.947	1.00 (0.94, 1.08)	0.99 (0.94, 1.05)	1.01 (0.97, 1.05)	1.01 (0.98, 1.04)
Lung cancer	178	1,323	5,875	13,376	0.379	0.084	0.91 (0.79, 1.05)	0.92 (0.88, 0.97)	0.93 (0.90, 0.95)	0.96 (0.94, 0.98)
Breast cancer	544	2,026	3,958	4,359	0.880	0.536	0.99 (0.91, 1.06)	1.01 (0.97, 1.05)	1.03 (1.00, 1.07)	1.02 (0.98, 1.05)
Liver cancer	74	249	911	1,819	0.113	0.129	1.20 (0.98, 1.47)	0.98 (0.87, 1.10)	0.94 (0.88, 1.01)	0.97 (0.92, 1.03)
Gallbladder cancer	44	214	752	1,616	0.519	0.894	1.08 (0.86, 1.37)	0.98 (0.87, 1.10)	1.01 (0.94, 1.08)	1.01 (0.95, 1.07)

Stomach cancer	115	422	1,036	2,007	0.141	0.399	0.87 (0.73, 1.05)	0.91 (0.83, 0.99)	0.87 (0.82, 0.93)	0.92 (0.87, 0.97)
Kidney cancer	45	253	1,060	2,157	0.411	0.750	0.86 (0.68, 1.09)	0.91 (0.82, 1.01)	0.91 (0.85, 0.97)	0.94 (0.89, 0.99)
Pancreatic cancer	87	562	2,286	5,323	0.898	0.128	0.87 (0.71, 1.06)	0.94 (0.87, 1.01)	0.96 (0.92, 1.00)	0.99 (0.96, 1.03)
Colorectal cancer	342	1,015	3,244	7,724	0.992	0.075	1.05 (0.96, 1.15)	1.00 (0.95, 1.05)	0.96 (0.92, 0.99)	0.95 (0.92, 0.98)
Bladder cancer	25	107	478	1,563	0.269	0.309	0.83 (0.62, 1.11)	0.96 (0.81, 1.15)	1.06 (0.97, 1.15)	1.00 (0.94, 1.07)
Ovarian cancer	138	548	1,641	2,355	0.929	0.303	1.13 (0.97, 1.32)	0.99 (0.92, 1.06)	1.00 (0.95, 1.05)	0.98 (0.93, 1.02)
Uterine cancer	215	366	743	1,348	0.782	0.054	0.95 (0.84, 1.06)	0.93 (0.85, 1.02)	1.03 (0.95, 1.11)	0.93 (0.87, 0.99)
Prostate cancer	3	57	831	5,028						
Testicular cancer	67	25	16	25	0.473	0.966	1.12 (0.81, 1.56)	1.19 (0.84, 1.70)	1.09 (0.66, 1.79)	1.06 (0.71, 1.59)

Table S19. Primary analyses of offspring mortality and paternal age, with follow-up split by categories of offspring age. Cox proportional hazards regression was applied to 2,658,132 male and 2,546,301 female offspring. Age was the time axis and robust standard errors were clustered by paternal identity. Adjustment set (e) (offspring sex and date of birth, maternal and paternal occupational and educational SEP, offspring birth order, and maternal age) was used. Causes of death were omitted when there were fewer than five deaths in any age category. P-values from two tests for proportional hazards are reported. The first tested the correlation between log(age) and the scaled Schoenfeld residuals for parental age in the combined primary analysis of all ages of offspring. The second was a Wald test comparing the HR within each age class.

	Deaths in main dataset among offspring aged (years):				P_{PH} from test:		Hazard ratio per five years of father's age at offspring's birth from primary analyses among offspring aged:			
Outcome	18-40	40-49	50-59	≥60	1	2	18-40	40-49	50-59	≥60
All-cause	25,354	33,929	77,521	156,666	0.072	<0.001	1.01 (1.00, 1.02)	0.99 (0.98, 0.99)	0.97 (0.97, 0.98)	0.97 (0.96, 0.97)
Cardiovascular disease	2,236	6,734	19,982	47,400	0.770	<0.001	1.05 (1.02, 1.08)	0.99 (0.97, 1.01)	0.97 (0.96, 0.98)	0.96 (0.95, 0.97)
Coronary heart disease	583	3,311	11,733	26,226	0.107	<0.001	1.05 (0.99, 1.12)	1.00 (0.97, 1.02)	0.96 (0.95, 0.98)	0.95 (0.94, 0.96)
Aortic aneurysm	131	291	898	2,654	0.824	0.082	1.14 (1.01, 1.29)	0.96 (0.87, 1.05)	1.00 (0.94, 1.05)	0.98 (0.94, 1.03)
Stroke	425	1,399	3,540	9,328	0.385	0.430	0.97 (0.90, 1.05)	0.95 (0.91, 0.99)	0.95 (0.93, 0.98)	0.97 (0.95, 1.00)
Diabetes	382	721	1,454	3,399	0.792	0.179	1.04 (0.96, 1.12)	1.02 (0.96, 1.08)	0.96 (0.92, 1.01)	0.99 (0.95, 1.02)
Respiratory diseases	472	823	2,505	8,387	0.109	<0.001	1.07 (0.99, 1.15)	0.98 (0.94, 1.04)	0.97 (0.94, 1.01)	0.94 (0.91, 0.96)
Kidney disease	53	110	289	819	0.591	0.902	0.88 (0.70, 1.10)	0.95 (0.84, 1.09)	0.96 (0.87, 1.06)	0.96 (0.88, 1.04)
Pancreatic disease	58	173	292	325	0.718	0.816	1.01 (0.81, 1.25)	0.95 (0.83, 1.08)	0.93 (0.83, 1.04)	0.91 (0.82, 1.01)
Liver cirrhosis	96	651	1,674	2,019	0.987	0.254	0.97 (0.84, 1.14)	0.92 (0.86, 0.98)	0.87 (0.83, 0.92)	0.87 (0.83, 0.91)
Alcohol dependence	209	935	1,712	1,245	0.938	0.891	0.96 (0.85, 1.07)	0.96 (0.91, 1.02)	0.94 (0.90, 0.99)	0.96 (0.91, 1.01)
Alzheimer's disease	0	25	191	2,657						
Parkinson's disease	0	2	35	676						
Multiple sclerosis	78	259	419	540	0.586	0.953	1.07 (0.91, 1.25)	1.05 (0.94, 1.16)	1.05 (0.97, 1.14)	1.03 (0.95, 1.12)
Motor neurone disease	44	147	531	1,192	0.050	0.498	0.91 (0.67, 1.24)	1.02 (0.91, 1.15)	1.05 (0.97, 1.13)	1.00 (0.94, 1.06)
External causes	13,693	8,531	9,237	7,197	0.008	0.002	1.01 (0.99, 1.03)	0.98 (0.96, 0.99)	0.97 (0.95, 0.99)	0.98 (0.96, 1.00)
Suicide	5,609	3,780	3,896	2,280	0.050	<0.001	1.04 (1.01, 1.07)	1.01 (0.98, 1.04)	0.97 (0.94, 1.00)	0.99 (0.96, 1.03)
Any cancer	4,260	10,907	32,079	66,351	0.319	0.112	1.01 (0.99, 1.03)	0.99 (0.98, 1.01)	0.99 (0.98, 1.00)	0.98 (0.97, 0.99)
Malignant melanoma	313	577	1,051	1,531	0.227	0.636	0.98 (0.89, 1.07)	0.93 (0.87, 1.00)	0.97 (0.92, 1.02)	0.94 (0.89, 0.99)
Brain cancer	731	991	1,785	2,317	0.963	0.772	1.03 (0.97, 1.10)	1.05 (0.99, 1.10)	1.04 (0.99, 1.08)	1.02 (0.98, 1.06)
Oesophageal cancer	16	175	771	1,520	0.311	0.305	1.21 (0.77, 1.89)	1.09 (0.97, 1.22)	1.02 (0.96, 1.09)	0.99 (0.93, 1.05)
Thyroid cancer	11	25	75	212	0.544	0.962	1.08 (0.72, 1.64)	1.03 (0.77, 1.39)	0.97 (0.79, 1.19)	1.01 (0.87, 1.16)
Lymphatic cancer	679	887	2,397	5,445	0.326	0.309	1.02 (0.96, 1.08)	1.00 (0.95, 1.05)	1.01 (0.97, 1.04)	0.98 (0.95, 1.01)
Lung cancer	178	1,323	5,875	13,376	0.604	0.470	0.94 (0.84, 1.05)	0.98 (0.94, 1.02)	0.96 (0.94, 0.99)	0.98 (0.96, 1.00)
Breast cancer	544	2,026	3,958	4,359	0.611	0.943	1.04 (0.97, 1.11)	1.01 (0.98, 1.05)	1.02 (0.99, 1.05)	1.01 (0.98, 1.04)
Liver cancer	74	249	911	1,819	0.212	0.349	1.04 (0.88, 1.23)	0.92 (0.83, 1.01)	0.90 (0.84, 0.96)	0.92 (0.88, 0.97)
Gallbladder cancer	44	214	752	1,616	0.315	0.345	0.97 (0.79, 1.19)	0.90 (0.81, 1.00)	0.99 (0.93, 1.06)	0.96 (0.91, 1.01)

Stomach cancer	115	422	1,036	2,007	0.339	0.677	1.03 (0.89, 1.20)	1.06 (0.99, 1.14)	1.01 (0.95, 1.07)	1.03 (0.98, 1.08)
Kidney cancer	45	253	1,060	2,157	0.466	0.429	0.88 (0.70, 1.09)	0.94 (0.85, 1.03)	1.00 (0.94, 1.06)	0.99 (0.95, 1.04)
Pancreatic cancer	87	562	2,286	5,323	0.708	0.192	0.86 (0.72, 1.03)	0.95 (0.90, 1.02)	0.96 (0.92, 1.00)	0.99 (0.96, 1.02)
Colorectal cancer	342	1,015	3,244	7,724	0.541	0.248	1.04 (0.96, 1.12)	1.03 (0.98, 1.08)	0.98 (0.95, 1.02)	1.00 (0.97, 1.02)
Bladder cancer	25	107	478	1,563	0.544	0.269	0.81 (0.55, 1.18)	0.93 (0.80, 1.08)	1.03 (0.95, 1.12)	0.98 (0.92, 1.04)
Ovarian cancer	138	548	1,641	2,355	0.581	0.966	1.02 (0.89, 1.17)	1.00 (0.94, 1.06)	1.00 (0.96, 1.04)	1.01 (0.97, 1.05)
Uterine cancer	215	366	743	1,348	0.832	0.502	1.03 (0.92, 1.14)	0.97 (0.88, 1.06)	1.03 (0.96, 1.09)	0.99 (0.94, 1.04)
Prostate cancer	3	57	831	5,028						
Testicular cancer	67	25	16	25	0.834	0.852	1.00 (0.76, 1.30)	0.97 (0.71, 1.32)	0.82 (0.55, 1.23)	0.89 (0.61, 1.30)

Table S20. Primary and sibling-comparison analyses of offspring mortality and maternal age, made separately for men (sons) and women (daughters). Primary analyses used Cox proportional hazards regression of 2,658,132 male and 2,546,301 female offspring. Age was the time axis and robust standard errors were clustered by maternal identity. Adjustment set (e) (offspring date of birth (DOB), maternal and paternal occupational and educational SEP, offspring birth order, and paternal age) was used. The sibling-comparison analysis used Cox regression stratified by maternal identity and was restricted to same-sex offspring in families with discordant outcomes. All family-level confounding was intrinsically adjusted for and adjustment for offspring DOB or paternal age were not possible. Explicit adjustment was therefore limited to offspring birth order.

	**Number of deaths analysed**				**Hazard ratio per five years of mother's age at offspring's birth**			
	**Primary analysis (main data)**		**Sibling-comparison analysis (restricted data)**		**Primary analysis (main data)**		**Sibling-comparison analysis (restricted data)**	
**Outcome**	**sons**	**daughters**	**sons**	**daughters**	**sons**	**daughters**	**sons**	**daughters**
All-cause	180,057	113,413	76,168	46,563	0.96 (0.95, 0.97)	0.97 (0.96, 0.97)	0.93 (0.91, 0.96)	0.97 (0.94, 1.00)
Cardiovascular disease	54,607	21,745	21,231	7,879	0.95 (0.94, 0.97)	0.94 (0.92, 0.95)	0.85 (0.81, 0.89)	1.01 (0.94, 1.10)
Coronary heart disease	32,282	9,571	12,608	3,484	0.96 (0.94, 0.97)	0.92 (0.89, 0.94)	0.79 (0.74, 0.84)	0.92 (0.81, 1.03)
Aortic aneurysm	2,881	1,093	1,115	368	0.92 (0.88, 0.97)	0.90 (0.83, 0.99)	1.01 (0.82, 1.25)	0.81 (0.54, 1.19)
Stroke	8,876	5,816	3,163	2,118	0.94 (0.91, 0.97)	0.94 (0.91, 0.98)	0.78 (0.69, 0.89)	1.00 (0.85, 1.16)
Diabetes	4,027	1,929	1,461	692	0.95 (0.91, 0.99)	0.94 (0.88, 1.00)	1.06 (0.88, 1.26)	0.97 (0.75, 1.25)
Respiratory diseases	6,578	5,609	2,327	2,127	0.97 (0.94, 1.01)	0.95 (0.91, 0.99)	0.85 (0.74, 0.98)	0.89 (0.76, 1.04)
Kidney disease	743	528	246	195	0.98 (0.88, 1.09)	0.94 (0.83, 1.06)	0.90 (0.57, 1.43)	0.81 (0.49, 1.35)
Pancreatic disease	631	217	257	79	0.96 (0.86, 1.07)	0.93 (0.75, 1.14)	0.90 (0.63, 1.28)	1.20 (0.52, 2.78)
Liver cirrhosis	3,194	1,246	1,207	467	1.00 (0.95, 1.06)	0.97 (0.89, 1.06)	1.09 (0.91, 1.32)	0.98 (0.73, 1.31)
Alcohol dependence	3,413	688	1,571	302	0.96 (0.91, 1.01)	0.96 (0.85, 1.07)	0.81 (0.69, 0.94)	0.84 (0.59, 1.21)
Alzheimer's disease	1,353	1,520	377	391	1.03 (0.95, 1.11)	1.05 (0.97, 1.13)	1.06 (0.72, 1.56)	1.75 (1.16, 2.64)
Parkinson's disease	462	251	113	63	1.12 (0.98, 1.27)	1.09 (0.90, 1.33)	1.39 (0.63, 3.07)	0.90 (0.32, 2.54)
Multiple sclerosis	536	760	206	290	1.04 (0.92, 1.17)	0.99 (0.90, 1.10)	1.42 (0.83, 2.41)	1.08 (0.71, 1.67)
Motor neurone disease	1,121	793	450	301	0.99 (0.91, 1.08)	0.91 (0.83, 1.01)	1.00 (0.72, 1.40)	1.00 (0.68, 1.46)
External causes	28,699	9,959	12,972	4,306	0.90 (0.88, 0.91)	0.92 (0.89, 0.94)	0.95 (0.89, 1.00)	0.81 (0.73, 0.89)
Suicide	11,307	4,258	5,093	1,901	0.93 (0.91, 0.96)	0.92 (0.88, 0.96)	0.92 (0.85, 1.01)	0.88 (0.76, 1.02)
Any cancer	56,871	56,726	22,538	23,323	0.98 (0.96, 0.99)	0.98 (0.97, 0.99)	0.96 (0.92, 1.01)	0.96 (0.92, 1.00)
Malignant melanoma	2,125	1,347	797	532	1.08 (1.01, 1.14)	1.02 (0.94, 1.09)	1.10 (0.87, 1.40)	1.29 (0.96, 1.74)
Brain cancer	3,525	2,299	1,576	970	0.97 (0.92, 1.01)	0.97 (0.92, 1.03)	0.94 (0.79, 1.12)	0.91 (0.73, 1.13)
Oesophageal cancer	1,968	514	724	184	0.95 (0.89, 1.01)	1.08 (0.94, 1.25)	0.98 (0.77, 1.26)	2.86 (1.54, 5.32)
Thyroid cancer	170	153	63	60	0.78 (0.63, 0.97)	0.89 (0.73, 1.09)	2.17 (0.95, 4.96)	3.69 (1.24, 10.99)
Lymphatic cancer	5,664	3,744	2,226	1,427	1.02 (0.98, 1.06)	0.99 (0.94, 1.03)	0.99 (0.86, 1.16)	0.95 (0.79, 1.14)
Lung cancer	10,489	10,263	4,111	4,301	0.95 (0.92, 0.98)	0.94 (0.91, 0.97)	0.93 (0.83, 1.03)	0.96 (0.87, 1.07)
Liver cancer	2,048	1,005	771	384	0.97 (0.91, 1.04)	0.96 (0.88, 1.05)	0.97 (0.77, 1.23)	0.74 (0.52, 1.07)

Gallbladder cancer	934	1,692	373	624	1.03 (0.95, 1.12)	0.99 (0.93, 1.06)	0.84 (0.59, 1.20)	1.08 (0.83, 1.42)
Stomach cancer	2,249	1,331	947	586	0.90 (0.85, 0.96)	0.90 (0.83, 0.97)	0.90 (0.73, 1.13)	0.63 (0.47, 0.85)
Kidney cancer	2,233	1,282	859	504	0.92 (0.86, 0.97)	0.94 (0.87, 1.02)	0.78 (0.62, 0.99)	0.79 (0.58, 1.07)
Pancreatic cancer	4,369	3,889	1,663	1,489	0.98 (0.93, 1.02)	0.98 (0.93, 1.02)	1.00 (0.85, 1.18)	0.92 (0.77, 1.10)
Colorectal cancer	6,827	5,498	2,589	2,113	0.97 (0.93, 1.00)	0.95 (0.92, 0.99)	1.05 (0.92, 1.20)	1.01 (0.87, 1.18)
Bladder cancer	1,591	582	569	210	0.99 (0.92, 1.07)	1.06 (0.94, 1.19)	0.96 (0.71, 1.30)	0.87 (0.52, 1.45)

Table S21. Primary and sibling-comparison analyses of offspring mortality and paternal age, made separately for men (sons) and women (daughters).
Primary analyses used Cox proportional hazards regression of 2,658,132 male and 2,546,301 female offspring. Age was the time axis and robust standard errors were clustered by paternal identity. Adjustment set (e) (offspring date of birth (DOB), maternal and paternal occupational and educational SEP, offspring birth order, and maternal age) was used. The sibling-comparison analysis used Cox regression stratified by paternal identity and was restricted to same-sex offspring in families with discordant outcomes. All family-level confounding was intrinsically adjusted for and adjustment for offspring DOB or maternal age were not possible. Explicit adjustment was therefore limited to offspring birth order.

	**Number of deaths analysed**				**Hazard ratio per five years of father's age at offspring's birth**			
	**Primary analysis (main data)**		**Sibling-comparison analysis (restricted data)**		**Primary analysis (main data)**		**Sibling-comparison analysis (restricted data)**	
**Outcome**	**sons**	**daughters**	**sons**	**daughters**	**sons**	**daughters**	**sons**	**daughters**
All-cause	180,057	113,413	76,387	46,709	0.97 (0.96, 0.98)	0.99 (0.98, 0.99)	0.91 (0.89, 0.93)	0.97 (0.94, 1.00)
Cardiovascular disease	54,607	21,745	21,270	7,899	0.96 (0.95, 0.97)	0.98 (0.96, 1.00)	0.85 (0.81, 0.88)	0.97 (0.90, 1.03)
Coronary heart disease	32,282	9,571	12,644	3,507	0.95 (0.94, 0.97)	0.98 (0.96, 1.01)	0.79 (0.75, 0.84)	0.87 (0.78, 0.96)
Aortic aneurysm	2,881	1,093	1,109	371	1.00 (0.95, 1.05)	0.96 (0.89, 1.03)	0.92 (0.77, 1.11)	0.89 (0.63, 1.27)
Stroke	8,876	5,816	3,174	2,089	0.97 (0.94, 0.99)	0.97 (0.94, 1.00)	0.79 (0.71, 0.88)	0.92 (0.81, 1.06)
Diabetes	4,027	1,929	1,468	690	0.98 (0.95, 1.03)	1.00 (0.94, 1.06)	0.96 (0.82, 1.12)	0.93 (0.74, 1.19)
Respiratory diseases	6,578	5,609	2,324	2,126	0.96 (0.93, 0.99)	0.94 (0.91, 0.98)	0.86 (0.76, 0.98)	0.83 (0.72, 0.95)
Kidney disease	743	528	245	194	0.97 (0.88, 1.06)	0.94 (0.85, 1.04)	0.74 (0.48, 1.14)	0.75 (0.45, 1.25)
Pancreatic disease	631	217	255	76	0.90 (0.82, 0.99)	1.06 (0.88, 1.27)	0.85 (0.63, 1.16)	1.11 (0.51, 2.39)
Liver cirrhosis	3,194	1,246	1,218	475	0.89 (0.85, 0.93)	0.86 (0.80, 0.93)	1.06 (0.90, 1.24)	0.91 (0.72, 1.15)
Alcohol dependence	3,413	688	1,576	293	0.95 (0.91, 0.99)	0.95 (0.86, 1.05)	0.73 (0.63, 0.84)	0.69 (0.49, 0.97)
Alzheimer's disease	1,353	1,520	376	401	0.99 (0.92, 1.06)	1.01 (0.95, 1.07)	1.05 (0.72, 1.52)	1.54 (1.06, 2.24)
Parkinson's disease	462	251	117	62	0.98 (0.87, 1.11)	0.89 (0.75, 1.06)	1.24 (0.57, 2.69)	1.03 (0.36, 2.92)
Multiple sclerosis	536	760	211	296	1.02 (0.93, 1.12)	1.06 (0.97, 1.16)	0.91 (0.62, 1.36)	1.12 (0.78, 1.61)
Motor neurone disease	1,121	793	452	303	1.03 (0.96, 1.10)	0.99 (0.91, 1.08)	0.87 (0.64, 1.19)	1.06 (0.74, 1.52)
External causes	28,699	9,959	13,042	4,320	0.99 (0.97, 1.00)	0.98 (0.96, 1.01)	0.93 (0.89, 0.97)	0.90 (0.84, 0.98)
Suicide	11,307	4,258	5,144	1,919	1.01 (0.98, 1.03)	1.02 (0.98, 1.06)	0.95 (0.89, 1.02)	1.00 (0.89, 1.11)
Any cancer	56,871	56,726	22,637	23,406	0.98 (0.97, 0.99)	0.99 (0.98, 1.00)	0.94 (0.90, 0.98)	0.97 (0.93, 1.01)
Malignant melanoma	2,125	1,347	805	546	0.94 (0.89, 0.99)	0.97 (0.90, 1.03)	1.26 (1.01, 1.56)	1.17 (0.90, 1.51)
Brain cancer	3,525	2,299	1,575	975	1.04 (1.00, 1.08)	1.02 (0.97, 1.07)	0.96 (0.83, 1.10)	0.90 (0.73, 1.11)
Oesophageal cancer	1,968	514	735	190	1.02 (0.97, 1.08)	0.96 (0.85, 1.08)	1.02 (0.82, 1.26)	2.87 (1.60, 5.13)
Thyroid cancer	170	153	64	61	1.08 (0.92, 1.26)	0.93 (0.77, 1.13)	1.32 (0.70, 2.50)	3.83 (1.30, 11.30)
Lymphatic cancer	5,664	3,744	2,250	1,420	0.98 (0.95, 1.01)	1.01 (0.97, 1.05)	0.93 (0.81, 1.05)	0.97 (0.82, 1.15)
Lung cancer	10,489	10,263	4,109	4,279	0.98 (0.95, 1.00)	0.97 (0.95, 1.00)	0.91 (0.82, 1.00)	0.96 (0.88, 1.06)
Liver cancer	2,048	1,005	775	384	0.88 (0.83, 0.94)	0.99 (0.91, 1.07)	0.98 (0.80, 1.21)	0.86 (0.64, 1.17)

Gallbladder cancer	934	1,692	374	625	0.97 (0.90, 1.04)	0.96 (0.91, 1.02)	0.87 (0.65, 1.16)	1.09 (0.85, 1.39)
Stomach cancer	2,249	1,331	954	587	1.03 (0.97, 1.08)	1.03 (0.96, 1.11)	0.86 (0.71, 1.05)	0.80 (0.63, 1.03)
Kidney cancer	2,233	1,282	868	514	1.00 (0.95, 1.05)	0.97 (0.90, 1.04)	0.74 (0.60, 0.92)	0.88 (0.69, 1.11)
Pancreatic cancer	4,369	3,889	1,660	1,502	0.96 (0.93, 1.00)	0.99 (0.95, 1.03)	0.90 (0.78, 1.04)	0.93 (0.79, 1.10)
Colorectal cancer	6,827	5,498	2,617	2,122	1.00 (0.97, 1.03)	0.99 (0.96, 1.03)	0.98 (0.87, 1.10)	1.03 (0.90, 1.18)
Bladder cancer	1,591	582	567	217	1.01 (0.95, 1.08)	0.93 (0.83, 1.03)	1.00 (0.76, 1.33)	0.77 (0.48, 1.24)

Figure S1a. Recorded all-cause and cause-specific deaths by year of death among the study population (born 1932-1987). Causes of death were recorded using four successive versions of the international classification of diseases (ICD) system. The solid black line indicates the numbers of deaths expected if the proportion of deaths due to each cause had remained constant over time.

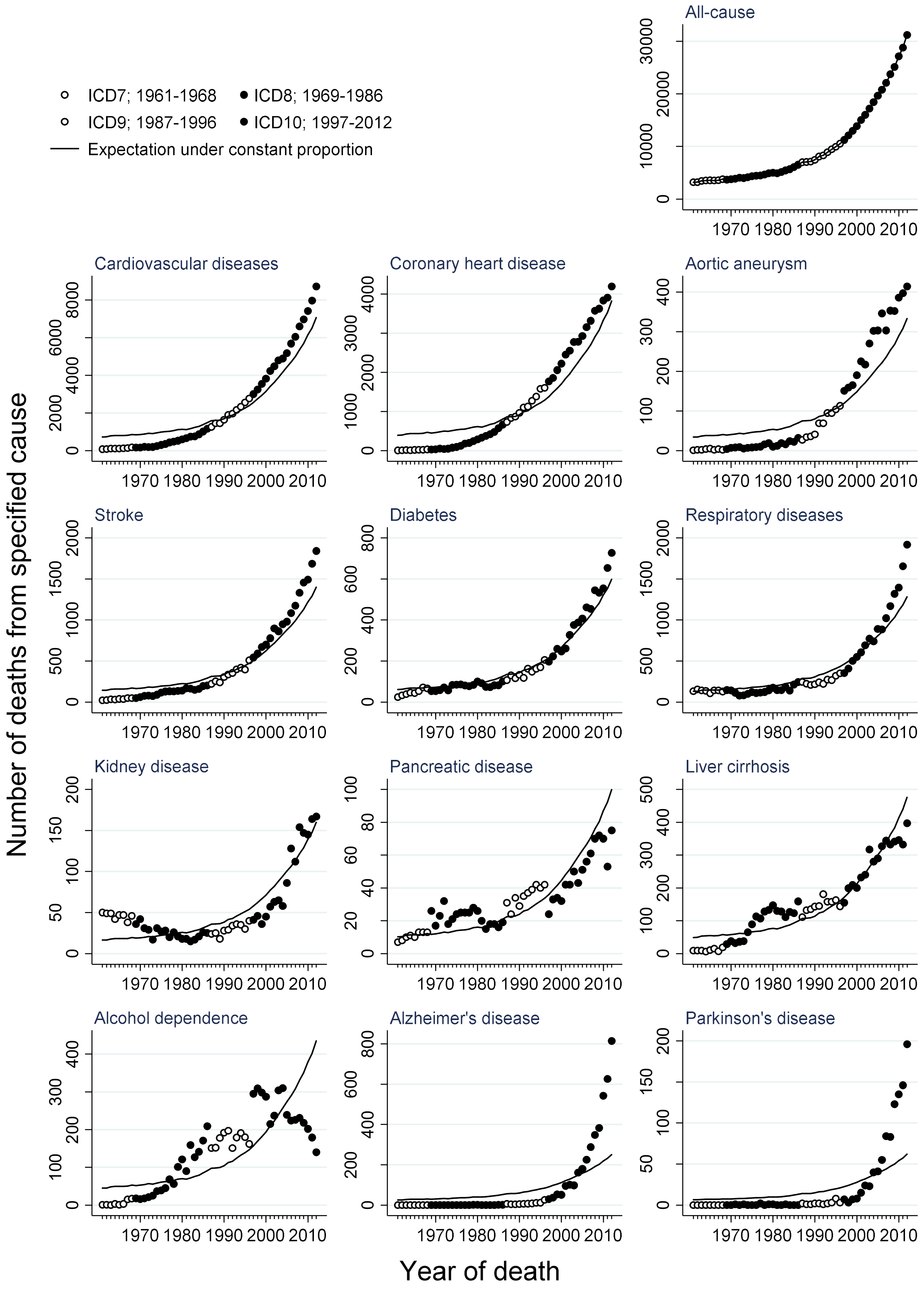

Figure S1b. Recorded all-cause and cause-specific deaths by year of death among the study population (born 1932-1987). Causes of death were recorded using four successive versions of the international classification of diseases (ICD) system. The solid black line indicates the numbers of deaths expected if the proportion of deaths due to each cause had remained constant over time.

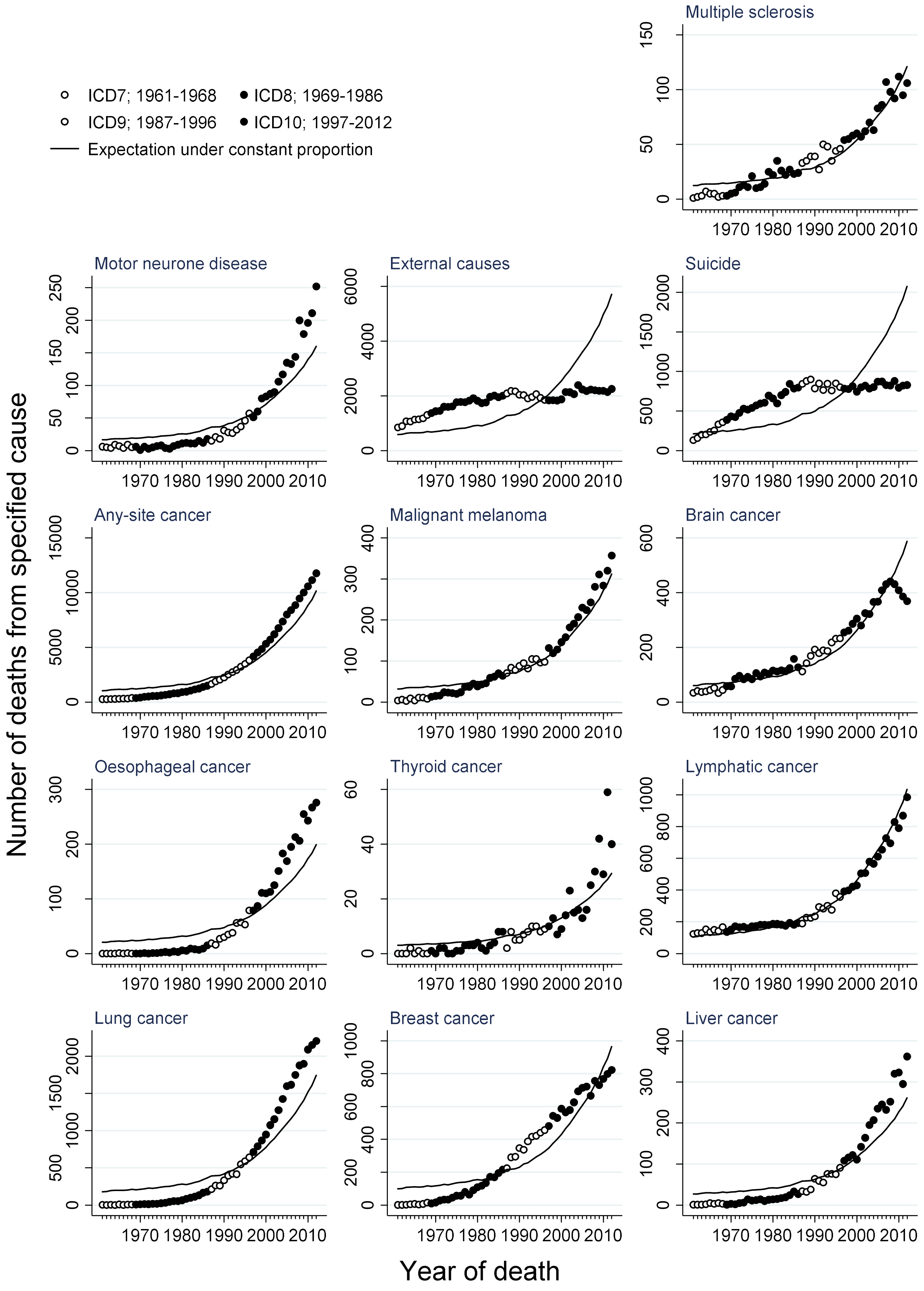

Figure S1c. Recorded all-cause and cause-specific deaths by year of death among the study population (born 1932-1987). Causes of death were recorded using four successive versions of the international classification of diseases (ICD) system. The solid black line indicates the numbers of deaths expected if the proportion of deaths due to each cause had remained constant over time.

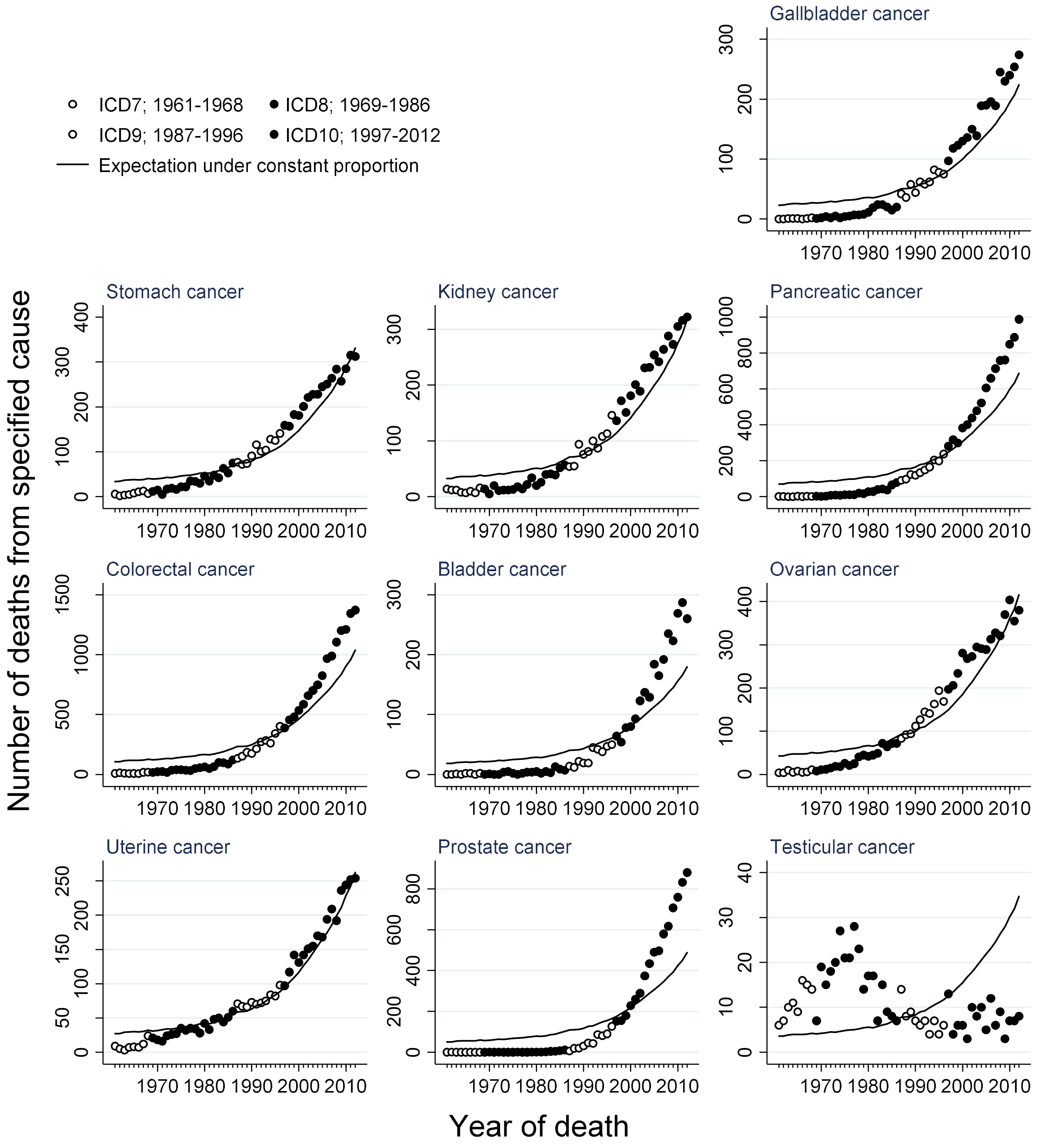

Figure S2. Hazard ratios for offspring mortality against classes of maternal or paternal age. Parental age in years was put into classes of <20, 20-24 (reference), 25-29, 30-34, 35-39, 40-44 and ≥45 and each class was plotted at its median. Points, but not lines, are transposed horizontally by +/- 0.5 years for clarity. The two oldest classes were combined for maternal age. Error bars are 95% confidence intervals. Primary analyses used Cox proportional hazards regression with robust standard errors clustered by parental identity. Standard adjustment comprised offspring sex, DOB and birth order, maternal and paternal occupational and educational SEP and the other parent's age at the time of the offspring's birth. Sibling-comparison analyses used Cox regression stratified by parental identity. They were conducted on the restricted dataset and adjusted for offspring sex and birth order (adjustment for parental SEP was unnecessary and adjustment for offspring DOB and the other parent's age were impossible).

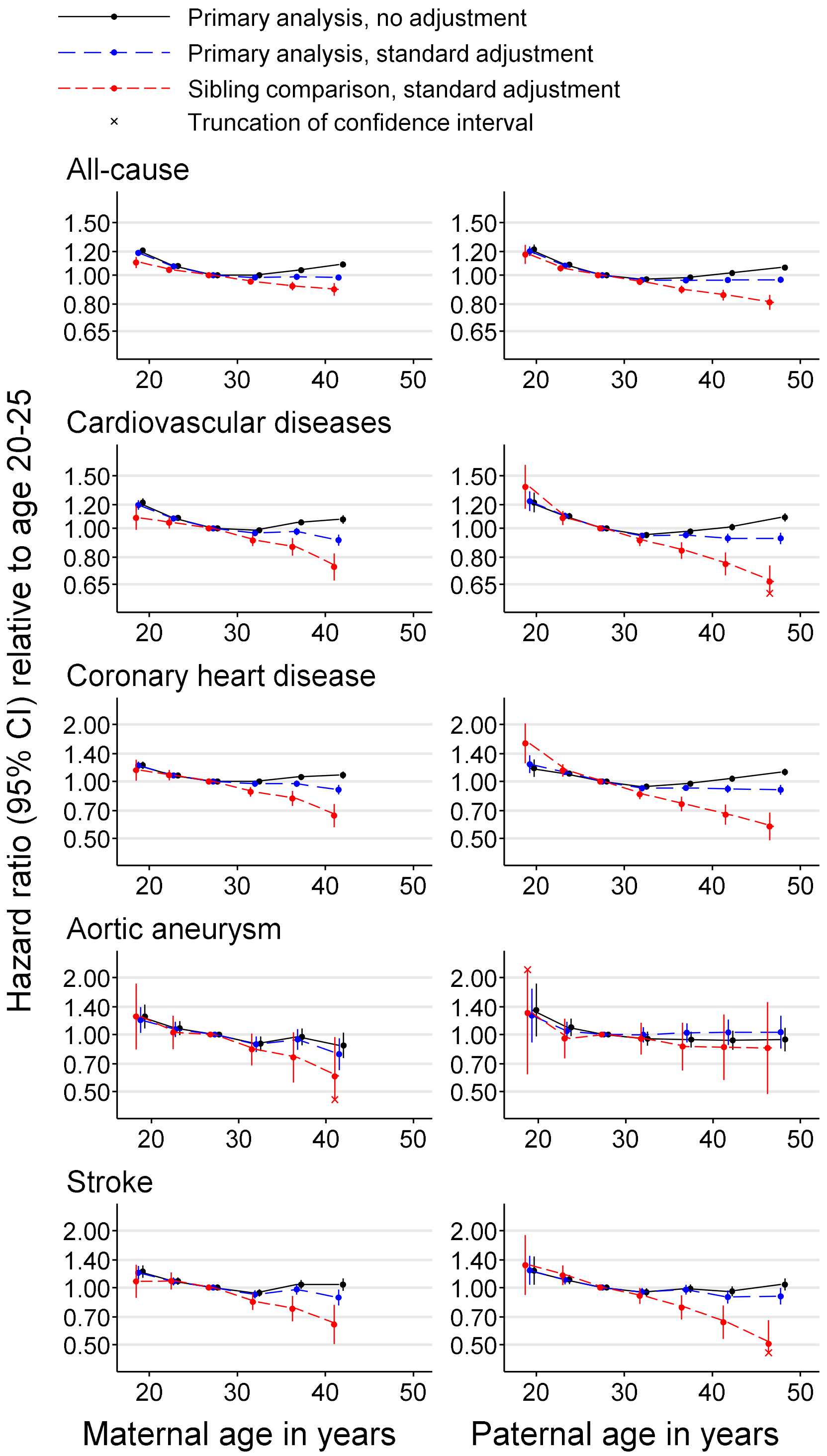

Figure S2 (continued). Hazard ratios for offspring mortality against classes of maternal or paternal age.

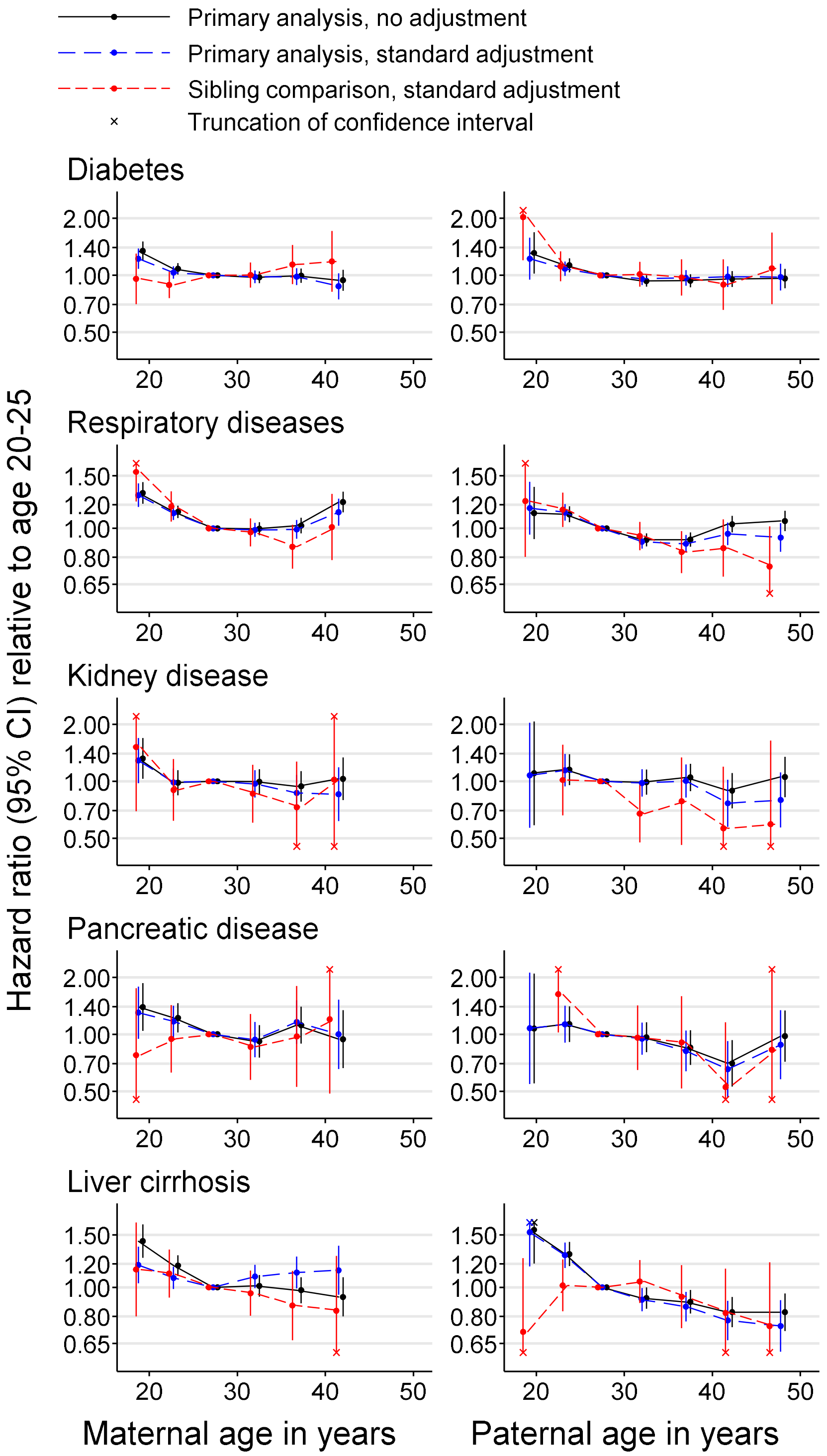

Figure S2 (continued). Hazard ratios for offspring mortality against classes of maternal or paternal age.

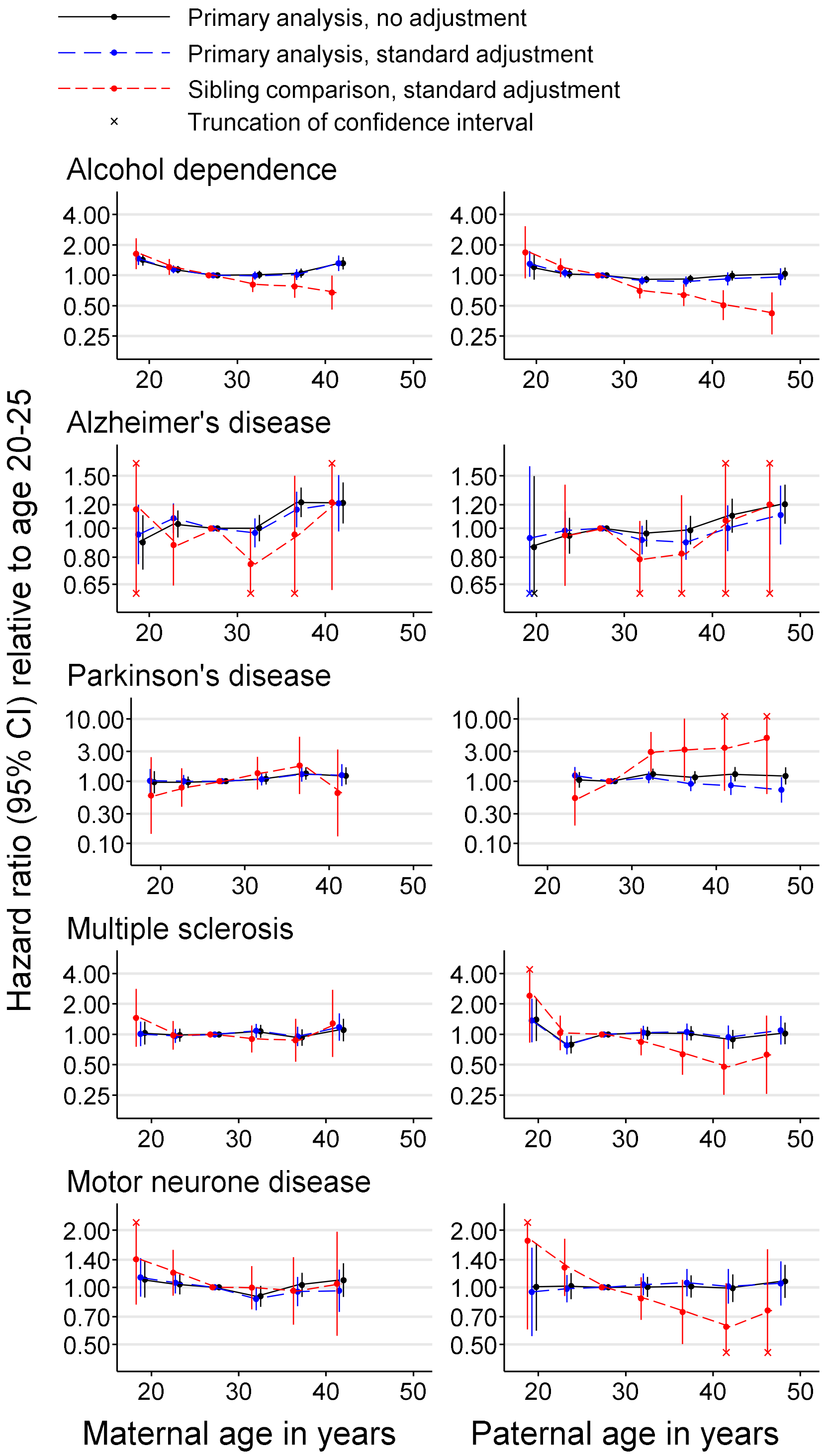

Figure S2 (continued). Hazard ratios for offspring mortality against classes of maternal or paternal age.

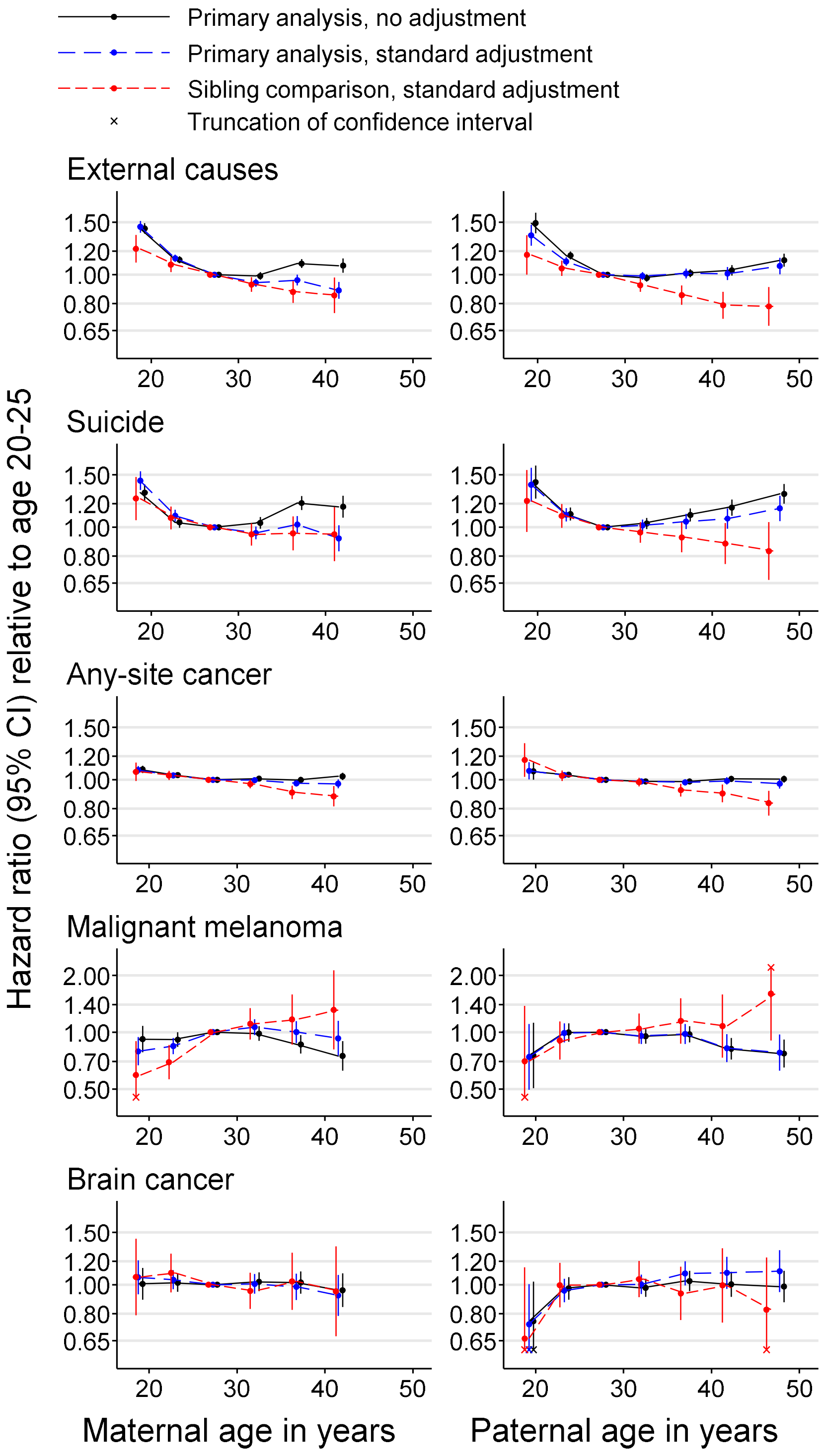

Figure S2 (continued). Hazard ratios for offspring mortality against classes of maternal or paternal age.

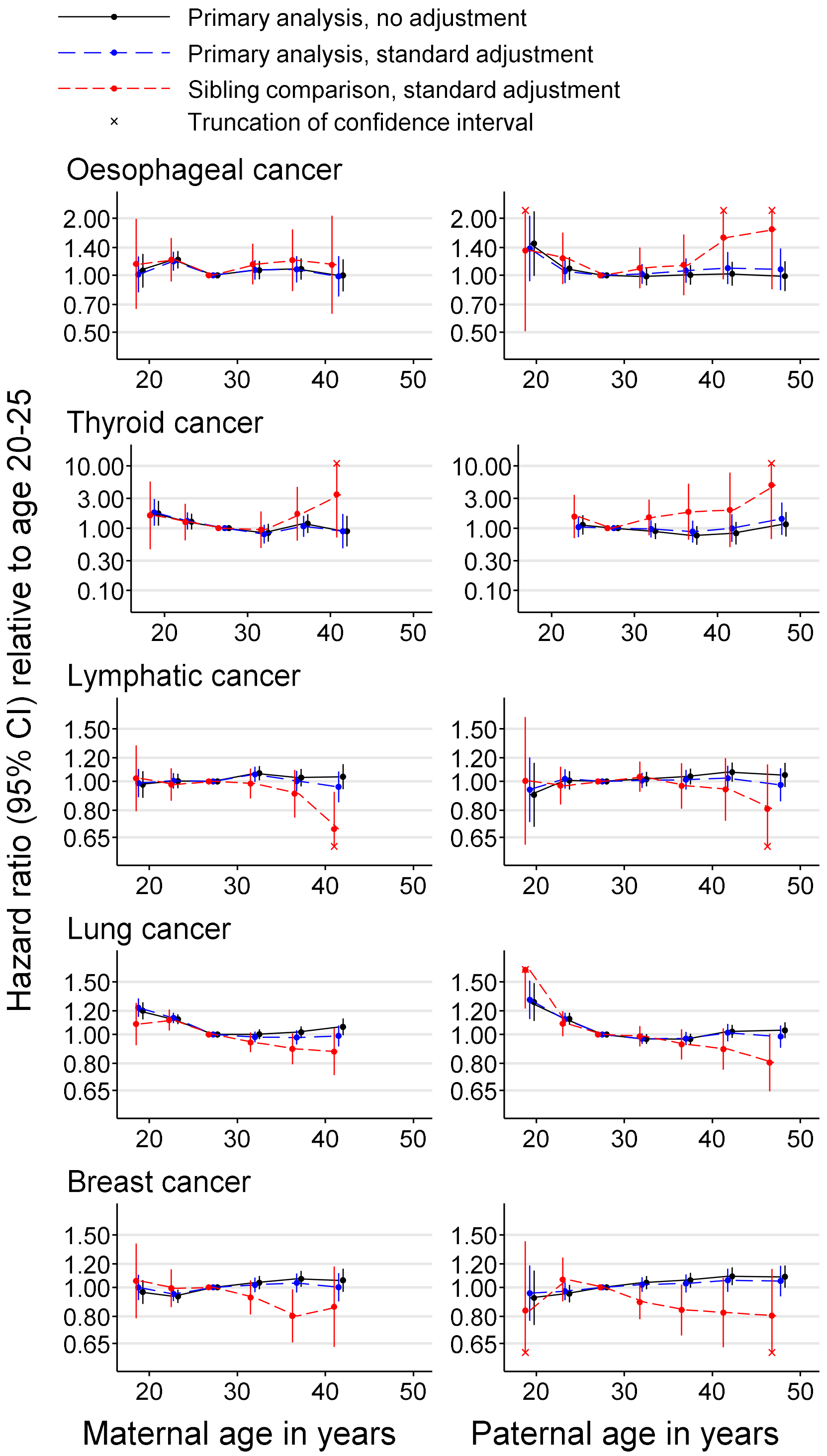

Figure S2 (continued). Hazard ratios for offspring mortality against classes of maternal or paternal age.

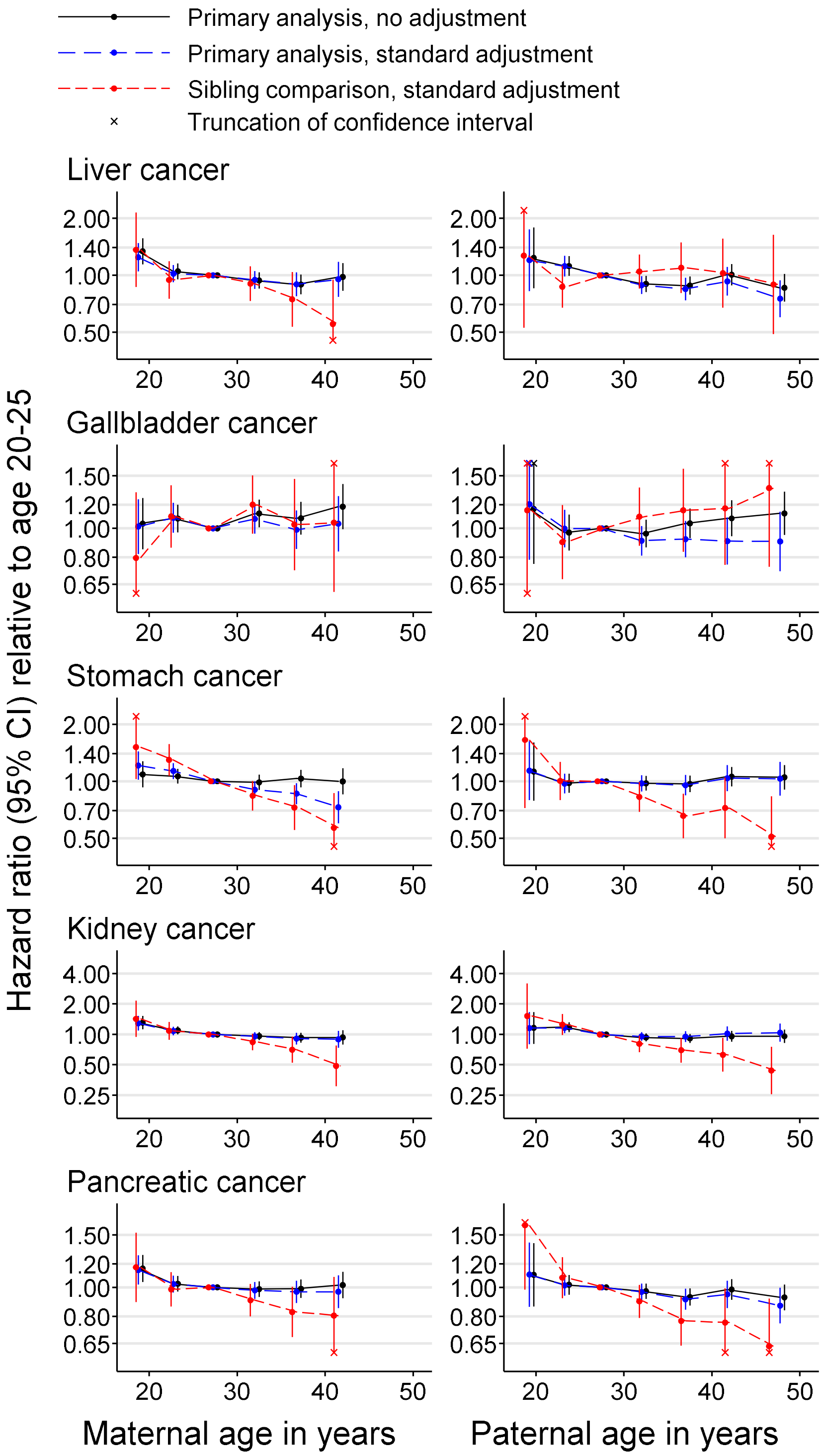

Figure S2 (continued). Hazard ratios for offspring mortality against classes of maternal or paternal age.

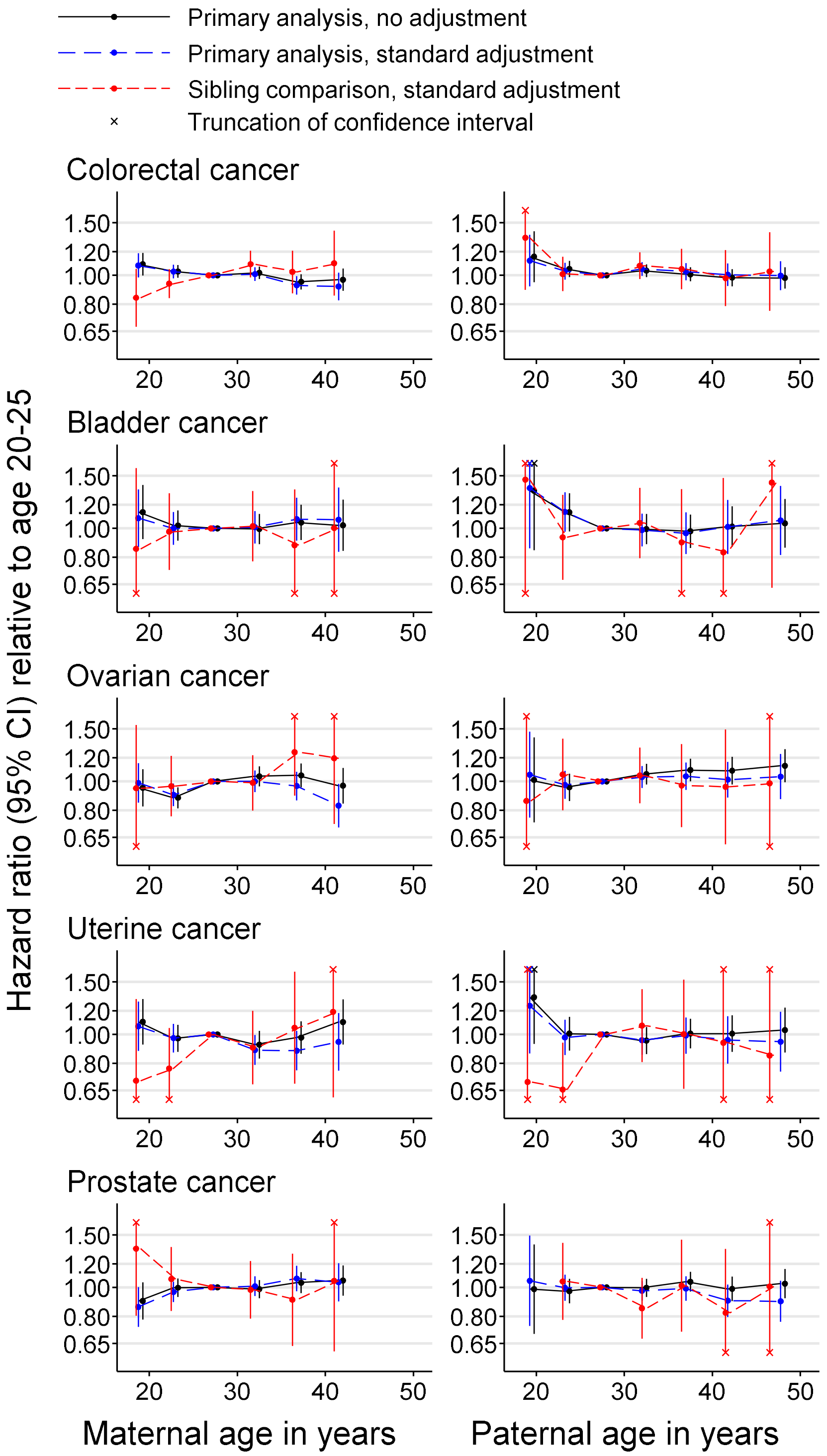

Figure S2 (continued). Hazard ratios for offspring mortality against classes of maternal or paternal age.

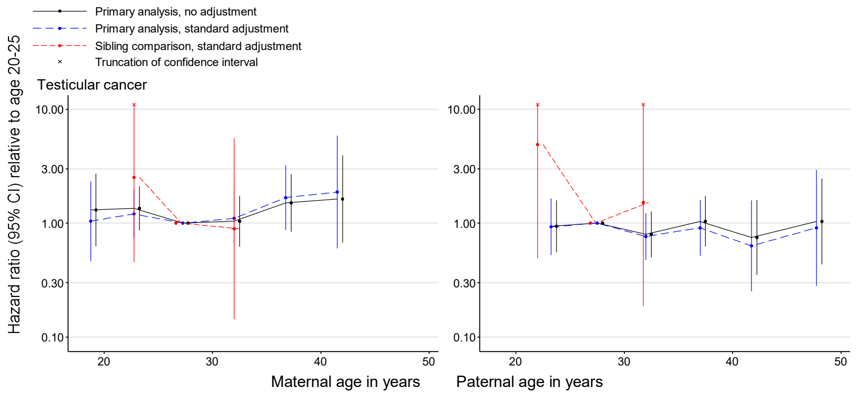

Supplementary Note. Comparison with Barclay & Myrskylä 2018.

Barclay and Myrskylä (B&M)[1] conducted similar analyses on the same starting dataset but got hazard ratios (HR) which were rather different from the most analogous models in our analysis, particularly for maternal age. Below, we consider how their analyses differ from the most closely corresponding all-cause analyses in ours and try to partially replicate their results by modifying our analyses. Their Model 1 was a standard Cox model (which we refer to as a primary analysis) and their Model 3 was a stratified Cox model (a sibling comparison analysis).

Differences between B&M and the current study:

1) B&M's Model 1 adjusted for the other parent's age (in categories) and offspring sex. The closest adjustment sets used with the primary analysis in the present analysis are (a) no adjustment and (e) offspring sex and date of birth, educational and occupational SEP in both parents, birth order and the other parent's age as a linear term. Their Model 3 adjusted for the other parents age (in categories), offspring sex and birth order. The closest adjustment set used with the sibling comparison analysis in the present analysis is (e) offspring sex and birth order.
2) B&M left-truncated follow-up at age 30; we did so at age 18. The aim in both studies was to investigate adult mortality, and mortality in early adulthood is very rare so this change is unlikely to have been influential.
3) B&M excluded people born before 1938; we used people born from 1932 (the earliest available). Their reasoning was that many people born in this early period would be incorrectly identified as first-born, because their older siblings were born before 1932 and thus not recorded in the data. Our reasoning was that while birth order may be an important covariate, people born in 1932-1938 provide a substantial proportion of recorded deaths and follow-up at older ages.
4) B&M used people born up to the end of 1960; we used all available data (people born up to 1987). We presume that their reasoning was that people born in 1960 were the last cohort who would already be 30 (their minimum age) by 1990 (the start of their follow-up). People born in 1960-1982 would turn 30 (and thus be followed up) sometime during their follow-up period of 1990-2012.
5) B&M followed up from January 1990; we followed up from July 1991. Prior to July 1991, there was missingness in the identification of parents which depended on the survival of the offspring (offspring who died were less likely to have identified parents).
6) B&M defined families by the identities of both parents; we used only the identity of the parent in question.
7) B&M excluded whole families which contained multiple births or where any child was born outside Sweden. We excluded only those individuals from multiple births. We did not have data on whether children were born outside Sweden (they may already have been removed) and did not replicate their removal of whole families containing multiple births.
8) B&M categorised birth order from 1st to ≥7th; we did so from 1st to ≥4th. We did not replicate this minor difference.
9) B&M categorised maternal age as 15-19, 20-24, 25-29 (ref), 30-34, 35-39, 40-44 and ≥45 years. Due to the rarity of very old mothers, we combined the oldest two categories. They categorised paternal age as 15-19, 20-24, 25-29 (ref), 30-34, 35-39, 40-44, 45-49, 50-54 and ≥55 years. Due to the rarity of very old fathers, we combined the oldest three categories. We did not replicate this difference.

The results suggest that differences between B&M's Model 1 and our primary analysis were mostly due to the adjustment variables, leading in our analysis to an increase in the additional hazard among the offspring of young parents and a reduction in the additional hazard among the offspring of older

parents. The linear models in Tables S2 and S3 suggest that adjustment for SEP was the most important difference. There may also have been an effect of our inclusion of later (younger) offspring in amplifying the risk associated with young parents.

In the comparison of B&M's Model 3 with our sibling comparison analyses, the omission of offspring born after 1960 attenuated the additional hazard in the offspring of young mothers partially, but not completely to the null finding of B&M. None of the changes applied individually replicated the increased hazard in the offspring of older mothers except a slight increase when follow-up began at January 1990 instead of July 1991. Nonetheless, the application of all of changes 1-6 approximately reproduced the results of B&M. This was not the case for paternal age, where the definition of families by the identities of both parents in isolation approximately reproduced the results of B&M, but when combined with the rest of changes 1-6, gave very different results.

Table 1. Results of models replicating the results of Model 1 in Barclay & Myrsklä (B&M).

	B&M Model 1	Adjustment e[a]	Change 1[a]	Change 2[a]	Change 3[a]	Change 4[a]	Change 5[a]	Change 6[a]	Changes 1-6[a]
Maternal age									
15-19	1.13 (1.10, 1.17)	1.19 (1.17, 1.21)	1.17 (1.15, 1.20)	1.18 (1.15, 1.20)	1.21 (1.18, 1.23)	1.15 (1.13, 1.18)	0.19 (1.17, 1.21)	1.19 (1.17, 1.21)	1.15 (1.13, 1.18)
20-24	1.05 (1.03, 1.07)	1.07 (1.06, 1.08)	1.06 (1.05, 1.08)	1.07 (1.06, 1.08)	1.07 (1.06, 1.08)	1.07 (1.06, 1.08)	1.07 (1.06, 1.08)	1.07 (1.06, 1.08)	1.06 (1.05, 1.08)
25-29	1.00	1.00	1.00	1.00	1.00	1.00	1.00	1.00	1.00
30-34	1.00 (0.99, 1.01)	0.98 (0.97, 0.99)	1.00 (0.99, 1.01)	0.98 (0.97, 0.99)	0.98 (0.97, 0.99)	0.98 (0.97, 0.99)	0.98 (0.97, 0.99)	0.98 (0.97, 0.99)	1.00 (0.99, 1.01)
35-39	1.02 (1.00, 1.04)	0.99 (0.97, 0.99)	1.02 (1.00, 1.03)	0.99 (0.98, 1.01)	0.98 (0.96, 1.00)	0.99 (0.97, 1.00)	0.99 (0.97, 0.99)	0.99 (0.97, 0.99)	1.02 (1.00, 1.04)
40-44[b]	1.05 (1.02, 1.08)	0.98 (0.96, 1.00)	1.04 (1.02, 1.06)	0.99 (0.96, 1.01)	1.01 (0.98, 1.03)	0.98 (0.96, 1.01)	0.98 (0.96, 1.00)	0.98 (0.96, 1.00)	1.05 (1.02, 1.08)
≥45	1.07 (0.99, 1.16)								
Paternal age									
15-19	1.13 (1.06, 1.21)	1.20 (1.16, 1.25)	1.12 (1.08, 1.16)	1.20 (1.15, 1.25)	1.21 (1.17, 1.27)	1.17 (1.12, 1.22)	1.21 (1.16, 1.25)	1.20 (1.16, 1.25)	1.13 (1.07, 1.18)
20-24	1.05 (1.03, 1.07)	1.08 (1.06, 1.09)	1.04 (1.03, 1.06)	1.08 (1.06, 1.09)	1.07 (1.06, 1.09)	1.08 (1.06, 1.09)	1.08 (1.06, 1.09)	1.08 (1.06, 1.09)	1.05 (1.03, 1.07)
25-29	1.00	1.00	1.00	1.00	1.00	1.00	1.00	1.00	1.00
30-34	0.98 (0.97, 1.00)	0.96 (0.95, 0.98)	0.99 (0.98, 1.00)	0.96 (0.95, 0.97)	0.96 (0.95, 0.97)	0.95 (0.94, 0.97)	0.96 (0.95, 0.97)	0.96 (0.95, 0.98)	0.98 (0.97, 1.00)
35-39	0.99 (0.97, 1.01)	0.96 (0.95, 0.97)	1.01 (0.99, 1.02)	0.96 (0.94, 0.97)	0.96 (0.94, 0.97)	0.94 (0.93, 0.96)	0.96 (0.95, 0.97)	0.96 (0.95, 0.97)	1.00 (0.98, 1.02)
40-44	1.00 (0.98, 1.02)	0.96 (0.95, 0.98)	1.04 (1.02, 1.05)	0.96 (0.94, 0.97)	0.97 (0.95, 0.99)	0.94 (0.92, 0.96)	0.97 (0.95, 0.98)	0.96 (0.95, 0.98)	1.02 (1.00, 1.04)
45-49[b]	1.03 (1.00, 1.07)	0.97 (0.94, 0.99)	1.07 (1.05, 1.10)	0.96 (0.94, 0.98)	0.97 (0.95, 0.99)	0.94 (0.91, 0.96)	0.97 (0.94, 0.99)	0.97 (0.94, 0.99)	1.05 (1.03, 1.08)
50-54	1.04 (0.98, 1.09)								
≥55	1.07 (0.97, 1.18)								
N	1,899,314	5,204,433	5,204,433	4,640,644	4,897,586	2,533,320	5,213,499	5,204,433	2,231,140
Deaths	157,328	293,470	293,470	282,615	213,662	263,843	296,979	293,470	186,014

[a]Maternal and paternal age analysed in separate analyses (with the same N).

[b]Includes higher age categories where results for these are not reported.

Changes are (1) adjustment, (2) age of left-truncation, (3) earliest DOB, (4) latest DOB, (5) date of left-truncation and (6) definition of families.

Table 2. Results of models replicating the results of Model 3 in Barclay & Myrsklä (B&M).

	B&M Model 3	Adjustment e[a]	Change 1[a]	Change 2[a]	Change 3[a]	Change 4[a]	Change 5[a]	Change 6[a]	Changes 1-6[a]
Maternal age									
15-19	1.01 (0.94, 1.08)	1.10 (1.06, 1.15)	1.08 (1.03, 1.13)	1.10 (1.06, 1.15)	1.12 (1.06, 1.18)	1.05 (1.00, 1.11)	1.08 (1.04, 1.13)	1.07 (1.03, 1.13)	0.99 (0.93, 1.06)
20-24	0.98 (0.95, 1.01)	1.04 (1.02, 1.06)	1.03 (1.01, 1.06)	1.05 (1.02, 1.07)	1.04 (1.02, 1.07)	1.03 (1.00, 1.05)	1.03 (1.01, 1.05)	1.04 (1.01, 1.06)	1.00 (0.97, 1.03)
25-29	1.00	1.00	1.00	1.00	1.00	1.00	1.00	1.00	1.00
30-34	1.00 (0.97, 1.03)	0.95 (0.93, 0.97)	0.96 (0.94, 0.99)	0.95 (0.93, 0.97)	0.96 (0.93, 0.98)	0.96 (0.93, 0.98)	0.97 (0.95, 0.99)	0.96 (0.94, 0.98)	0.99 (0.96, 1.02)
35-39	1.00 (0.95, 1.06)	0.92 (0.89, 0.95)	0.95 (0.91, 0.98)	0.92 (0.89, 0.95)	0.93 (0.89, 0.96)	0.93 (0.90, 0.97)	0.96 (0.93, 0.99)	0.93 (0.90, 0.96)	1.00 (0.94, 1.05)
40-44[b]	1.07 (0.98, 1.17)	0.90 (0.85, 0.94)	0.94 (0.88, 0.99)	0.90 (0.85, 0.94)	0.92 (0.87, 0.98)	0.91 (0.87, 0.96)	0.98 (0.93, 1.02)	0.91 (0.86, 0.95)	1.05 (0.96, 1.14)
≥45	1.15 (0.95, 1.39)								
Paternal age									
15-19	1.14 (0.99, 1.31)	1.17 (1.09, 1.26)	1.13 (1.04, 1.22)	1.19 (1.11, 1.29)	1.21 (1.12, 1.31)	1.12 (1.02, 1.22)	1.17 (1.07, 1.26)	1.13 (1.03, 1.23)	1.09 (0.97, 1.23)
20-24	1.02 (0.98, 1.06)	1.05 (1.03, 1.08)	1.03 (1.01, 1.06)	1.06 (1.03, 1.09)	1.06 (1.03, 1.09)	1.04 (1.01, 1.07)	1.04 (1.02, 1.07)	1.02 (0.99, 1.05)	0.98 (0.94, 1.02)
25-29	1.00	1.00	1.00	1.00	1.00	1.00	1.00	1.00	1.00
30-34	0.99 (0.96, 1.02)	0.95 (0.93, 0.97)	0.96 (0.94, 0.99)	0.95 (0.93, 0.97)	0.95 (0.93, 0.97)	0.97 (0.94, 0.99)	0.96 (0.94, 0.98)	0.97 (0.95, 1.00)	1.02 (0.99, 1.06)
35-39	0.96 (0.91, 1.01)	0.89 (0.87, 0.92)	0.91 (0.88, 0.95)	0.89 (0.87, 0.92)	0.89 (0.86, 0.92)	0.92 (0.89, 0.95)	0.93 (0.90, 0.96)	0.93 (0.90, 0.96)	1.02 (0.96, 1.08)
40-44	0.92 (0.86, 1.00)	0.86 (0.82, 0.89)	0.88 (0.84, 0.93)	0.86 (0.82, 0.90)	0.85 (0.81, 0.89)	0.90 (0.86, 0.94)	0.92 (0.88, 0.95)	0.91 (0.86, 0.95)	1.03 (0.95, 1.11)
45-49[b]	0.90 (0.81, 1.00)	0.81 (0.77, 0.86)	0.84 (0.78, 0.90)	0.81 (0.77, 0.86)	0.80 (0.75, 0.86)	0.86 (0.80, 0.92)	0.90 (0.85, 0.95)	0.87 (0.81, 0.93)	1.02 (0.92, 1.14)
50-54	0.81 (0.69, 0.95)								
≥55	0.87 (0.67, 1.12)								
N	319,749	379,710/384,208	379,710/384,208	363,655/367,962	278,560/281,337	322,010/326,608	392,646/396,990	354,380/360,415	214,879/218,286
Deaths	117,169	154,143/157,015	154,143/157,015	148,006/150,693	109,746/111,761	133,865/136,300	158,846/161,729	146,186/148,683	87,799/89,097

[a]Maternal and paternal age analysed in separate analyses (with slightly different N due to family definition; reported for paternal age / maternal age). N also differs with change 6 because within-family exposure discordance depends on the exposure parent even if family definition depends on both parents.
[b]Includes higher age categories where results for these are not reported.
Changes are (1) adjustment, (2) age of left-truncation, (3) earliest DOB, (4) latest DOB, (5) date of left-truncation and (6) definition of families.

References

1. Barclay K, Myrskylä M. Parental age and offspring mortality: Negative effects of reproductive ageing may be counterbalanced by secular increases in longevity. *Population Studies-a Journal of Demography.* 2018;72(2):157-173.
